# Protocol for a phase 2, partially blinded, randomized trial assessing the safety and efficacy of sorfequiline or bedaquiline in combination with pretomanid and linezolid in adult participants with newly diagnosed, drug-sensitive, smear-positive pulmonary tuberculosis (NC-009)

**DOI:** 10.1186/s13063-025-09413-5

**Published:** 2026-01-06

**Authors:** M. Olugbosi, M. Beumont, L. Lombard, J. Nedelman, J. Timm, T. Black, R. Bruning-Barry, D. Hickman, A. Lombardi, M. Betteridge, E. Egizi, L. Marcopulos, J. Henderson, S. Seidel, S. Foraida, M. Benhayoun, E. Sun

**Affiliations:** 1 TB Alliance, Sandton, South Africa; 2 TB Alliance, New York City, USA; 3 RTI International, Research Triangle Park, USA

**Keywords:** Sorfequiline, Bedaquiline, Pretomanid, Linezolid, Drug-sensitive TB, Clinical trial

## Abstract

**Introduction:**

In 2023, tuberculosis (TB) returned to being the world’s leading cause of death from a single infectious agent. Current standard of care for drug-sensitive tuberculosis (DS-TB) treatment has a long duration with risk of poor compliance and outcomes and increased risk of development of resistant strains. Sorfequiline (S) is a second-generation diarylquinoline with the potential to contribute both to increased efficacy and improved safety and to a shorter TB treatment regimen for both DS-TB and drug-resistant DR-TB.

**Methods and analysis:**

NC-009 is a phase 2, multicentre, partially blinded, randomized clinical trial conducted in five treatment arms, including three doses of sorfequiline in combination with pretomanid and linezolid for 8 weeks followed by 7 or 18 weeks of HR depending on the participant meeting criteria to stop treatment at week 15. There is also a BPaL arm and standard of care arm, both for a 26-week treatment duration. The study population is smear-positive, DS-TB. The primary objective of the study is to determine the optimal dose of sorfequiline to move forward to a potential phase 3 study based on efficacy and safety data. The study is being conducted in Georgia, South Africa, Tanzania, Uganda, and the Philippines in accordance with ICH-GCP and after approval of all relevant country health authorities and ethics committees.

**Discussion:**

NC-009 is a first in-patient, dose-ranging, phase 2 trial of sorfequiline, with an innovative design combining ph2a, ph2b, and ph2c elements that simultaneously allow for dose selection, preliminary evaluation of treatment duration, robust collection of safety data, evaluation of drug-drug interaction with antiretroviral medications, and pharmacokinetic assessment of study drugs. The study is currently ongoing.

**Trial registration number {4}:**

ClinicalTrials.gov NCT 06058299. Registered on 09 September 2023.

**Supplementary Information:**

The online version contains supplementary material available at 10.1186/s13063-025-09413-5.

## Strengths and limitations of this study

- This is a unique study that combines dose selection for a next-generation diarylquinoline (three doses of sorfequiline evaluated) using efficacy and safety data, plus evaluation of potential duration of treatment based on ability to stop treatment at week 15 based on meeting certain criteria.
- This study also evaluates the potential drug–drug interaction of sorfequiline on dolutegravir and tenofovir.
- The study is not powered to compare sorfequiline and bedaquiline, both in combination with pretomanid and linezolid.

## Introduction

### Background and rationale {6a}

Tuberculosis (TB) is a preventable and usually curable disease. Yet in 2023, TB returned to being the world’s leading cause of death from a single infectious agent, following 3 years in which it was replaced by coronavirus disease (COVID-19) [1].

Existing drug-sensitive TB (DS-TB) treatment regimens are efficacious but lengthy in duration and involve multidrug therapy combinations, i.e. an intensive phase of 2 months of isoniazid (H), rifampicin (R), pyrazinamide (Z), and ethambutol (E) followed by a continuation phase of 4 months of isoniazid and rifampicin (HRZE/HR); this treatment regimen was first introduced as the standard of care more than 40 years ago [2]. Long and complex treatments lead to high rates of non-adherence, which often result in unfavourable outcomes, emergence of drug resistance, continued spread of disease, and increased mortality. Drug-drug interactions caused by rifampicin’s potent induction of CYP enzymes are another major challenge with the HRZE/HR regimen, particularly for women on hormonal contraception and TB participants co-infected with HIV taking antiretroviral therapies (ARVs). While a 4-month rifapentine-based regimen for treatment of pulmonary DS-TB is now recommended by the WHO as a possible alternative to HRZE/HR, but with very limited global roll-out [3], there is a need for new TB drugs and drug regimens that are efficacious, safe, well-tolerated, simpler, and that can further shorten the overall treatment duration, thereby improving adherence and patient outcomes.

The combination of bedaquiline, a first-generation diarylquinoline, with pretomanid (a nitroimidazole), and linezolid (an oxazolidinone) administered for 26 weeks has been extensively studied and is referred to as the BPaL regimen. The BPaL regimen demonstrated robust efficacy (> 90% relapse-free cure) in adults with pulmonary XDR-TB and TI/NR MDR-TB (pre-WHO 2021 definitions) when linezolid was dosed at 1200 mg daily [4]. When the linezolid dose was decreased to 600 mg daily, efficacy was retained, and the regimen demonstrated improved tolerability [5]. BPaL (plus moxifloxacin in patients with fluoroquinolone-susceptible TB) for 6 months is currently recommended for patients with drug-resistant TB (DR-TB) sensitive to all components of the regimen [6]. The high potency of the BPaL regimen has the potential to also shorten treatment for DS-TB [7].

Sorfequiline (S) is a second-generation diarylquinoline selected for development based on initial positive nonclinical results. In vitro and in vivo studies have shown the increased potency of sorfequiline over bedaquiline. The sorfequiline minimum inhibitory concentration (MIC) against a phylogenetically diverse panel of 96 clinical strains was approximately 10-fold lower than the corresponding bedaquiline MICs, and in the acute and chronic mouse models of TB, sorfequiline was superior to bedaquiline when administered as monotherapy or in combination with PaL. Crucially, the activity of sorfequiline against the most common type of bedaquiline-resistant mutant (Rv0678 mutant) was similar to that of bedaquiline against a wild-type strain, both in vitro and in the mouse model [8]. Based on in vitro cardiac potassium channel current inhibition screening studies (hERG assay) and in vivo cardiovascular safety assessments, sorfequiline has shown a reduced risk for QTc prolongation compared with bedaquiline [9]. Sorfequiline thus has the potential to contribute both to increased efficacy and improved safety and to a shorter TB treatment regimen for both DS-TB and DR-TB.

## Objectives {7}

List of primary, key secondary, secondary, and exploratory objectives and associated endpoints are described in Table 1.

**Table 1:** List of primary, key secondary, secondary, and exploratory objectives and associated endpoints

Objectives	Endpoints
**Primary**	
To evaluate the efficacy of three dose levels of sorfequiline in combination with pretomanid and linezolid, relative to 2HRZE, during 8 weeks of treatment in adult participants with newly diagnosed, smear-positive, pulmonary DS-TB as measured by time to stable sputum culture conversion to negative status	Time to stable sputum culture conversion to negative status using data from weekly cultures through 8 weeks of treatment
**Key secondary**	
To evaluate the safety and efficacy at 26 weeks after the EOT of B-Pa-L relative to 2HRZE/4HR in adult participants with newly diagnosed, smear-positive, pulmonary DS-TB	Proportion of participants with a favourable outcome at 26 weeks after the EOT
**Secondary**	
To evaluate the efficacy of three dose levels of sorfequiline in combination with pretomanid and linezolid in adult participants with newly diagnosed, smear-positive, pulmonary DS-TB, as measured by meeting the criteria to stop treatment at week 15	Proportion of participants who meet the criteria to stop treatment at week 15
To evaluate the safety and efficacy of three dose levels of sorfequiline in combination with pretomanid and linezolid at 26 weeks and 52 weeks after end of treatment relative to 2HRZE/4HR for the overall arms and key subgroups by stratification factors and total treatment duration in adult participants with newly diagnosed, smear-positive, pulmonary DS-TB	Proportion of participants with a favourable outcome at 26 weeks and 52 weeks after the EOT
To evaluate the safety and efficacy at 52 weeks after the EOT of B-Pa-L relative to 2HRZE/4HR in adult participants with newly diagnosed, smear-positive, pulmonary DS-TB	Proportion of participants with a favourable outcome at 52 weeks after the EOT
To evaluate the efficacy of three dose levels of sorfequiline in combination with pretomanid and linezolid, B-Pa-L, and 2HRZE/4HR at 26 weeks and 52 weeks after the EOT, as measured by relapse rates in participants that enter follow up with a favourable response at the EOT for the overall arms and key subgroups by stratification factors and total treatment duration	Relapse rates at 26 weeks and 52 weeks after the EOT
To evaluate the efficacy of B-Pa-L, relative to 2HRZE, during 8 weeks of treatment in adult participants with newly diagnosed, smear-positive, pulmonary DS-TB as measured by time to stable sputum culture conversion to negative status	Time to stable sputum culture conversion to negative status using data from weekly cultures through 8 weeks of treatment
To assess the bactericidal activity over 2 weeks of three dose levels of sorfequiline in combination with pretomanid and linezolid or B-Pa-L, relative to 2HRZE, in adult participants with newly diagnosed, smear-positive, pulmonary DS-TB	*BA*_TTP_ (1–15) as determined by the rate of change in TTP over days 1 to 15 of treatment, represented by the model-fitted log(TTP) as calculated by the regression of the observed log(TTP) counts over time
To assess the bactericidal activity over 8 weeks of three dose levels of sorfequiline in combination with pretomanid and linezolid or B-Pa-L, relative to 2HRZE, in adult participants with newly diagnosed, smear-positive, pulmonary DS-TB	*BA*_TTP_ (1–56) as determined by the rate of change in TTP over 8 weeks of treatment, represented by the model-fitted log(TTP) results as calculated by the regression of the observed log(TTP) results over time
To evaluate stable sputum culture conversion to negative status over time during the treatment period	Proportion of participants with stable sputum culture conversion to negative status at weeks 4, 6, 8, 12, 15, 20, and 26
To assess the safety and tolerability of the three dose levels of sorfequiline in combination with pretomanid and linezolid or B-Pa-L regimens at different time points, relative to 2HRZE/4HR, in adult participants with newly diagnosed, smear-positive, pulmonary DS-TB	Incidence of TEAEs by severity, drug relatedness, seriousness, and whether they lead to early discontinuation and death; ECG, vital signs, and quantitative and qualitative clinical laboratory result measurements; changes in ophthalmic exam for visual acuity; and changes noted in peripheral neuropathy, including observed changes from baseline at weeks 8 and 26 and 26 weeks after the EOT
To evaluate the systemic exposure of sorfequiline, bedaquiline, pretomanid, and linezolid	Plasma concentrations of sorfequiline, bedaquiline, pretomanid, linezolid, and selected metabolites from sparse samples in all participants assigned those treatments and from an intensive profile on day 15 in a sub-group of participants
**Exploratory**	
To assess if changes in measurements of biomarker assays through the course of treatment and the post-treatment follow-up period are associated with treatment outcome	Change from baseline in biomarker assay through the course of treatment and the post-treatment follow-up period relative to treatment outcome
To correlate various endpoints measured with MGIT with AFB smear and exploratory biomarkers at day 1 and weeks 4, 8, 15, and 26	Parameters derived from modelling various endpoints measured with MGIT (e.g. MGIT negative [yes/no], quantitative outcomes) relative to AFB smear and exploratory biomarkers at day 1 and weeks 4, 8, 15, and 26
To correlate MGIT culture results at weeks 2, 4, and 8 with favorable outcome at 26 weeks and 52 weeks after the EOT	Parameters derived from modelling MGIT culture results at weeks 2, 4, and 8 (e.g. MGIT negative [yes/no], quantitative outcomes) relative to favourable outcome at 26 weeks and 52 weeks after the EOT
To explore predictors of MGIT-negative culture at week 8 of treatment	MGIT negative at week 8 of treatment (yes/no) relative to key baseline characteristics
To explore the population PK and exposure–response relationships of sorfequiline, bedaquiline, pretomanid, and linezolid	Population-PK models, summary metrics of exposure (e.g. AUC) derived from those models, and models for relationships between exposure and efficacy and safety outcomes
To explore the systemic exposure of dolutegravir and tenofovir when co-administered with three dose levels of sorfequiline in combination with pretomanid and linezolid, B-Pa-L, or 2HRZE	Trough concentrations of dolutegravir and tenofovir from participants living with HIV-assigned treatments
To explore the impact on quality of life of three dose levels of sorfequiline in combination with pretomanid and linezolid, B-Pa-L, and 2HRZE/4HR	Time to improvement of quality of life measurements and proportion of participants with improved measurements on quality of life at different time points during and after treatment

## Trial design {8}

### Intervention description {11a}

Treatment will be administered by the site to the participant at scheduled trial visits. In between scheduled site visits, the participants will be responsible for the administration of their own investigation medicinal product (IMP).

- *First three treatment arms*: A total of 25 mg/50 mg/100 mg of sorfequiline + 200-mg pretomanid + 600-mg linezolid for 8 weeks (TP1) followed by 7 weeks or 18 weeks of HR based on participant meeting criteria to stop treatment at week 15 (TP2)
- *Fourth treatment arm*: A total of 200-mg bedaquiline + 200-mg pretomanid + 600-mg linezolid for 1 st 8 weeks (TP1) followed by 100-mg bedaquiline + 200-mg pretomanid + 600-mg linezolid for 18 weeks (TP2)
- *Fifth treatment arm*: HRZE for 8 weeks followed by HR for 18 weeks

This is a phase 2, multicentre, partially blinded, randomized clinical trial where at least 300 participants with DS-TB who meet all the inclusion criteria and none of the exclusion criteria, aged 18 to 65, will be randomized to receive 1 of the 5 active treatment regimens (at least 60 participants per regimen). Participants will be randomized equally, using an interactive response technology (IRT) that stratifies based on country and severity of disease (AFB 3+ and/or bilateral cavitation) to 1 of the 5 daily treatment regimens.

The trial consists of the following periods:

- *Screening period*: Screening visit up to 11 days prior to randomization (day 1)
- *Treatment period 1 (TP1):* Day 1 through week 8 (SPaL or BPaL or HRZE)
- *Treatment period 2 (TP2)*
  ◦ Week 9 through week 15 (participants in the SPaL arms that meet criteria for early completion of treatment)
  ◦ Week 9 through week 26 (participants in the HRZE/HR or BPaL arms and participants in the SPaL arms who do not meet criteria for early completion of treatment).
- *Post end-of-treatment (EOT) follow-up period*: 52 weeks after EOT

After receiving 8 weeks of treatment, participants in the SPaL arms and in the control arm (HRZE/HR) will continue treatment with HR, and participants randomized to BPaL will continue treatment with BPaL. Treatment completion will be allowed at week 15 in participants randomized to the SPaL arms, if the criteria below are met:

- Week 8 sputum mycobacterial growth indicator tube (MGIT) culture is negative.
- The participant has no TB-related symptoms by week 15.

If the MGIT result is MTB positive and/or there are still TB symptom(s), participants will continue to receive HR (in the SPaL arms) and will complete a total of 26 weeks of treatment.

Note: The inability to produce sputum will be considered as a negative MGIT culture result and therefore can be used to determine the participants’ eligibility to complete treatment at week 15.

For all participants except those on HRZE/HR, blood samples to assess the pharmacokinetics (PK) of study drugs are collected weekly pre-dose and at 1, 3, and 5 h post-dose at day 15 and week 8. Participants at some sites may volunteer for 24-h intensive sampling at day 15. In addition, pre-dose samples are collected at days 1 and 8 and weeks 4 and 8 to assess the PK of tenofovir and dolutegravir.

**Fig. 1:**
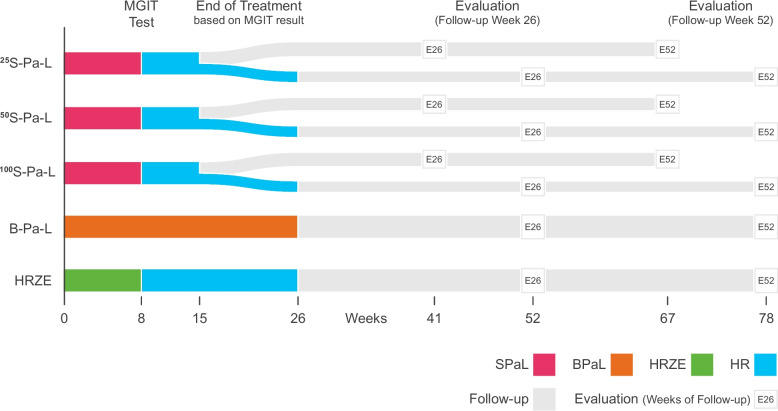
NC-009 Trial Design

See Fig. 1 for NC-009 Trial Design.

### Retreatment {30}

Participants randomized to the 3 SPaL arms and BPaL arm who relapse or experience treatment failure (see definition of relapse and treatment failure in Table 2) will be retreated with HRZE/HR, provided that drug susceptibility data do not reveal emergence of resistance or the participant has a contraindication for receiving HRZE/HR, in which case they will be referred to the local national TB programme (NTP) for further management. Participants who are retreated will be followed for 26 weeks after the end of their retreatment.

**Table 2:** Study outcome definitions

	**Definition**
Treatment failure	**Treatment failure diagnosed *by the end of treatment* IF ≥ 2 of A, B, or C are satisfied OR if D is satisfied** **A. Clinical disease persistence** Persistent TB symptoms or signs without an alternative explanation **B. Chest X-ray progression** Abnormalities that are compatible with active TB disease (cavitation, infiltrates, consolidation) with clear evidence of no improvement or progression compared with start of treatment without an alternative, more likely explanation C. Unconfirmed positive MGIT sputum sample result after having achieved culture-negative status (defined as two negative culture results at least 7 days apart) **D. Confirmed MTB-positive sputum culture** Failure to attain culture-negative status by the end of treatment or failure to maintain culture-negative status (defined as two positive culture results at least 7 days apart after achieving culture negative status)
Relapse or reinfection	*Relapse or reinfection diagnosed during follow up* in participants with culture-negative status by the EOT, *IF ≥ 2 of A, B, or C are satisfied, OR if D is satisfied* **A. Clinical disease progression** New or recurrent TB symptoms or signs after complete resolution at the EOT without an alternative, more likely explanation **B. Chest X-ray progression** Abnormalities that are compatible with active TB disease (cavitation, infiltrates, consolidation) with clear evidence of progression compared with the EOT without an alternative, more likely explanation **C. Microbiological evidence of recurrence** Sputum sample taken after the EOT is smear positive OR GeneXpert positive (if > 26 weeks after EOT) OR unconfirmed MGIT MTB-positive culture **D. Confirmed MTB-positive sputum culture** Sputum culture MTB positive on two consecutive samples, at least 7 days apart
Stable culture conversion	Two negative results (at least 7 days apart without an intervening MTB positive). If the first negative result occurs at week 8, the week 9 culture result can be used for the confirmatory negative result
Lost to follow-up	Should a participant not be reachable to attend trial visits, the following should be conducted and documented in the site trial records before confirming a participant is lost to follow-up: •Three documented telephone contact attempts (participant and/or relative) •Letter to be sent to the participant’s physical address via courier or a similar process (adapted per country-specific requirements/circumstances) If no feedback is received within 30 days of courier confirmation of letter delivery to and receipt by the participant, then the participant may be considered lost to follow-up

In the case of treatment failure or relapse in participants randomized to the control arm (HRZE/HR), the participant will be referred to the local NTP for further management, and the treating physicians will be provided with the DST results and medical report from the investigator, should an individualized regimen be indicated.

Participants who early discontinue from the study for reasons other than relapse/reinfection or treatment failure, where possible, would complete an early discontinuation visit and be referred to the local NTP. No additional follow-up visits are required except where unscheduled visits are needed for ongoing AEs that led to discontinuation from the trial and for pregnancies.

TB Alliance certifies that it has liability insurance coverage for itself and will provide an associated certificate upon request. The insurance does not relieve the investigators of the obligation to maintain their own liability insurance as required by applicable law. TB Alliance does not assume any obligation for the medical treatment of other injuries and illnesses, but in certain instances, compassionate support is provided to participants as required.

## Methods and analysis

### Study setting {9}

The trial will be performed at multiple centres globally, including Georgia, the Philippines, South Africa, Tanzania, and Uganda. These are a total of 22 clinical trial units recruiting participants from TB clinics in catchment areas. See Table 3 for the list of countries, cities, and site names (Table 4).

**Table 3:** List of countries, cities and site names

Country	City	Site name
Georgia	Tbilisi	National Center for Tuberculosis and Lung Diseases
Philippines	Manila	Tropical Disease Foundation
		Lung Center of Philippines
		Care Clinical Trial Group, Inc.
South Africa	Brits	Madibeng Centre for Research
	Cape Town	Desmond Tutu Health Foundation
		TASK Clinical Trials
		University of Cape Town Lung Institute
	Durban	Enhancing Care Foundation
		TB and HIV Investigative Network
	East London	Synergy Biomed Research Institute
	George	TASK Eden
	Johannesburg	Clinical HIV Research Unit, Helen Joseph Hospital
	Klerksdorp	Prenatal HIV Research Unit, Tshepong Hospital
	Port Elizabeth	Isango Lethemba TB Research Unit
	Pretoria	Setshaba Research Centre
	Rustenburg	The Aurum Institute
Tanzania	Mbeya	National Institute for Medical Research, Mbeya
	Moshi	Kilimanjaro Clinical Research Institute
	Mwanza	National Institute for Medical Research, Mwanza
Uganda	Kampala	Joint Clinical Research Institute
		Case Western Reserve University, Makerere University

**Table 4:** List of ethical boards and regulatory authorities

Country	RA: regulatory authority EC: Ethics committee	Name of RA or EC	Reference number	Approval
Georgia	Local EC	JSC National Centre of Tuberculosis and Lung Diseases Independent Ethics Committee	817/01–17	26 Mar 2023
Georgia	RA	LEPL Regulation Agency for Medical and Pharmaceutical Activities of Ministry of Internally Displaced Persons from the Occupied Territories, Labor, Health and Social Affairs of Georgia	No. 000924	13 May 2023
Philippines	Local EC	Makati Medical Centre Institutional Review Board	MMCIRB20 23–083	16 Aug 2023
Philippines	Local EC	Lung Centre Philippines Institutional Review Board	LCP-CT-015–2023	4 Jul 2023
Philippines	Local EC	Batangas Medical Centre Research Ethics Review Committee	BatMC RERC 2024-002	2 Jul 2024
Philippines	Central EC	Single Joint Research Ethics Board	SJREB-2023-62	16 Aug 23
Philippines	RA	Philippine Food and Drug Administration	2023-CT0791	9 Oct 2023
South Africa	Central EC	Wits Health Research Ethics Committee	230305	23 Jun 2023
South Africa	Local EC	University of Cape Town, Faculty of Human Sciences Human Research Ethics Committee	280/2023	7 Aug 2023
South Africa	Local EC	University of Cape Town, Faculty of Human Sciences Human Research Ethics Committee	699/2023	28 Sep 2023
South Africa	Central EC	Pharma-Ethics Health Research Ethics Committee	240626487-62	29 Jul 2024
South Africa	RA	South African Health Products Regulatory Authority	20230301	19 Apr 2023
Tanzania	Local EC	Mbeya Medical Research and Ethics Review Committee	SZEC – 2439/R.A/V.1/175	30 Mar 2023
Tanzania	Local EC	Kilimanjaro Christian Medical Centre Research Ethics Committee	Proposal number 1421	21 Jun 2023
Tanzania	Central EC	National Institute for Medical Research	N/A	26 May 2023
Tanzania	RA	Tanzania Medicines and Medical Devices Authority	BC.69/96/64/3	14 Sep 2023
Uganda	Local EC	Joint Clinical Research Centre Research Ethics Committee	JCRC-2023-43	3 May 2023
Uganda	Central EC	Uganda National Council for Science and Technology	HS2928ES	7 Aug 2023
Uganda	RA	Uganda National Drug Authority	CTC 0248/2023	9 Aug 2023

### Eligibility criteria {10}

The key inclusion and exclusion criteria are listed below:

#### Inclusion criteria

- Signed informed consent
- DS-TB is defined as sensitive to rifampicin and isoniazid by rapid sputum-based test AND either newly diagnosed for TB or has a history of being untreated for at least 3 years after cure from a previous episode of TB.
- Of non-childbearing potential or using effective birth control methods
- Body weight ≥ 35 kg

#### Exclusion criteria

- Karnofsky score < 60 at screening
- Any evidence of extrapulmonary TB
- Cardiovascular or QT prolongation risk factors
- Pregnant or breastfeeding
- Any of the following lab toxicities:
  ◦ Platelets < 100,000/mm^3^
  ◦ Creatinine > 1.3 × ULN
  ◦ Haemoglobin < 9.5 g/dL or < 95 g/L
  ◦ Absolute neutrophil count < 800/mm^3^
  ◦ Serum potassium less than the lower limit of normal for the laboratory
  ◦ ALT and/or AST ≥ 2.5 × ULN
  ◦ Total bilirubin ≥ 1.6 × ULN
  ◦ Direct bilirubin > 1 × ULN
  ◦ Haemoglobin A1c ≥ 8.0%
  ◦ Total lipase ≥ 1.5 × ULN
  ◦ Total amylase ≥ 1.5 × ULN
  ◦ CPK > 3 × ULN (if > 3 × ULN, enquire about the participant’s recent strenuous activity and consider repeating the test within the screening window)
  ◦ TSH > 1 × ULN
- For participants living with HIV only as follows:
  ◦ CD4 + count < 200 cells/μL
  ◦ WHO clinical stage 4 HIV disease
  ◦ Participant does not agree to use DTG/TFV/3TC during the trial if ARV therapy is indicated and randomized to the sorfequiline or the BPaL regimen.
  ◦ If initiation of ARV therapy is indicated, participants who are known to be intolerant, non-responsive to DTG/TFV/3TC or have DTG/TFV/3TC as a contraindication.

### Consent or assent {26a}

Informed consent forms are approved by the relevant ethics committee and regulatory authority. Written informed consent will be obtained from each screened participant, and the process is conducted in the participants’ preferred local languages in each country. In cases where a participant is illiterate, an impartial witness will be present throughout the informed consent process to ensure that the information in the consent form was accurately explained to and understood by the participant, and that informed consent was freely given by the participant.

### Additional consent provisions for collection and use of participant data and biological specimens {26b}

Additional consents to be signed by participants are as follows:

- Pharmacokinetic sampling (for participants willing to participate in 24 h of PK sample collection at day 15, PK subgroup)
- Biostorage for the following:
  ◦ The storage of all unused blood, sputum, and urine samples (as available) for long-term storage
  ◦ Additional blood and urine samples for exploratory research will be collected at day 1 and weeks 4, 8, 15, and 26.
- Pharmacogenetic testing for participants not randomized to the HRZE/HR arm to collect a blood sample at day 1 for possible exploratory pharmacogenetic testing.

## Interventions

### Explanation for choice of comparator {6b}

The 6-month treatment regimen is composed of four first-line TB medicines—HRZE/HR per weight band. This regimen is well known and has been widely adopted worldwide for decades; while using it, about 85% of participants will have a successful treatment outcome [1]. This regimen is based on seminal TB treatment studies conducted by the British Medical Research Council in the second half of the twentieth century [10].

### Intervention modifications {11b}

At no time should the participant be treated with a single agent. If any of the components other than linezolid need to be interrupted, the entire regimen must be interrupted. Dose adjustments are not allowed for sorfequiline, bedaquiline, pretomanid, or HRZE/HR. Linezolid can be reduced from 600 to 300 mg or temporarily interrupted or permanently discontinued. No minimum number of doses of linezolid is specified in the protocol.

During TP1 and 2, the full regimen can be interrupted for up to 14 and 28 cumulative doses for drug-related toxicities respectively.

### Strategies to improve adherence to interventions {11c}

Participants are reminded at each dispensing visit about the importance of adherence and compliance to the IMP. Site staff encourage participants to contact them between visits if they have any questions about their medications or if they are feeling unwell. The site contact details (24 h) are provided to the participant at the screening visit via a ‘Participant Contact Card’.

### Concomitant care that is permitted or prohibited during the trial {11d}

All therapies (prescriptions or over-the-counter medications, including vitamins and herbal supplements) different from the trial drugs are recorded in the concomitant therapy section of the Direct Data Capturing (DDC) platform.

#### Prohibited concomitant medications

The following therapies are not allowed during the trial: All medicinal products used to treat pulmonary TB, isoniazid prophylaxis as treatment preventive therapy (TPT) preventative for participants living with HIV, and monoamine oxidase inhibitors (due to linezolid, applicable to SPaL and BPaL regimens).

#### Concomitant medication to be avoided

The following concomitant medications should be avoided during and for 14 days after treatment with IMP to prevent possible drug interactions with the IMP: any drug known to be hepatotoxic (applicable for all treatment regimens), e.g. NSAIDs and acetaminophen; any drug known to prolong QTc interval (applicable to SPaL and BPaL regimens), e.g. chloroquine and amiodarone; any drug known to induce significant myelosuppression (due to linezolid, applicable to SPaL and BPaL regimens), e.g. chloramphenicol; systemic use of strong CYP3A4 inhibitors, for more than 14 consecutive days (applicable to SPaL and BPaL regimens), e.g. azole antifungals; systemic use of strong and moderate CYP3A4 inducers should be avoided 14 days before and during treatment (applicable to SPaL and BPaL regimens), e.g. phenytoin and carbamazepine; serotonergic antidepressants (SPaL and BPaL regimens), e.g. fluoxetine and paroxetine; and strong P-gp inhibitors for more than three consecutive days (applicable to SPaL and BPaL regimens), e.g. cyclosporine.

### Provision of posttrial care {30}

Participants who early discontinue from the study for reasons other than relapse/reinfection or treatment failure, where possible, would complete an early discontinuation visit and be referred to the local NTP. No additional follow-up visits are required except where unscheduled visits are needed for ongoing adverse events (AEs) that led to discontinuation from the trial and for pregnancies.

### Outcomes {12}

See Table 1 for study objective and relevant outcomes/end points. See Table 2 for study outcome definitions.

### Participant timelines {13}

See trial flow chart in NC-009 protocol (supplementary materials).

### Sample size {14}

Sample size for this trial will be at least 60 participants per treatment regimen. Sample size assumptions below are selected to be conservative to achieve adequate power given expected minimal dropout prior to week 8.

Based on data from previous trials of DS-TB, it is assumed that the probability of participants achieving stable sputum culture conversion to negative status by week 8 and TP1 in the control regimen (HRZE/HR) is 0.50 [11, 12]. The number of events and power required is based on comparison of the primary endpoint, i.e. time to stable sputum culture conversion to negative status using data from weekly cultures through week 8 and TP1.

The trial will require approximately 76 events (stable sputum culture conversion to negative status) among approximately 120 randomized participants. This number ensures that a two-sided *α* = 0.05 logrank test procedure will have 80% power when the true hazard ratio of sorfequiline vs. HRZE is 2.0 and 90% power when the hazard ratio is 2.2. It is assumed that calculations are sufficiently conservative to ensure that the required number of events will be observed by the time the analysis is conducted using week 8 data. To minimize type 1 error, the comparisons for inference will be ordered starting with the highest sorfequiline dose regimen.

### Recruitment—strategies for achieving adequate participant randomization to reach target sample size {15}

Participating sites foster and maintain positive working relationships with the local TB clinics in their areas via the community engagement (CE) teams and activities. Before a trial commences, the site CE team meets with and informs community stakeholders, e.g. Community Advisory Board (CAB), TB clinic nurses, and doctors about the trial. When the trial starts, the TB clinics identify patients who have been newly diagnosed with DS-TB, and they inform the site CE contact person. The CE contact person talks with the patient to find out if they would like to know more about the trial, and if they agree, they invite them to the site. The CE team arranges transportation for the participant from their home or TB clinic to the site while they still test positive for TB. This process facilitates a good relationship between CAB, TB clinics, and trial sites and, as a consequence, hopefully good recruitment to the trial.

### Who will be blinded {17a}

The trial is partially blinded, i.e. sorfequiline and bedaquiline will be blinded during the first 8 weeks of trial treatment; participants randomized to the sorfequiline or bedaquiline arms will receive open-label pretomanid and linezolid. After the week 8 visit, participants will be unblinded if they are randomized to sorfequiline or bedaquiline, but the dose of sorfequiline remains blinded throughout the study. Participants randomized to the HRZE/HR arm will receive open-label IMP. Only the unblinded statistician will have access to unblinded data.

### Blinding (masking): emergency unblinding {17b}

The blind for a participant must not be broken by the site or TB Alliance except in the case of a medical emergency, where treatment of a participant is influenced by the knowledge of what dose of sorfequiline or bedaquiline the participant is receiving. The investigator should discuss this with the TB Alliance’s study physician prior to breaking the blind unless knowledge of the treatment regimen is required urgently for a safety concern. TB Alliance’s study physician should be informed of the blind break within 24 h if not discussed prior to unblinding.

## Data collection and management

### Data collection plan {18a}

This study utilized a Direct Data Capturing (DDC) system, which negates the need, in most cases, for a paper source document at the site. Safety lab tests are performed by a central laboratory vendor which involves the shipping of samples to affiliated local labs; results available are sent to the sites via a central e-platform. Sputum samples are analysed by a central laboratory in South Africa, and the results available are sent to sites via the laboratory e-platform. In non-South African sites, local mycobacteriology labs affiliated with the sites are used, and data is entered directly into the DDC system. ECGs taken at the site and associated readings are transmitted to a central ECG system to undergo quality checks, blinded central review, and cardiologist reporting. All data capture and laboratory information systems conform to the Code of Federal Regulations Title 21, Part 11 (21 CFR Part 11) requirements. The data are mapped and transmitted directly from the laboratory information system into the corresponding SDTM datasets.

### Plans to promote participant retention and complete follow‑up {18b}

Each site develops their own ‘recruitment and retention’ plan, specific to their community and location. Participants are reimbursed for their transport, and any other reasonable expense, to and from the site for all trial visits as approved by EC/IRB. The site also provides the participant with a ‘cellular/phone voucher’ to ensure that the participant has the monetary means to contact the site at any time, if they need to.

Site staff contact the participant telephonically between trial visits and just before the next scheduled visit to ask them how they are doing and to remind them of the upcoming visit.

### Data management {19}

A DDC system was designed to collect all the data required by the protocol. Delegated site staff enter data collected per study visit in the DDC system (tablets). Any correction or changes in entry in the DDC are tracked electronically via an audit trail. Safety lab, Myco lab, ECG, and IRT data are all programmatically integrated into the corresponding SDTM datasets.

Adverse events are coded using the Medical Dictionary for Regulatory Activities (MedDRA) terminology. Concomitant medications will be summarized per Anatomical Therapeutic Chemical (ATC) level 2 and level 4 codes. Medications that cannot be assigned a level 2 or level 4 code will be identified in the table as missing the coding level.

The data manager, or their delegate, regularly reviews the DDC data entered by investigator staff for completeness and accuracy.

### Confidentiality {27}

All laboratory specimens, including stored specimens, as well as trial reports, data collection tools, and administrative documents are identified by using only the participant’s unique trial number. All local and central databases are secured with password-protected access systems. The investigators ensure anonymity of the participant, and that all documents are anonymized before being transmitted to TB Alliance.

### Plans for collection, laboratory evaluation and storage of biological specimens for genetic or molecular analysis in this trial/future use {33}

For study participants not randomized to HRZE/HR and who sign a separate informed consent, a blood sample will be drawn at day 1 (or at another time point during the trial if not obtained at day 1) for possible exploratory pharmacogenetic testing. The sample may be used to identify genes that contribute to pharmacokinetic (PK) variability of sorfequiline and bedaquiline, i.e. variability in blood levels of those drugs and their metabolites, such as (but not necessarily restricted to) genes for drug-metabolizing enzymes or drug-transport proteins.

## Statistical methods

### Statistical methods for primary and secondary outcomes {20a}

Demographic and screening/baseline characteristics of the randomized participants will be summarized by treatment arm. There are three analysis populations utilized in this protocol, i.e.:

- *Intent-to-treat (ITT) population* (include all randomized participants who took at least one dose of trial drug)
- *Modified intent-to-treat (MITT) population* (participants who are randomized and take at least one dose of trial drug, without late exclusions, i.e. the lack of MTB culture positive on day 1 and discrepancies between screening rapid test result and corresponding culture/WGS)
- *Per-protocol population* (MITT population excluding participants with major protocol deviations)

The MITT population is the primary population for all efficacy analyses and ITT for all safety analyses.

### Primary objective estimand

- Target population: The analysis population will be mITT adult participants with newly diagnosed, smear-positive, pulmonary DS-TB as defined by the protocol inclusion/exclusion criteria.
- Variable of interest: Time to stable sputum culture conversion to negative status over 8 weeks using data from weekly cultures up to and including week 8.
- Population-level summary: Comparison of time to stable sputum culture to negative status between each TBAJ876 treatment group and 2HRZE/4HR.
- Intercurrent event handling is as follows:
  ◦ Hypothetical strategy: Participants who discontinue the study/lost to follow-up or die due to any cause prior to 8 weeks without having met the criteria for stable sputum culture conversion will be censored at the date of their last visit.

Stable sputum culture conversion to negative status is defined as two negative results (at least 7 days apart without an intervening MTB positive). The primary hypothesis is that for participants randomized to a sorfequiline-containing regimen (at least one sorfequiline regimen), the time to culture negativity by 8 weeks will be superior compared to the participants who are treated with the standard HRZE regimen. Time to stable sputum culture conversion to negative status will also be summarized using the Kaplan–Meier method and displayed graphically. Median event times (and other quartiles) and two-sided 95% CI for each time will be provided.

The key secondary analysis is the proportion of participants with a favourable outcome at 26 weeks after the end of treatment (EOT) based on the MITT population for BPaL vs. HRZE/HR.

The proportion of participants who meet the criteria to stop treatment at week 15 in the three sorfequiline arms will be summarized based on the MITT population.

Relapse rates at 26 weeks after EOT, and separately at 52 weeks after the EOT follow-up period based on the MITT population, will be summarized.

An analysis of the bactericidal activity over 2 weeks, *BA*_TTP_ (1–15), of sorfequiline or bedaquiline in combination with pretomanid and linezolid, relative to HRZE, is determined by the rate of change in TTP over days 1 and 15 of treatment, represented by the model-fitted log(TTP) as calculated by the regression of the observed log(TTP) counts over time. A similar analysis will be performed to assess bactericidal activity over 8 weeks [BA_TTP_ (1–56)].

Change from baseline in measurements of biomarker assays (potentially LAM and other assays), through the course of treatment and the post-treatment follow-up period relative to treatment outcome, will be evaluated.

Key safety analysis will be performed including incidence of adverse events (AEs), study drug modifications, clinical laboratory evaluation, ECGs, and concomitant medications.

For study drugs, plasma concentrations will be summarized, and exposure metrics such as *C*_max_ and AUC will be computed and summarized. Relationships will be explored between exposure metrics and efficacy and safety endpoints.

Trough concentrations of dolutegravir and tenofovir will be summarized for participants living with HIV and will be compared with standard ranges to assess possible interactions with study drugs.

### Interim analysis {21b}

There will be one planned unblinded interim analysis which will contain results by treatment group in aggregate and will include the primary analysis. This will occur after all participants have completed 8 weeks of treatment. The study team and Data Safety Monitoring Committee (DSMC) will have access to the primary end-point analysis.

### Statistics: additional analyses {20b}

A stratified logrank test will be used to compare time to stable sputum culture conversion to negative status between the two regimens with the stratification factors country and severity of disease (AFB 3+ and/or bilateral cavitation). All subgroup analyses will be performed on the ITT, mITT, and PP populations. Additionally, post hoc analyses not originally described in the protocol will be mentioned in the statistical analysis plan (SAP). SAP in supplementary material.

### Methods in analysis to handle protocol non‑adherence and any statistical methods to handle missing data {20c}

Participants with clinical study report (CSR) reportable deviations as evaluated and determined by a review committee prior to database lock will be excluded from the PP population.

### Plans to give access to the full protocol, participant‑level data, and statistical code {31c}

It is the intention that de-identified SDTM datasets including an associated data dictionary will be made available via TB PACTS hosted by CPATH (https://c-path.org/tools-platforms/tb-pacts/). The full protocol and statistical analysis plan will be made available as appendices during the publication of the trial results.

## Oversight and monitoring

### Composition of the coordinating centre and trial steering committee {5d}

The trial is led by the following:

- TB Alliance study physician who leads the core team composed of a biostatistician, medical monitors, a mycobacteriologist, and a nonclinical group
- TB Alliance clinical project manager who leads the clinical operations group consisting of a partner clinical research organization overseeing trial monitoring, vendor management, data management, drug and nondrug supplies, and the finance team

There is also a Data Safety Monitoring Committee (DSMC).

### Composition of the Data Safety Monitoring Committee and its role and reporting structure {21a}

The DSMC is independent of TB Alliance and all project collaborators. It is governed by the DSMC Charter, which describes its purpose and terms of reference. It consists of a chairperson and other seasoned TB disease and trial specialists, a statistician, and a country representative from Tanzania (per Tanzania Medi requirements). The DSMC meeting will be held approximately every 6 months after the first randomized participant. Ad hoc meetings can be called by TB Alliance or the DSMC based on the rates of SAEs, SAEs of particular concern, or any safety concerns that arise during the trial.

The DSMC acts in an advisory capacity to TB Alliance, to safeguard the interest of trial participants by monitoring participant safety, participant risk versus benefit, and general evaluation of the study progress.

See DSMC Charter in supplementary material.

### Adverse events reporting and harms {22}

Adverse events reporting applies to both investigational and control arms in the trial. All AEs and serious adverse events (SAEs) will be collected from the signing of the ICF until the follow-up week 52 visit (end of trial). Treatment-emergent AEs are defined as any AE that occurs after the first dose of IMP and within 28 days after the last dose of IMP. All AEs are recorded in the AE section of the DDC. AEs can be spontaneously reported or elicited during open-ended questioning, examination, or evaluation of a trial participant. The investigator must also promptly review all results of assessments performed as part of the trial, such as laboratory assessment results, ECGs, vital sign monitoring, and physical examinations, and assess them for clinical significance. Each AE is evaluated to determine the severity grade: Grades 1–4 as per the latest version of the DAIDS Severity Grading Scale, its duration (start and end dates or if continuing at the end-of-study visit), its relationship to the study treatment, action taken with respect to study treatment (treatment maintained, dose reduced, permanently discontinued, temporarily discontinued, not applicable), whether medication or therapy was taken/given in relation to the AE, and whether it is a serious adverse event (SAE). All AEs will be followed until satisfactory clinical resolution or stabilization or the end of the follow-up period or early discontinuation of the trial. All SAEs (including updated or significant follow-up information) will be recorded and reported to TB Alliance immediately and within 24 h of awareness.

TB Alliance has a legal responsibility to notify the relevant regulatory authority, IRB/EC, and investigators about the safety of an IMP under clinical investigation.

### Frequency and plans for auditing trial conduct {23}

A risk-based approach is used for quality assurance audits to evaluate if the trial was conducted and the data generated in compliance with the protocol, GCP, and applicable regulatory and ethics committee requirements.

Prior to the conduct of work, independent auditors conduct qualification audits for significant trial vendors to evaluate and confirm the vendor’s capability to perform the planned work in accordance with required standards. During the trial, independent auditors conduct routine requalification audits. The frequency of the audit is based on the risk tier assigned to each vendor, with the highest risk vendors subject to an audit every 2 years, ranging to the lowest risk vendors, which are audited every 10 years.

### Dissemination plan {31a}

Results of this research will be submitted for publication as soon as feasible upon completion of the trial in the form of a joint publication(s) between TB Alliance and investigator(s), including site clinical and laboratory investigators, as appropriate. Publication and authorship will be in accord with the International Association of Journal Editors.

Because the trial is funded, in whole or in part, by the Bill and Melinda Gates Foundation (the ‘Foundation’), all peer-reviewed published research relating to the trial must comply with the Foundation’s Open Access Policy as described from time to time at http://www.gatesfoundation.org/How-We-Work/General-Information/Open-Access-Policy. Specifically, (a) all peer-reviewed published research relating to the trial must be submitted for publication by TB Alliance through the Chronos Open Access Publishing Service established by the Foundation to ensure the immediate and unrestricted access and reuse of all peer-reviewed published research funded, in whole or in part, by the Foundation without any embargo period, and (b) all data underlying the peer-reviewed published research results must be immediately made accessible and open to the public in accordance with the Foundation’s Open Access Policy.

## Discussion

Tuberculosis continues to be one of the most formidable global health threats, especially in the context of rising drug resistance and the setbacks from the COVID-19 pandemic. In response, the TB Alliance initiated protocol NC-009, a phase 2, multi-arm clinical trial that introduces several pioneering features aimed at improving treatment outcomes for drug-sensitive TB (DS-TB). This article explores the innovative aspects of the NC-009 study design, emphasizing its methodological sophistication and translational impact.

At the heart of the NC-009 protocol is its five-arm, partially blinded, randomized trial design. This allows the evaluation of the following:

- Three different doses (25 mg, 50 mg, 100 mg) of the novel diarylquinoline sorfequiline
- Comparison with bedaquiline (200 mg), the current standard among diarylquinolines
- First trial to evaluate BPaL in DS-TB population
- Benchmarking against the HRZE/HR standard of care

The partially blinded methodology balances methodological rigour with operational feasibility.

An important innovation is the adaptive treatment duration based on early treatment response. Participants in the SPaL arms can transition from treatment to follow-up at 15 weeks, provided they meet predefined microbiological and symptomatic recovery criteria. This approach tests the hypothesis that treatment for DS-TB can be safely and effectively shortened for some participants, improving adherence and reducing resource use.

Unlike many trials that focus predominantly on clinical outcomes, NC-009 robustly incorporates pharmacokinetic/pharmacodynamic (PK/PD) modeling, using both sparse and intensive sampling strategies. This allows for the following:

- Correlation of drug exposure with bactericidal activity and adverse events
- Identification of the optimal dose of sorfequiline with a favorable efficacy–safety profile
- Assessment of drug–drug interactions in participants co-infected with HIV

A pragmatic and ethical innovation is the inclusion of a preplanned re-treatment pathway, i.e. participants randomized to the S/BPaL arms who relapse are offered standard retreatment with HRZE/HR, and outcomes are monitored for an additional 26 weeks, as part of the study. This strategy ensures participant safety while enabling long-term outcome assessment and avoids overburdening the local NTP clinics.

The protocol introduces detailed criteria for treatment modification and toxicity monitoring (including visual acuity and neuropathy assessments), critical for regimens containing linezolid. It also includes QTc interval evaluation, providing an opportunity to compare sorfequiline to bedaquiline regarding this safety concern of the latter. These measures ensure participant safety without compromising the study’s scientific goals.

## Trial status

The study is being conducted according to the initial protocol version 1 dated 3 February 2023. No protocol amendments have been generated.

Recruitment started on 24 October 2023 and completed on 30 August 2024, including a total of 309 randomized and eligible participants. The last treatment visit was conducted on 10 March 2025, and the planned ‘last participant last follow-up visit’ is due on 27 February 2026. All ongoing participants are currently in the follow-up phase of the trial.

Three DSMC meetings were held on 5 February 2024, 8 April 2024, and 4 November 2024, and the recommendation was to continue the trial unmodified. The clinical study report (CSR) is planned to be completed by 25 September 2026 following the planned final database lock on 8 May 2026.

## Supplementary Information

Additional file 1. NC-009 Trial Flow Chart_SPIRIT Figure.

Additional file 2. SPIRIT Checklist.

Additional file 3. NC009 Statistical Analysis Plan.

Additional file 4. NC009 Data Safety Monitoring Committee Charter.

## Data Availability

The trial data will be made available after primary publication. All unpublished information/data given to the investigator by TB Alliance shall not be published or disclosed to a third party, other than to the responsible IRB/EC, with the understanding of the confidentiality of their nature, without the prior written consent of TB Alliance.

## Abbreviations

3TC: Lamivudine
AE: Adverse event
AFB: Acid-fast bacilli
ALP: Alkaline phosphatase
ALT: Alanine aminotransferase
ARV: Antiretroviral
AST: Aspartate transaminase
AUC: Area under the curve
BATTP: Bactericidal activity measured by TTP
BPaL: Bedaquiline, pretomanid, and linezolid
CFR: Code of Federal Regulations
Cmax: Maximum plasma concentration
CPK: Creatine phosphokinase
CV: Coefficient of variation
CYP: Cytochrome P450
DAIDS: Divisions of AIDS
DR-TB: Drug-resistant tuberculosis
DSMC: Data Safety Monitoring Committee
DS-TB: Drug-sensitive tuberculosis
DTG: Dolutegravir
EC: Ethics committee
ECG: Electrocardiogram
EOT: End of treatment
GCP: Good clinical practice
HAV: Hepatitis A virus
HbsAg: Hepatitis B surface antigen
HBV: Hepatitis B virus
HCV: Hepatitis C virus
HIV: Human immunodeficiency virus
HRZE: Isoniazid, rifampicin, pyrazinamide, and ethambutol
HR: Isoniazid and rifampicin
IB: Investigator’s Brochure
ICF: Informed consent form
ICH: International Council on Harmonisation
IMP: Investigational medicinal product
IRB: Institutional Review Board
IRT: Interactive response technology
ITT: Intent to treat
IUATLD: International Union Against Tuberculosis and Lung Disease
LAM: Lipoarabinomannan
MAD: Multiple ascending dose
MDR-TB: Multidrug-resistant tuberculosis
MedDRA: Medical Dictionary for Regulatory Activities
MGIT: Mycobacteria growth indicator tube
MIC: Minimum inhibitory concentration
MITT: Modified intent to treat
MTB: *Mycobacterium tuberculosis*
NTP: National TB Program
PCR: Polymerase chain reaction
PD: Pharmacodynamics
PK: Pharmacokinetics
PP: Per protocol
QTcF: QT interval corrected using Fridericia’s formula
SAD: Single ascending dose
SAE: Serious adverse event
SAP: Statistical analysis plan
SD: Standard deviation
SDTM: Study Data Tabulation Model
SmPC: Summary of product characteristics
TB: Tuberculosis
TEAE: Treatment-emergent adverse event
TFV: Tenofovir
TI/NR MDR-TB: Treatment-intolerant/nonresponsive MDR-TB
TPT: TB preventive treatment
TSH: Thyroid-stimulating hormone
TTP: Time to positive
ULN: Upper limit of normal
WGS: Whole genome sequencing
WHO: World Health Organization
XDR-TB: Extensively drug-resistant tuberculosis
