# Supplementary material for: Protocol for a phase 2, partially blinded, randomized trial assessing the safety and efficacy of sorfequiline or bedaquiline in combination with pretomanid and linezolid in adult participants with newly diagnosed, drug-sensitive, smear-positive pulmonary tuberculosis (NC-009)
Source: Trials. 2026 Jan 6;27:102. doi: 10.1186/s13063-025-09413-5 (PMC12869905; doi:10.1186/s13063-025-09413-5)
Supplement: Supplementary file 1 — Additional file 1. NC-009 Trial Flow Chart_SPIRIT Figure. [file 13063_2025_9413_MOESM1_ESM.pdf]

## NC-009 Main Trial Flow Chart

| Period | Screen-ing | Treatment Period 1 | | | | | | | | | | | | Treatment Period 2 | | | | | | Post EOT Follow-up | | | | | | |
|---|---|---|---|---|---|---|---|---|---|---|---|---|---|---|---|---|---|---|---|---|---|---|---|---|---|---|
| **Time of Visit** | Up to 11 days before 1st IMP dose | Day 1 | Day 4 | Day 8 | Day 11 | Day 15 | Week 3 | Week 4 | Week 5 | Week 6 | Week 7 | Week 8 | [1] EOT Make-up Period 1 | Week 9 | Week 12 | [2] Week 15 or EOT | [3] Week 20 | [3] Week 26 EOT | [4] EOT Make-up Period 2 | FU Week 4 | FU Week 8 | FU Week 16 | FU Week 26 | FU Week 38 | FU Week 52 | Early Trial Discontinuation Visit |
| **Visit Window** | -11 to -1 days | | ±1 day | | | | ±3 days | | | | | | | ±5 days | | | | | | ±14 days | | | | | | |
| Informed Consent | X | | | | | | | | | | | | | | | | | | | | | | | | | |
| Demography | X | | | | | | | | | | | | | | | | | | | | | | | | | |
| Medical History | X | | | | | | | | | | | | | | | | | | | | | | | | | |
| Urine Drug Screen | X | | | | | | | | | | | | | | | | | | | | | | | | | |
| Urine Pregnancy Test | X | X | | | | | | X | | X | | X | | | X | | | X | | | | X | | | X | X |
| Urinalysis | X | | | | | | | | | | | | | | | | | | | | | | | | | |
| Exploratory Urine Sample | | X | | | | | | X | | | | X | | | | X | | X | | | | | | | | |
| Inclusion/Exclusion | X | X | | | | | | | | | | | | | | | | | | | | | | | | |
| Randomisation | | X | | | | | | | | | | | | | | | | | | | | | | | | |
| Karnofsky Assessment | X | | | | | | | | | | | | | | | | | | | | | | | | | |
| HIV Screening | X | | | | | | | | | | | | | | | | | | | | | | | | | |
| CD4 Count, Viral Load | X | | | | | | | X | | | | X | | | | | | X | | | | | | | X | X |
| HBsAg, Anti-Hep C Ab | X | | | | | | | | | | | | | | | | | | | | | | | | | |
| Chest X-ray | X | | | | | | | | | | | | | | X | | | X | | | | | X | | X | X |
| Spot Sputum | X | X | X | X | X | X | X | X | X | X | X | X | X | X | X | X | X | X | X | X | X | X | X | X | X | X |
| Visual Acuity | X | | | | | X | | X | | X | | X | | | X | X | X | X | | X | | X | | | | X |
| Vital Signs | X | X | X | X | X | X | X | X | X | X | X | X | X | X | X | X | X | X | X | X | X | X | X | X | X | X |
| Single 12-lead ECG | X | | | | | | | | | | | | | | | | | | | | | | | | | |
| Triple 12-lead ECG | | X | | | | X | | | | | | X | X | | X | | | X | X | | | | | | | X |
| [5] PK Sampling (Trial Drugs) | | X | | X | | X | X | X | X | X | X | X | X | | X | | | X | X | | | | | | | [6] X |
| [7] PK Sampling (ARV) | | X | | X | | | | X | | | | X | | | | | | | | | | | | | | |
| PK Sampling (24 hour) | | | | | | X | | | | | | | | | | | | | | | | | | | | |

| Period | Screen-ing | Treatment Period 1 | | | | | | | | | | | | Treatment Period 2 | | | | | | Post EOT Follow-up | | | | | | Early Trial Discontinuation Visit |
|---|---|---|---|---|---|---|---|---|---|---|---|---|---|---|---|---|---|---|---|---|---|---|---|---|---|---|
| **Time of Visit** | Up to 11 days before 1st IMP dose | Day 1 | Day 4 | Day 8 | Day 11 | Day 15 | Week 3 | Week 4 | Week 5 | Week 6 | Week 7 | Week 8 | [1] EOT Make-up Period 1 | Week 9 | Week 12 | [2] Week 15 or EOT | [3] Week 20 | [3] Week 26 EOT | [4] EOT Make-up Period 2 | FU Week 4 | FU Week 8 | FU Week 16 | FU Week 26 | FU Week 38 | FU Week 52 | |
| **Visit Window** | -11 to -1 days | | ±1 day | | | | ±3 days | | | | | | | ±5 days | | | | | | ±14 days | | | | | | |
| [5]Pharmacogenetic Sample | | X | | | | | | | | | | | | | | | | | | | | | | | | |
| Full Physical Exam | X | X | | | | | | | | | | | | | | X | | X | X | | | | | | | |
| Height | X | | | | | | | | | | | | | | | | | | | | | | | | | |
| Cardiovascular Exam | | | X | X | X | X | X | X | X | X | X | X | X | X | X | | X | | | X | X | X | | | | X |
| Symptom Directed Exam | | | X | X | X | X | X | X | X | X | X | X | X | X | X | | X | | | X | X | X | X | X | X | X |
| TB Symptoms Profile | | X | | | | | | X | | | | X | X | | | X | | X | X | | | | X | | X | X |
| Brief Peripheral Neuropathy Assessment | X | | | | | X | | X | | X | | X | X | | X | X | X | X | X | | | X | | | | X |
| Laboratory Safety Test | X | X | | X | | X | X | X | X | X | X | X | X | X | X | X | X | X | X | | | | | | | X |
| Exploratory Blood Sample | | X | | | | | | X | | | | X | | | | X | | X | | | | | | | | |
| IMP Administration, Adherence | | X | X | X | X | X | X | X | X | X | X | X | X | X | X | X | X | X | X | | | | | | | [5]X |
| Quality of Life Questionnaire | | X | | | | | | | | | | | | | | X | | | | | | | | | X | X |
| Concomitant Medications | | X | X | X | X | X | X | X | X | X | X | X | X | X | X | X | X | X | X | X | X | X | X | X | X | X |
| Adverse Events | X | X | X | X | X | X | X | X | X | X | X | X | X | X | X | X | X | X | X | X | X | X | X | X | X | X |

1. The EOT Make-up Period 1 visit will only be performed if the participant has missed 4 to 14 cumulative IMP doses. If less than 4 doses were missed, the participant is also required to make up those missed doses by the Week 8 visit, but there is no need to perform an additional visit prior to the transition to Treatment Period 2.
2. Week 15 can either be the EOT visit for participants who complete treatment at this visit in the TBAJ876-Pa-L arms or a regular visit for participants who continue treatment until Week 26.
3. Visit only applicable to participants who complete treatment at Week 26.

4. The EOT Make-up Period 2 visit will only be performed if the participant has missed 6 to 28 cumulative IMP doses (during Treatment Period 2). Making up missed doses is required for participants who complete treatment at Week 15 or 26; however, the additional EOT visit is not required prior to proceeding to the Follow-up Period if less than 6 doses were missed.
5. Excluding participants randomised to 2HRZE/4HR.
6. IMP adherence will be assessed and PK samples will be taken only if the Early Trial Discontinuation Visit is done during either of the treatment periods and not during follow up.
7. Applies to all treatment arms; only for participants living with HIV and on ARV.

**Re-treatment Flow Chart**

| **Period** | **Re-treatment** | | | | | | **Follow-up** | | |
|---|---|---|---|---|---|---|---|---|---|
| **Time of Visit** | [A]**Day 1** | **Week 2** | **Week 4** | **Week 8** | **Week 16** | **Week 26** | **Week 8 after EOT** | **Week 16 after EOT** | **Week 26 after EOT** |
| **Visit Window** | **±5 days** | | | | | | **±14 days** | | |
| [B]Chest X-ray | X | | | | | X | | | X |
| [C]Spot Sputum | X | X | X | X | X | X | X | X | X |
| [C]Visual Acuity | X | | | | | X | | | X |
| Vital Signs | X | X | X | X | X | X | X | X | X |
| Height | X | | | X | | | | | |
| Symptom Directed Exam | X | X | X | X | X | X | X | X | X |
| [C]Laboratory Safety Test | X | X | X | X | X | X | | | |
| IMP Administration, Adherence | X | X | X | X | X | X | | | |
| Concomitant Medications | X | X | X | X | X | X | X | X | X |
| Adverse Events | X | X | X | X | X | X | X | X | X |

A: Day 1 is the day of the first dose of HRZE in participants who are retreated
B: Chest X-ray does not need to be repeated if one was performed within 4 weeks prior to Day 1.
C: Procedure does not need to be repeated if performed within 2 weeks prior to Day 1.
