# Supplementary material for: Protocol for a phase 2, partially blinded, randomized trial assessing the safety and efficacy of sorfequiline or bedaquiline in combination with pretomanid and linezolid in adult participants with newly diagnosed, drug-sensitive, smear-positive pulmonary tuberculosis (NC-009)
Source: Trials. 2026 Jan 6;27:102. doi: 10.1186/s13063-025-09413-5 (PMC12869905; doi:10.1186/s13063-025-09413-5)
Supplement: Supplementary file 2 — Additional file 2. SPIRIT Checklist. [file 13063_2025_9413_MOESM2_ESM.docx]

# Reporting checklist for protocol of a clinical trial.

Based on the SPIRIT guidelines.

## Instructions to authors

Complete this checklist by entering the page numbers from your manuscript where readers will find each of the items listed below.

Your article may not currently address all the items on the checklist. Please modify your text to include the missing information. If you are certain that an item does not apply, please write "n/a" and provide a short explanation.

Upload your completed checklist as an extra file when you submit to a journal.

In your methods section, say that you used the SPIRIT reporting guidelines, and cite them as:

Chan A-W, Tetzlaff JM, Gøtzsche PC, Altman DG, Mann H, Berlin J, Dickersin K, Hróbjartsson A, Schulz KF, Parulekar WR, Krleža-Jerić K, Laupacis A, Moher D. SPIRIT 2013 Explanation and Elaboration: Guidance for protocols of clinical trials. BMJ. 2013;346:e7586

|  |  | Reporting Item | Page Number |
| --- | --- | --- | --- |
| **Administrative information** |  |  |  |
| Title | [#1](https://www.goodreports.org/reporting-checklists/spirit/info/#1) | Protocol for a phase 2, partially-blinded, randomised trial assessing the safety and efficacy of sorfequiline or bedaquiline, in combination with pretomanid and linezolid in adult participants with newly diagnosed, drug-sensitive, smear-positive pulmonary tuberculosis (NC009). | 1 |
| Trial registration | [#2a](https://www.goodreports.org/reporting-checklists/spirit/info/#2a) | ClinicalTrials.gov: NCT 06058299 (URL: https://clinicaltrials.gov/study/NCT06058299?cond=tuberculosis&term=NC-009&rank=1) | 1 |
| Trial registration: data set | [#2b](https://www.goodreports.org/reporting-checklists/spirit/info/#2b) | All data included in primary ClinicalTrials.gov registry: NCT 06058299 | 1 |
| Protocol version | [#3](https://www.goodreports.org/reporting-checklists/spirit/info/#3) | 03 Feb 2023 Version 1.0 | 1 |
| Funding | [#4](https://www.goodreports.org/reporting-checklists/spirit/info/#4) | This work was supported by TB Alliance (Global Alliance for TB Drug Development) with funding from Australia’s Department of Foreign Affairs and Trade, the Gates Foundation [OPP1129600], the Foreign, Commonwealth and Development Office (United Kingdom), Germany’s Federal Ministry of Education and Research through KfW, Irish Aid, and the United States Agency for International Development. | 15 |
| Roles and responsibilities: contributorship | [#5a](https://www.goodreports.org/reporting-checklists/spirit/info/#5a)  #31b | Olugbosi M ^1^, Beumont M ^2^, Lombard L ^1^, Nedelman J ^2^, Timm J ^2^, Black T ^2^, Barry R ^3^, Hickman D ^2^, Lombardi A ^2^, Betteridge M ^2^, Egizi E ^2^, Marcopulos L ^2^, Henderson J ^1^, Seidel S ^2^, Foraida S ^2^, Benhayoun M ^2^, Sun E ^2^  ^1^ TB Alliance, South Africa  ^2^ TB Alliance, USA  ^3^ RTI International, USA  OM, BM, LL, NJ, TJ, BT, BR, HD, LA, BeM, EE, ML, HJ, SS, FS, BnM, SE initiated the study design and implemented the study. OM is study physician, and LL is clinical project manager. NJ is pharmacokinetics, BT is biologist, TJ is microbiologist, BeM is clinical data manager, EE is back up clinical project manager & medical writer, ML is drug supply manager, HJ oversees quality assurance, SS is community engagement manager. All authors contributed to refinement of the study protocol and approved the final manuscript. | 1;15 |
| Roles and responsibilities: sponsor contact information | [#5b](https://www.goodreports.org/reporting-checklists/spirit/info/#5b) | Dr Morounfolu Olugbosi  TB Alliance  80 Pine Street,  20th Floor,  New York, NY 10005 `  Phone +1-212-227-7540  [morounfolu.olugbosi@tballiance.org](mailto:morounfolu.olugbosi@tballiance.org) | 1 |
| Roles and responsibilities: sponsor and funder | [#5c](https://www.goodreports.org/reporting-checklists/spirit/info/#5c) | TB Alliance has the ultimate authority for trial design; collection, management, analysis, and interpretation of data; writing the final report and the decision to submit the report for publication. Funders have no input in the listed activities. |  |
| Roles and responsibilities: committees | [#5d](https://www.goodreports.org/reporting-checklists/spirit/info/#5d) | The trial is led by:   - TB Alliance Study Physician who leads the core team composed of biostatistician, medical monitors, mycobacteriologist, and non-clinical group. - TB Alliance Clinical Project Manager who leads the clinical operations group consisting of a partner Clinical Research Organization overseeing trial monitoring, vendor management, data management, drug and non-drug supplies, and the finance team.   There is also a Data Safety Monitoring Committee (DSMC). | 11-12 |
| **Introduction** |  |  |  |
| Background and rationale | [#6a](https://www.goodreports.org/reporting-checklists/spirit/info/#6a) | Tuberculosis (TB) is a preventable and usually curable disease. Yet in 2023, TB returned to being the world’s leading cause of death from a single infectious agent, following 3 years in which it was replaced by coronavirus disease (COVID-19) (1).  Existing drug sensitive TB (DS-TB) treatment regimens are efficacious but lengthy in duration and involve multidrug therapy combinations i.e., an intensive phase of 2 months of isoniazid (H), rifampicin (R), pyrazinamide (Z), and ethambutol (E) followed by a continuation phase of 4 months of isoniazid and rifampicin (HRZE/HR); this treatment regimen was first introduced as the standard of care more than 40 years ago (2). Long and complex treatments lead to high rates of non-adherence, which often result in unfavourable outcomes, emergence of drug resistance, continued spread of disease, and increased mortality. Drug-drug interactions caused by rifampicin’s potent induction of CYP enzymes are another major challenge with the HRZE/HR regimen, particularly for women on hormonal contraception and TB participants co-infected with HIV taking anti-retroviral therapies (ARVs). While a 4-month rifapentine-based regimen for treatment of pulmonary DS-TB is now recommended by the WHO as a possible alternative to HRZE/HR but with very limited global roll-out (3), there is a need for new TB drugs and drug regimens that are efficacious, safe, well-tolerated, simpler, and that can further shorten the overall treatment duration, thereby improving adherence and patient outcomes.  The combination of bedaquiline, a first generation diarylquinoline, with pretomanid (a nitroimidazole), and linezolid (an oxazolidinone) administered for 26 weeks has been extensively studied and is referred to as the BPaL regimen. The BPaL regimen demonstrated robust efficacy (>90% relapse-free cure) in adults with pulmonary XDR-TB and TI/NR MDR-TB (pre-WHO 2021 definitions) when linezolid was dosed at 1200 mg daily (4). When the linezolid dose was decreased to 600 mg daily, efficacy was retained, and the regimen demonstrated improved tolerability (5). BPaL (plus moxifloxacin in patients with fluoroquinolone-susceptible TB) for 6 months is currently recommended for patients with drug resistant TB (DR-TB) sensitive to all components of the regimen (6). The high potency of the BPaL regimen has potential to also shorten treatment for DS-TB (7).  Sorfequiline (S) is a second generation diarylquinoline selected for development based on initial positive nonclinical results. In vitro and in vivo studies have shown the increased potency of sorfequiline over bedaquiline. The sorfequiline minimum inhibitory concentration (MIC) against a phylogenetically diverse panel of 96 clinical strains was approximately 10-fold lower than the corresponding bedaquiline MICs and in the acute and chronic mouse models of TB, sorfequiline was superior to bedaquiline when administered as monotherapy or in combination with PaL. Crucially, the activity of sorfequiline against the most common type of bedaquiline resistant mutant (Rv0678 mutant) was similar to that of bedaquiline against a wild type strain, both in vitro and in the mouse model(8) . Based on in vitro cardiac potassium channel current inhibition screening studies (hERG assay) and in vivo cardiovascular safety assessments, sorfequiline has shown a reduced risk for QTc prolongation compared with bedaquiline(9) . Sorfequiline thus has the potential to contribute both to increased efficacy and improved safety, and to a shorter TB treatment regimen for both DS-TB and DR-TB. | 2-3 |
| Background and rationale: choice of comparators | [#6b](https://www.goodreports.org/reporting-checklists/spirit/info/#6b) | The 6-month treatment regimen is composed of four first-line TB medicines – HRZE/HR per weight band. This regimen is well known and has been widely adopted worldwide for decades; while using it, about 85% of participants will have a successful treatment outcome (1). This regimen is based on seminal TB treatment studies conducted by the British Medical Research Council in the second half of the 20th century (10). | 6 |
| Objectives | [#7](https://www.goodreports.org/reporting-checklists/spirit/info/#7) | List of primary, key secondary, secondary and exploratory objectives and associated end-points in Table 1. | 3 |
| Trial design | [#8](https://www.goodreports.org/reporting-checklists/spirit/info/#8) | This is a phase 2, multi-center, partially blinded, randomised clinical trial where at least 300 participants with DS‑TB who meet all the inclusion criteria and none of the exclusion criteria, aged 18 to 65, will be randomised to receive 1 of the 5 active treatment regimens (at least 60 participants per regimen). Participants will be randomised in equally, using an interactive response technology (IRT) that stratifies based on country and severity of disease (AFB 3+ and/or bilateral cavitation) to 1 of the 5 daily treatment regimens.  The trial consists of the following periods:   - *Screening Period*: Screening visit up to 11 days prior to randomisation (Day 1) - *Treatment Period 1 (TP1):* Day 1 through Week 8 (SPaL or BPaL or HRZE) - *Treatment Period 2 (TP2):*    - Week 9 through Week 15 (participants in the SPaL arms that meet criteria for early completion of treatment).   - Week 9 through Week 26 (participants in the HRZE/HR or BPaL arms, and participants in the SPaL arms who do not meet criteria for early completion of treatment). - *Post end of treatment (EOT) Follow-up Period*: 52 weeks after EOT   After receiving 8 weeks of treatment, participants in the SPaL arms and in the control arm (HRZE/HR) will continue treatment with HR, and participants randomised to BPaL will continue treatment with BPaL. Treatment completion will be allowed at week 15 in participants randomised to the SPaL arms, if the criteria below are met*:*   - Week 8 sputum Mycobacterial Growth Indicator Tube (MGIT) culture is negative, and - The participant has no TB-related symptoms by Week 15.   If the MGIT result is MTB positive and/or there are still TB symptom(s), participants will continue to receive HR (in the SPaL arms) and will complete a total of 26 weeks of treatment.  Note: The inability to produce sputum will be considered as a negative MGIT culture result and therefore can be used to determine the participants’ eligibility to complete treatment at Week 15.  Re-Treatment  Participants randomised to the 3 SPaL arms and BPaL arm who relapse or experience treatment failure (see definition of relapse and treatment failure in Table 2) will be re-treated with HRZE/HR, provided that drug susceptibility data do not reveal emergence of resistance or the participant has a contraindication for receiving HRZE/HR, in which case they will be referred to the local national TB programme (NTP) for further management. Participants who are re-treated will be followed for 26 weeks after the end of their re-treatment.  In the case of treatment failure or relapse in participants randomised to the control arm (HRZE/HR), the participant will be referred to the local NTP for further management, and the treating physicians will be provided with the DST results and medical report from the investigator, should an individualized regimen be indicated.  For all participants except those on HRZE/HR, blood samples to assess the pharmacokinetics (PK) of study drugs are collected weekly pre-dose and at 1, 3, and 5 hours post-dose at Day 15 and Week 8. Participants at some sites may volunteer for 24-hour intensive sampling at Day 15. In addition, pre-dose samples are collected at Days 1 and 8 and Weeks 4 and 8 to assess the PK of tenofovir and dolutegravir.  See Figure 1 for NC-009 Trial Design. | 3-4 |
| **Methods: Participants, interventions, and outcomes** |  |  |  |
| Study setting | [#9](https://www.goodreports.org/reporting-checklists/spirit/info/#9) | The trial will be performed at multiple centres globally including Georgia, the Philippines, South Africa, Tanzania, and Uganda. These are a total of 22 clinical trial units recruiting participants from TB clinics in catchment areas. See Table 3 for list of countries, cities and site names. | 5 |
| Eligibility criteria | [#10](https://www.goodreports.org/reporting-checklists/spirit/info/#10) | **Inclusion Criteria**   1. Participants are required to meet all the following inclusion criteria during the screening period to be randomised. 2. Signed written informed consent prior to undertaking any trial-related procedures. 3. Participants aged 18 to 65 years, inclusive. 4. Body weight (in light clothing and no shoes) ≥35 kg. 5. Sputum positive for tubercle bacilli (at least 1+ on the IUATLD/WHO scale on smear microscopy) at the trial laboratory. 6. DS-TB participants defined as the following:    1. Sensitive to rifampicin and isoniazid by rapid sputum-based test AND    2. Either newly diagnosed for TB or have a history of being untreated for at least 3 years after cure from a previous episode of TB. 7. A chest X-ray during the screening period or within 14 days of screening which in the opinion of the investigator is compatible with pulmonary TB. 8. Be of non-childbearing potential OR using effective methods of birth control as defined below:    1. Non-childbearing Potential    2. Participant is not heterosexually active or practices sexual abstinence OR    3. Female participant or male participant’s female sexual partner: bilateral oophorectomy, bilateral tubal ligation, and/or hysterectomy or has been postmenopausal with a history of no menses for at least 12 consecutive months OR    4. Male participant or female participant’s male sexual partner: vasectomized or has had a bilateral orchidectomy at least 3 months prior to screening.    5. Effective method of birth control is defined as one of the following:    6. Double-barrier method, which can include a combination of male condom, diaphragm, cervical cap, or female condom.    7. Note: Female and male condom should not be used together.    8. Combination of a barrier method with hormone-based contraceptives or an intra‑uterine device.    9. Both male and female participants must be willing to continue practicing birth control methods and not be planning to conceive throughout treatment and for 6 months after the last dose of IMP.     **Exclusion Criteria**   1. Participants will be excluded from participating in the trial if they meet any of the following criteria during the screening period: 2. History or presence of pulmonary, hepatic, musculoskeletal abnormalities, renal, hematological, gastrointestinal, endocrine, immunologic, dermatologic, neurological, psychiatric disease, or any condition as determined by the investigator that could impact the participant’s ability to participate in the trial. 3. Cardiovascular abnormalities, such as pathological heart murmur, acute or chronic cardiac diseases including but not limited to angina (stable or unstable), acute coronary syndrome in the last 6 months, and any type of cardiomyopathy. 4. Karnofsky performance status score of <60%. 5. Abuse of alcohol or illegal drugs that in the opinion of the investigator would compromise the participant’s safety or ability to follow through with all protocol‑specified restrictions, visits, and evaluations. 6. Historical and/or current use of local traditional medications/herbs (such as St. John’s wort) which in the opinion of the investigator would compromise the participant’s safety or ability to follow through with all protocol-specified restrictions, visits, and evaluations. 7. Being, or about to be, treated for malaria. 8. Is critically ill and, in the judgment of the investigator, has a diagnosis likely to result in death during the trial or the follow-up period. 9. Any evidence of extrapulmonary TB. Pleural effusion occupying <50% of hemithorax or concomitant intra- or extra-thoracic lymphadenopathy are not exclusions. 10. For participants living with HIV only:     1. CD4+ count <200 cells/μL.     2. WHO Clinical Stage 4 HIV disease.     3. Participants who do not agree to use DTG/TFV/3TC if ARV therapy is indicated, whilst participating in the trial and are randomised to the sorfequiline or the BPaL regimen (participants randomised to the HRZE/HR regimen can use any ARV compatible with the regimen).     4. If initiation of ARV therapy is indicated within Treatment Period 1, participants who are known to be intolerant, non-responsive to DTG/TFV/3TC or have DTG/TFV/3TC as a contraindication. 11. Having participated in other clinical trials with investigational agents within 8 weeks prior to Day 1 or currently randomised in an investigational drug trial. 12. Participants with QTcF interval >450 msec at screening based on ECG measurement. 13. Participants with any of the following at the screening visit per medical history:     1. A personal or family history of congenital QT prolongation.     2. A history of known, untreated, and uncontrolled hypothyroidism.     3. A history of or ongoing bradyarrhythmia.     4. A history of Torsade de Pointe. 14. Unstable Diabetes Mellitus which required hospitalization for hyper- or hypo-glycemia within the past year prior to the start of screening. 15. Females who have a positive pregnancy test during the screening visit or are already known to be pregnant, breastfeeding, or planning to conceive a child during the trial or within 6 months of completing treatment with IMP. Males planning to conceive a child during the trial or within 6 months of stopping treatment with IMP. 16. Any diseases or conditions in which the use of standard TB drugs or any of their components is contraindicated, including but not limited to drug allergy. 17. Use of any drug within 30 days prior to randomisation known to prolong QTc interval 18. Participants with the following toxicities at screening:     1. Platelets <100,000/mm^3^     2. Creatinine >1.3 x ULN     3. Haemoglobin <9.5 g/dL or <95 g/L     4. Absolute neutrophil count <800/mm^3^     5. Serum potassium less than the lower limit of normal for the laboratory.     6. ALT and/or AST ≥2.5 x ULN     7. Total bilirubin ≥1.6 x ULN     8. Direct bilirubin >1 x ULN     9. Haemoglobin A1c ≥8.0%     10. Total lipase ≥1.5 x ULN     11. Total amylase ≥1.5 x ULN     12. CPK >3 x ULN (if >3 x ULN, enquire about the participant’s recent strenuous activity and consider repeating the test within the screening window)     13. TSH >1 x ULN     14. Positive results at screening for HBsAg, HAV IgM, or hepatitis C antibodies. Participants with positive hepatitis C antibodies but negative PCR can be allowed in the trial. | 5-6 |
| Interventions: description | [#11a](https://www.goodreports.org/reporting-checklists/spirit/info/#11a) | Treatment will be administered by the site to the participant at scheduled trial visits. In between scheduled site visits, the participants will be responsible for the administration of their own investigation medicinal product (IMP).  *1^st^ 3 treatment arms*: 25 mg/ 50 mg/ 100 mg of sorfequiline + 200 mg pretomanid + 600 mg linezolid for 8 weeks (TP1) followed by 7 weeks or 18 weeks of HR based on participant meeting criteria to stop treatment at week 15 (TP2).  *4^th^ treatment arm*: 200 mg bedaquiline + 200mg pretomanid + 600 mg linezolid for 1st 8 weeks (TP1) followed by 100 mg bedaquiline + 200mg pretomanid + 600 mg linezolid for 18 weeks (TP2).  *5^th^ treatment arm*: HRZE for 8 weeks followed by HR for 18 weeks. | 3 |
| Interventions: modifications | [#11b](https://www.goodreports.org/reporting-checklists/spirit/info/#11b) | At no time should the participant be treated with a single agent. If any of the components other than linezolid need to be interrupted, the entire regimen must be interrupted. Dose adjustments are not allowed for sorfequiline, bedaquiline, pretomanid, or HRZE/HR. Linezolid can be reduced from 600 mg to 300mg or temporarily interrupted or permanently discontinued. No minimum number of doses of linezolid is specified in the protocol.  During TP1 and 2, the full regimen can be interrupted for up to 14 and 28 cumulative doses for drug related toxicities respectively. | 6-7 |
| Interventions: adherance | [#11c](https://www.goodreports.org/reporting-checklists/spirit/info/#11c) | Participants are reminded at each dispensing visit about the importance of adherence and compliance to the IMP. Site staff encourage participants to contact them between visits if they have any questions about their medications or if they are feeling unwell. The site contact details (24 hours) are provided to the participant at the screening visit via a ‘Participant Contact Card’. | 7 |
| Interventions: concomitant care | [#11d](https://www.goodreports.org/reporting-checklists/spirit/info/#11d) | All therapies (prescriptions or over-the-counter medications, including vitamins and herbal supplements) different from the trial drugs are recorded in the concomitant therapy section of the Direct Data Capturing (DDC) platform.  *Prohibited Concomitant Medications*:  The following therapies are not allowed during the trial: All medicinal products used to treat pulmonary TB; isoniazid prophylaxis as treatment preventive therapy (TPT) preventative for participants living with HIV; Monoamine Oxidase Inhibitors (due to linezolid, applicable to SPaL and BPaL regimens).  *Concomitant Medication to be avoided*:  The following concomitant medications should be avoided during and for 14 days after treatment with IMP to prevent possible drug interactions with the IMP: any drug known to be hepatotoxic (applicable for all treatment regimens) e.g. NSAIDS, acetaminophens; any drug known to prolong QTc interval (applicable to SPaL and BPaL regimens) e.g. chloroquine, amiodarone; any drug known to induce significant myelosuppression (due to linezolid, applicable to SPaL and BPaL regimens) e.g. chloramphenicol; systemic use of strong CYP3A4 inhibitors, for more than 14 consecutive days (applicable to SPaL and BPaL regimens) e.g. azole antifungals; systemic use of strong and moderate CYP3A4 inducers should be avoided 14 days before and during treatment (applicable to SPaL and BPaL regimens) e.g. phenytoin, carbamazepine serotonergic antidepressants (SPaL and BPaL regimens) e.g. fluoxetine, paroxetine; strong P-gp inhibitors for more than 3 consecutive days (applicable to SPaL and BPaL regimens) e.g. cyclosporine. | 7 |
| Outcomes | [#12](https://www.goodreports.org/reporting-checklists/spirit/info/#12) | See table 1 for study objective and relevant outcomes/end points. See table 2 for Study Outcome Definitions. | 7 |
| Participant timeline | [#13](https://www.goodreports.org/reporting-checklists/spirit/info/#13) | See Trial Flow Chart in NC-009 protocol (supplementary materials). | 7 |
| Sample size | [#14](https://www.goodreports.org/reporting-checklists/spirit/info/#14) | Sample size for this trial will be at least 60 participants per treatment regimen. Sample size assumptions below are selected to be conservative to achieve adequate power given expected minimal dropout prior to Week 8.  Based on data from previous trials of DS-TB, it is assumed that the probability of participants achieving stable sputum culture conversion to negative status by Week 8 to TP1 in the control regimen (HRZE/HR) is 0.50 (11, 12). The number of events and power required is based on comparison of the primary endpoint i.e. time to stable sputum culture conversion to negative status using data from weekly cultures through Week 8 to TP1.  The trial will require approximately 76 events (stable sputum culture conversion to negative status) among approximately 120 randomised participants. This number ensures that a 2-sided, α = 0.05 logrank test procedure will have 80% power when the true hazard ratio of sorfequiline vs. HRZE is 2.0 and 90% power when the hazard ratio is 2.2. It is assumed that calculations are sufficiently conservative to ensure that the required number of events will be observed by the time the analysis is conducted using Week 8 data. To minimize Type I error, the comparisons for inference will be ordered starting with the highest sorfequiline dose regimen. | 7-8 |
| Recruitment | [#15](https://www.goodreports.org/reporting-checklists/spirit/info/#15) | Participating sites foster and maintain positive working relationships with the local TB clinics in their areas via the Community Engagement (CE) teams and activities. Before a trial commences, the site CE team meets with and informs community stakeholders e.g. Community Advisory Board (CAB), TB clinic nurses and doctors about the trial. When the trial starts, the TB clinics identify patients who have been newly diagnosed with DS-TB, and they inform the site CE contact person. The CE contact person talks with the patient to find out if they would like to know more about the trial, and if they agree, they invite them to the site. The CE team arranges transportation for the participant from their home or TB clinic to the site while they still test positive for TB. This process facilitates a good relationship between CAB, TB clinics and trial sites, and as a consequence hopefully good recruitment to the trial. | 8 |
| **Methods: Assignment of interventions (for controlled trials)** |  |  |  |
| Allocation: sequence generation | [#16a](https://www.goodreports.org/reporting-checklists/spirit/info/#16a) | Participants were randomised to one of the 5 regimens in a equal ratio, using an IRT system with directions provided to each site via the IRT user manual. |  |
| Allocation concealment mechanism | [#16b](https://www.goodreports.org/reporting-checklists/spirit/info/#16b) | Per participant allocation of IMP is via the IRT system randomly randomizes participants to a treatment arm considering the stratification factors and generates the kit numbers per visit for the participant. |  |
| Allocation: implementation | [#16c](https://www.goodreports.org/reporting-checklists/spirit/info/#16c) | IRT system randomly allocates participants to a treatment arm considering the stratification factors and generates the kit numbers per visit for the participant. Participant enrolment is performed by site staff via the Direct Data Collection (DDC) platform. |  |
| Blinding (masking) | [#17a](https://www.goodreports.org/reporting-checklists/spirit/info/#17a) | The trial is partially blinded i.e. sorfequiline and bedaquiline will be blinded during the first 8 weeks of trial treatment; participants randomised to the sorfequiline or bedaquiline arms will receive open label pretomanid and linezolid. After the Week 8 visit, participants will be unblinded if they are randomised to sorfequiline or bedaquiline but the dose of sorfequiline remains blinded throughout the study. Participants randomised to the HRZE/HR arm will receive open label IMP. Only the unblinded statistician will have access to unblinded data. | 8 |
| Blinding (masking): emergency unblinding | [#17b](https://www.goodreports.org/reporting-checklists/spirit/info/#17b) | The blind for a participant must not be broken by the site or TB Alliance except in the case of a medical emergency, where treatment of a participant is influenced by the knowledge of what dose of sorfequiline or bedaquiline the participant is receiving. The investigator should discuss this with the TB Alliance’s Study Physician prior to breaking the blind unless knowledge of the treatment regimen is required urgently for a safety concern. TB Alliance’s Study Physician should be informed of the blind break within 24 hours if not discussed prior to unblinding. | 8 |
| **Methods: Data collection, management, and analysis** |  |  |  |
| Data collection plan | [#18a](https://www.goodreports.org/reporting-checklists/spirit/info/#18a) | This study utilized a Direct Data Capturing (DDC) system, which negates the needs, in most cases, for a paper source document at the site. Safety lab tests are performed by a central laboratory vendor which involves shipping of samples to affiliated local labs; results available are sent to the sites via a central e-platform. Sputum samples are analysed by a central laboratory in South Africa, and the results available are sent to sites via the laboratory e-platform. In non-South African sites, local mycobacteriology labs affiliated with the sites are used and data is entered directly into the DDC system. ECGs taken at the site and associated readings are transmitted to a central ECG system to undergo quality checks, blinded central review and cardiologist reporting. All data capture and laboratory information systems conform to the Code of Federal Regulations Title 21, Part 11 (21 CFR Part 11) requirements. The data are mapped and transmitted directly from the laboratory information system into the corresponding SDTM datasets. | 8-9 |
| Data collection plan: retention | [#18b](https://www.goodreports.org/reporting-checklists/spirit/info/#18b) | Each site develops their own ‘Recruitment and Retention’ plan, specific to their community and location. Participants are reimbursed for their transport, and any other reasonable expense, to and from the site for all trial visits as approved by EC/IRB. The site also provides the participant with a ‘cellular / phone voucher’ to ensure that the participant has the monetary means to contact the site at any time, if they need to.  Site staff contact the participant telephonically between trial visits and just before the next scheduled visit to ask them how they are doing and to remind them of the upcoming visit. | 9 |
| Data management | [#19](https://www.goodreports.org/reporting-checklists/spirit/info/#19) | A DDC system was designed to collect all the data required by the protocol. Delegated site staff enter data collected per study visit in the DDC system (tablets). Any correction or changes in entry in the DDC are tracked electronically via an audit trail. Safety lab, Myco lab, ECG & IRT data are all programmatically integrated into the corresponding SDTM datasets.  Adverse events are coded using the Medical dictionary for regulatory activities (MedDRA) terminology. Concomitant medications will be summarised per Anatomical Therapeutic Chemical (ATC) level 2 and level 4 code. Medications that cannot be assigned a level 2 or level 4 code will be identified in the table as missing the coding level.  The Data Manager, or their delegate, regularly reviews the DDC data entered by investigator staff for completeness and accuracy. | 9 |
| Statistics: outcomes | [#20a](https://www.goodreports.org/reporting-checklists/spirit/info/#20a) | Demographic and screening/baseline characteristics of the randomised participants will be summarised by treatment arm. There are 3 analysis population utilized in this protocol i.e.:   - *Intent-to-treat (ITT) Population* (include all randomised participants who took at least 1 dose of trial drug) - *Modified Intent-to-treat (MITT) Population* (participants who are randomised and take at least 1 dose of trial drug, without late exclusions i.e. the lack of MTB culture positive on day 1 and discrepancies between screening rapid test result and corresponding culture/WGS) - *Per Protocol Population* (MITT population excluding participants with major protocol deviations)   The MITT population is the primary population for all efficacy analyses and ITT for all safety analysis.  Primary Objective Estimand   - Target population: The analysis population will be mITT adult participants with newly diagnosed, smear-positive, pulmonary DS-TB as defined by the protocol inclusion/exclusion criteria. - Variable of interest: time to stable sputum culture conversion to negative status over 8 weeks using data from weekly cultures up to and including Week 8. - Population-level summary: comparison of time to stable sputum culture to negative status between each TBAJ876 treatment group and 2HRZE/4HR. - Intercurrent event handling:   - Hypothetical strategy: Participants who discontinue the study/lost to follow-up or die due to any cause prior to 8 weeks without having met the criteria for stable sputum culture conversion will be censored at the date of their last visit.   The primary hypothesis is that for participants randomised to a sorfequiline-containing regimen (at least 1 sorfequiline regimen), the time to culture negativity by 8 weeks will be superior, compared to the participants who are treated with the standard HRZE regimen. Time to stable sputum culture conversion to negative status will also be summarized using the Kaplan-Meier method and displayed graphically. Median event times (and other quartiles) and 2-sided 95% CI for each time will be provided.  The key secondary analysis is the proportion of participants with a favourable outcome at 26 weeks after end of treatment (EOT) based on the MITT population for BPaL vs. HRZE/HR.  The proportion of participants who meet the criteria to stop treatment at Week 15 in the 3 sorfequiline arms will be summarized based on the MITT population.  Relapse rates at 26 weeks after EOT, and separately at 52 weeks after the EOT follow-up period based on the MITT population will be summarized.  An analysis of the bactericidal activity over 2 weeks, BA_TTP_(1-15), of sorfequiline or bedaquiline in combination with pretomanid and linezolid, relative to HRZE, as determined by the rate of change in TTP over Days 1 to 15 of treatment, represented by the model-fitted log(TTP) as calculated by the regression of the observed log(TTP) counts over time. A similar analysis will be performed to assess bactericidal activity over 8 weeks [BA_TTP_(1-56)].  Change from baseline in measurements of biomarker assays (potentially LAM and other assays), through the course of treatment and the post-treatment follow-up period relative to treatment outcome will be evaluated.  Key safety analysis will be performed including incidence of adverse events (AE), study drug modifications, clinical laboratory evaluation, ECGs, concomitant medications etc.  For study drugs, plasma concentrations will be summarized, and exposure metrics such as C_max_ and AUC will be computed and summarized. Relationships will be explored between exposure metrics and efficacy and safety endpoints.  Trough concentrations of dolutegravir and tenofovir will be summarized for participants living with HIV and will be compared with standard ranges to assess possible interactions with study drugs. | 10-11 |
| Statistics: additional analyses | [#20b](https://www.goodreports.org/reporting-checklists/spirit/info/#20b) | A stratified logrank test will be used to compare time to stable sputum culture conversion to negative status between the 2 regimens with the stratification factors country and severity of disease (AFB 3+ and/or bilateral cavitation). All subgroup analyses will be performed on the ITT, mITT and PP populations. Additionally, post hoc analyses not originally described in the protocol will be mentioned in the statistical analysis plan (SAP). SAP in supplementary material. | 11 |
| Statistics: analysis population and missing data | [#20c](https://www.goodreports.org/reporting-checklists/spirit/info/#20c) | Participants with Clinical Study Report (CSR) reportable deviations as evaluated and determined by a review committee prior to database lock will be excluded from the PP population. | 11 |
| **Methods: Monitoring** |  |  |  |
| Data monitoring: formal committee | [#21a](https://www.goodreports.org/reporting-checklists/spirit/info/#21a) | The DSMC is independent of TB Alliance and all project collaborators. It is governed by the DSMC Charter which describes its purpose and terms of reference. It consists of a chairperson and other seasoned TB disease and trial specialists, a statistician and a country representative from Tanzania (per Tanzania Medi requirements). The DSMC meeting will be held approximately every 6 months after the first randomised participant. Ad-hoc meetings can be called by TB Alliance or the DSMC based on the rates of SAEs, SAEs of particular concern, or any safety concerns that arise during the trial  The DSMC acts in an advisory capacity to TB Alliance, to safeguard the interest of trial participants by monitoring participant safety, participant risk versus benefit, and general evaluation of the study progress.  See DSMC Charter in supplementary material. | 12 |
| Data monitoring: interim analysis | [#21b](https://www.goodreports.org/reporting-checklists/spirit/info/#21b) | There will be 1 planned unblinded interim analysis which will contain results by treatment group in aggregate and will include the primary analysis. This will occur after all participants have completed 8 weeks of treatment. The study team and Data Safety Monitoring Committee (DSMC) will have access to the primary end-point analysis. | 11 |
| Harms | [#22](https://www.goodreports.org/reporting-checklists/spirit/info/#22) | Adverse events reporting applies to both investigational and control arms in the trial. All AEs and serious adverse events (SAEs) will be collected from the signing of the ICF until the follow-up Week 52 visit (end of trial).  Treatment-emergent AEs are defined as any AE that occurs after the first dose of IMP and within 28 days after the last dose of IMP. All AEs are recorded in the AE section of the DDC. AEs can be spontaneously reported or elicited during open-ended questioning, examination, or evaluation of a trial participant. The investigator must also promptly review all results of assessments performed as part of the trial, such as laboratory assessment results, ECGs, vital sign monitoring, physical examinations, etc. and assess them for clinical significance. Each AE is evaluated to determine the severity grade: Grade 1–4 as per the latest version of the DAIDS Severity Grading Scale, its duration (start and end dates or if continuing at the end-of-study visit), its relationship to the study treatment, action taken with respect to study treatment (treatment maintained, dose reduced, permanently discontinued, temporarily discontinued, not applicable), whether medication or therapy was taken/given in relation to the AE and whether it is a serious adverse event (SAE). All AEs will be followed until satisfactory clinical resolution or stabilization, or the end of the follow-up period or early discontinuation of the trial. All SAEs (including updated or significant follow up information) will be recorded and reported to TB Alliance immediately and within 24 hours of awareness.  TB Alliance has a legal responsibility to notify the relevant regulatory authority, IRB/EC, and investigators about the safety of an IMP under clinical investigation. | 12 |
| Auditing | [#23](https://www.goodreports.org/reporting-checklists/spirit/info/#23) | A risk-based approach is used for quality assurance audits to evaluate if the trial was conducted and the data generated in compliance with the protocol, GCP and applicable regulatory and ethics committee requirements.  Prior to the conduct of work, independent auditors conduct qualification audits for significant trial vendors to evaluate and confirm the vendor’s capability to perform the planned work in accordance with required standards. During the trial independent auditors conduct routine requalification audits. The frequency of audit is based on the risk tier assigned to each vendor, with the highest risk vendors subject to an audit every 2 years, ranging to the lowest risk vendors which are audited every 10 years. | 12-13 |
| **Ethics and dissemination** |  |  |  |
| Research ethics approval | [#24](https://www.goodreports.org/reporting-checklists/spirit/info/#24) | All relevant country ethics committee / institutional review board (REC/ IRB) approvals were obtained before commencement of this study.  The study protocol, the participant Information and Consent Form (ICF), the DDC, up-to-date versions of the Investigator Brochures or Summary of Product Characteristics (SmPC), as well as Principal Investigators qualifications has been submitted and approved by the ethical boards and regulatory authorities listed in Table 4. | 15 |
| Protocol amendments | [#25](https://www.goodreports.org/reporting-checklists/spirit/info/#25) | Once a protocol amendment has been finalized and signed by TB Alliance, the Principal Investigators would be provided with a copy of the protocol amendment and asked to sign and date the signature page, indicating their agreement to comply with the amendment.  A protocol amendment would be submitted to and approved by all the relevant ethics committees and regulatory authorities before implementation.  If the protocol amendment results in an update to the current informed consent form, this document would also need to be submitted to and approved by all the relevant ethics committees and regulatory authorities, at the same time. Following receipt of written approval, participants will be invited back to the site to review the updated informed consent form. After they have been provided with an opportunity to read and ask questions, they will be asked to sign the informed consent form at the same time as the investigator.  Trial registries would be updated timeously with the required information related to the amendment. |  |
| Consent or assent | [#26a](https://www.goodreports.org/reporting-checklists/spirit/info/#26a) | Informed consent forms are approved by the relevant ethics committee and regulatory authority. Written informed consent will be obtained from each screened participant, and the process is conducted in the participants’ preferred local languages in each country. In cases where a participant is illiterate, an impartial witness will be present throughout the informed consent process to ensure that the information in the consent form was accurately explained to, and understood by the participant, and that informed consent was freely given by the participant. | 6 |
| Consent or assent: ancillary studies | [#26b](https://www.goodreports.org/reporting-checklists/spirit/info/#26b) | Additional consents to be signed by participants are:   - Pharmacokinetic Sampling (for participants willing to participate in 24hrs of PK sample collection at Day 15, PK subgroup) - Biostorage for: - The storage of all unused blood, sputum, and urine samples (as available) for long-term storage. - Additional blood and urine samples for exploratory research will be collected at Day 1, weeks 4, 8, 15, and 26.   Pharmacogenetic testing for participants not randomised to the HRZE/HR arm to collect a blood sample at Day 1 for possible exploratory pharmacogenetic testing. | 6 |
| Confidentiality | [#27](https://www.goodreports.org/reporting-checklists/spirit/info/#27) | All laboratory specimens, including stored specimens, as well as trial reports, data collection tools, and administrative documents are identified by using only the participant’s unique trial number. All local and central databases are secured with password-protected access systems. The investigators ensure anonymity of the participant and that all documents are anonymised before being transmitted to TB Alliance. | 9 |
| Declaration of interests | [#28](https://www.goodreports.org/reporting-checklists/spirit/info/#28) | There are no financial or other conflicts of interest for principal investigators or DSMC committee members. | 16 |
| Data access | [#29](https://www.goodreports.org/reporting-checklists/spirit/info/#29) | The trial data will be made available after primary publication. All unpublished information/data given to the investigator by TB Alliance shall not be published or disclosed to a third party, other than to the responsible IRB/EC, with the understanding of the confidentiality of their nature, without the prior written consent of TB Alliance. | 15 |
| Ancillary and post-trial care | [#30](https://www.goodreports.org/reporting-checklists/spirit/info/#30) | Participants randomised to the 3 SPaL arms and the BPaL arm who relapse or experience treatment failure will be treated with HRZE/HR as part of the study, provided that drug susceptibility data do not reveal emergence of resistance to HRZE or the participant has a contraindication for receiving HRZE/HR, in which case they will be referred to the local NTP for further management. Participants who are re-treated will be followed for 26 weeks after the end of their re-treatment.  Participants randomized to standard of care/comparator arm who relapse, or experience treatment failure will be referred to local NTP for appropriate treatment with provision of medical report, last drug sensitivity result, most recent CXR to support participant management.  Participants who early discontinue from the study for reasons other than relapse/re-infection or treatment failure, where possible, would complete an early discontinuation visit and be referred to the local NTP. No additional follow-up visits are required except where unscheduled visits are needed for ongoing AEs that led to discontinuation from trial and for pregnancies.  TB Alliance certifies that it has liability insurance coverage for itself and will provide an associated certificate upon request. The insurance does not relieve the investigators of the obligation to maintain their own liability insurance as required by applicable law. TB Alliance does not assume any obligation for the medical treatment of other injuries and illnesses but in certain instances, compassionate support is provided to participants as required.  Provision of post-trial care {30}  Participants who early discontinue from the study for reasons other than relapse/re-infection or treatment failure, where possible, would complete an early discontinuation visit and be referred to the local NTP. No additional follow-up visits are required except where unscheduled visits are needed for ongoing adverse events (AEs) that led to discontinuation from trial and for pregnancies. | 4-5; 7 |
| Dissemination policy: trial results | [#31a](https://www.goodreports.org/reporting-checklists/spirit/info/#31a) | Results of this research will be submitted for publication as soon as feasible upon completion of the trial in the form of a joint publication(s) between TB Alliance and investigator(s), including site clinical and laboratory investigators, as appropriate. Publication and authorship will be in accord with the International Association of Journal Editors.  Because the trial is funded, in whole or in part, by the Bill and Melinda Gates Foundation (the “Foundation”), all peer-reviewed published research relating to the trial must comply with the Foundation’s Open Access Policy as described from time to time at http://www.gatesfoundation.org/How-We-Work/General-Information/Open-Access-Policy. Specifically, (a) all peer-reviewed published research relating to the trial must be submitted for publication by TB Alliance through the Chronos Open Access Publishing Service established by the Foundation to ensure the immediate and unrestricted access and reuse of all peer-reviewed published research funded, in whole or in part, by the Foundation without any embargo period, and (b) all data underlying the peer-reviewed published research results must be immediately made accessible and open to the public in accordance with the Foundation’s Open Access Policy. | 13 |
| Dissemination policy: authorship | [#31b](https://www.goodreports.org/reporting-checklists/spirit/info/#31b) | OM, BM, LL, NJ, TJ, BT, BR, HD, LA, BeM, EE, ML, HJ, SS, BnM, SE initiated the study design and implemented the study. OM is study physician and LL is clinical project manager. NJ is pharmacokinetics, BT is biologist, TJ is microbiologist, BeM is clinical data manager, EE is back up clinical project manager & medical writer, ML is drug supply manager, HJ oversees quality assurance, SS is community engagement manager. All authors contributed to refinement of the study protocol and approved the final manuscript. | 15 |
| Dissemination policy: reproducible research | [#31c](https://www.goodreports.org/reporting-checklists/spirit/info/#31c) | It is the intention that de-identified SDTM datasets including an associated data dictionary will be made available via TB PACTS hosted by CPATH (https://c-path.org/tools-platforms/tb-pacts/). The full protocol and statistical analysis plan will be made available as appendices during the publication of the trial results. | 11 |
| **Appendices** |  |  |  |
| Informed consent materials | [#32](https://www.goodreports.org/reporting-checklists/spirit/info/#32) | Informed consent materials in supplementary | 15 |
| Biological specimens | [#33](https://www.goodreports.org/reporting-checklists/spirit/info/#33) | For study participants not randomised to HRZE/HR and who sign a separate informed consent, a blood sample will be drawn at Day 1 (or at other time point during the trial if not obtained at Day 1) for possible exploratory pharmacogenetic testing. The sample may be used to identify genes that contribute to pharmacokinetic (PK) variability of sorfequiline and bedaquiline, i.e., variability in blood levels of those drugs and their metabolites, such as (but not necessarily restricted to) genes for drug-metabolizing enzymes or drug-transport proteins. | 9-10 |

None The SPIRIT Explanation and Elaboration paper is distributed under the terms of the Creative Commons Attribution License CC-BY-NC. This checklist can be completed online using <https://www.goodreports.org/>, a tool made by the [EQUATOR Network](https://www.equator-network.org) in collaboration with [Penelope.ai](https://www.penelope.ai)
