# Supplementary material for: Protocol for a phase 2, partially blinded, randomized trial assessing the safety and efficacy of sorfequiline or bedaquiline in combination with pretomanid and linezolid in adult participants with newly diagnosed, drug-sensitive, smear-positive pulmonary tuberculosis (NC-009)
Source: Trials. 2026 Jan 6;27:102. doi: 10.1186/s13063-025-09413-5 (PMC12869905; doi:10.1186/s13063-025-09413-5)
Supplement: Supplementary file 3 — Additional file 3. NC009 Statistical Analysis Plan. [file 13063_2025_9413_MOESM3_ESM.pdf]

STATISTICAL ANALYSIS PLAN

STUDY TITLE: A PHASE 2, PARTIALLY BLINDED, RANDOMISED TRIAL ASSESSING THE SAFETY AND EFFICACY OF TBAJ876 OR BEDAQUILINE, IN COMBINATION WITH PRETOMANID AND LINEZOLID IN ADULT PARTICIPANTS WITH NEWLY DIAGNOSED, DRUG-SENSITIVE, SMEAR-POSITIVE PULMONARY TUBERCULOSIS

PROTOCOL NUMBER: NC-009

SPONSOR:	TB Alliance<br>80 Pine Street, 20th Floor, New York, NY 10005
REGULATORY AGENCY IDENTIFIER NUMBER(S):	TBAJ876 IND #145349
PREPARED BY:	Rho<br>2635 E NC Hwy 54<br>Durham, NC 27713<br>Telephone: (919) 408-8000<br>Fax: (919) 408-0999

This document is confidential and proprietary to TB Alliance. This study is being conducted in compliance with good clinical practice, including the archiving of essential documents. Acceptance of this document constitutes agreement by the recipient that no unpublished information contained herein will be reproduced, published, or otherwise disclosed without the prior written approval of TB Alliance, except that this document may be disclosed to appropriate Institutional Review Boards under the condition that they keep the information confidential.

PROPRIETARY — © 2021 Rho, Inc.

ACKNOWLEDGEMENT AND SIGNATURE SHEET

**Approved:**	**Approved:**
Morounfolu Olugbosi<br>Senior Director, Clinical Development<br>TB Alliance	Patricia Stephenson<br>Associate Director, Biostatistics<br>Rho, Inc.
Signature and Date	Signature and Date
Signed by:<br>Olugbosi Morounfolu<br>Signer Name: Olugbosi Morounfolu<br>Signing Reason: I approve this document<br>Signing Time: 30-Sep-2024 | 11:39:43 AM EDT<br>3DABF6C3410E4B8F85E1D6E511FA5A5F	Signed by:<br>Patricia Stephenson<br>Signer Name: Patricia Stephenson<br>Signing Reason: I approve this document<br>Signing Time: 30-Sep-2024 | 11:36:23 AM EDT<br>3829D0C947B74A0A9E7F2FAEDE4550C3

PROPRIETARY — © 2021 Rho, Inc.

VERSION HISTORY

SAP Version	Version Date	Change(s)	Rationale
1.0	2024-01-18	Version 1.0	Version 1.0
2.0	2024-09-30	• General: References to 'CRF' changed to 'DDC' throughout document.<br>• Sections 5.4 and 7.4: Updates to reflect label changes for protocol deviations (e.g. major protocol deviations changed to CSR reportable deviations).<br>• Section 6.1: Note 2 methods for MIC (MGIT and EUCAST).<br>• Section 8.2.2: Additional detail provided for multivariate Cox proportional hazards model<br>• Section 8.2.3: Removal of multiple imputation sensitivity analysis.<br>• Section 9.2: Add summary of full regimens. Add Kaplan-Meier graphs for time to first linezolid discontinuation, pause, and/or reduction.<br>• Section 9.4: Specific toxicities updated.<br>• Section 9.5.1: Added eDish plot.<br>• Section 9.7: Update to only provide a listing for visual acuity.<br>• Section 9.9.1: Added shift table for vital signs.<br>• Section 12.4.1: Added section into Appendix to describe inability to produce sputum.<br>• Section 12.4.2.: Added section into Appendix to describe isolated positive cultures. Reference in Table 2.	Version 2.0

PROPRIETARY — © 2021 Rho, Inc.

		• Section 12.4.5: Added section for interpretation of relapse/reinfection using WGS.	

PROPRIETARY — © 2021 Rho, Inc.

TABLE OF CONTENTS

1. LIST OF ABBREVIATIONS....9
2. PURPOSE OF THE ANALYSES....11
3. PROTOCOL SUMMARY....12
4. GENERAL ANALYSIS AND REPORTING CONVENTIONS....15
5. ANALYSIS POPULATIONS....16
5.1. Intent-to-treat (ITT) Population....16
5.2. Safety Population....16
5.3. Modified Intent-to-treat (mITT) Population....16
5.4. Per Protocol (PP) Population....16
5.5. Pharmacokinetic (PK) Population....17
6. DISPOSITION OF PARTICIPANTS....18
6.1. Demographic and Other Baseline Characteristics....19
6.2. Medical History....21
7. STUDY OPERATIONS....22
7.1. Protocol Deviations....22
7.2. Randomisation....22
7.3. Measures of Treatment Compliance....22
8. ENDPOINT EVALUATION....23
8.1. Overview of Efficacy Analysis Methods....23
8.1.1. Multicenter Studies....23
8.1.2. Assessment Time Windows....23
8.1.3. Timing of Analyses....24
8.1.4. Multiple Comparisons/Multiplicity....24
8.1.5. Estimands....24
8.2. Primary Endpoint....25
8.2.1. Computation of the Primary Endpoint....25
8.2.2. Primary Analysis of the Primary Endpoint....27
8.2.3. Sensitivity Analyses of the Primary Analysis....28

PROPRIETARY — © 2021 Rho, Inc.

8.3. Key Secondary Endpoint 28
8.3.1. Proportion of participants with a favourable outcome at 26 weeks after EOT (B-Pa-L relative to 2HRZE/4HR) 28
8.4. Secondary Endpoints 31
8.4.1. Proportion of Participants who Meet the Criteria to Stop Treatment at Week 15 31
8.4.2. Proportion of participants with a favourable outcome at 26 weeks and 52 weeks after the EOT 32
8.4.3. Relapse Rates 32
8.4.4. Impact of Treatment Duration on Favourable Outcome and Relapse Rates (26 Weeks and 52 Weeks after EOT) 32
8.4.5. Bactericidal Activity 33
8.4.6. Time to Stable Sputum Culture Conversion to Negative Status by Severity Group and Separately by Country 34
8.5. Other Endpoints 34
8.6. Exploratory Endpoints 34
8.7. Examination of Subgroups 35
9. SAFETY EVALUATION 37
9.1. Overview of Safety Analysis Methods 37
9.2. Extent of Exposure 37
9.3. Adverse Events (AEs) 40
9.3.1. Adverse Event Overview 40
9.4. Deaths, Serious Adverse Events, and Specific Toxicities 42
9.5. Clinical Laboratory Evaluation 43
9.5.1. Analysis of Abnormal Laboratory Value 43
9.6. Electrocardiograms (ECGs) 45
9.7. Visual Acuity 46
9.8. Peripheral Neuropathy 46
9.9. Vital Signs, Physical Findings, and Other Observations Related to Safety 46
9.9.1. Vital Signs and Physical Examination 46

PROPRIETARY — © 2021 Rho, Inc.

9.9.2. Concomitant Medications 47
9.9.3. Concomitant Procedures 47
10. PHARMACOKINETIC EVALUATION 49
10.1. Pharmacokinetic Endpoints 49
10.2. Pharmacokinetic Methods 49
10.2.1. Pharmacokinetic Data Conventions 49
10.2.2. Pharmacokinetic Endpoint Estimation 50
10.2.3. Statistical Analysis of Pharmacokinetic Endpoints 51
10.2.4. Analyses of Efficacy versus Exposure 51
10.2.5. Analyses of Safety versus Exposure 52
10.2.6. Additional Analyses 53
11. INTERIM ANALYSES AND DATA MONITORING 54
12. APPENDIX 55
12.1. Study Flow Chart 55
12.2. Schedule of Events 56
12.3. Visit Windowing 57
12.4. Definitions 59
12.4.1. Inability to produce sputum 59
12.4.2. Isolated positive cultures 59
12.4.3. Positive culture 59
12.4.4. Derived MGIT results per visit 60
12.4.5. Interpretation of Relapse/Reinfection using Whole Genome Sequencing (WGS) 60

PROPRIETARY — © 2021 Rho, Inc.

LIST OF TABLES

Table 1: List of Abbreviations 9
Table 2: Definitions for Unassessable/Favourable/Unfavourable 28
Table 3: Parameters for Pharmacokinetic Analysis 51

PROPRIETARY — © 2021 Rho, Inc.

1. LIST OF ABBREVIATIONS

Table 1: List of Abbreviations

Abbreviation	Term
2HRZE/4HR	8 weeks (2 months) HRZE followed by 18 weeks (4 months) HR
3TC	Lamivudine
AE	adverse event
AFB	acid-fast bacilli
ALP	alkaline phosphatase
ALT	alanine aminotransferase
ARV	Antiretroviral
AST	aspartate aminotransferase
ATC	Anatomical Therapeutic Chemical
BA_{TTP}	bactericidal activity measured by TTP
BLQ	below the limit of quantitation
BMI	body mass index
B-Pa-L	bedaquiline, pretomanid, linezolid
CI	confidence interval
CRF	case report form
CSR	clinical study report
CTCAE	Common Terminology Criteria for Adverse Events
CV	coefficient of variation
DDC	Direct data capture
DMID	Division of Microbiology and Infectious Disease
DP	decimal places
DR-TB	drug-resistant tuberculosis
DS-TB	drug-sensitive tuberculosis
DST	drug susceptibility testing
DTG	Dolutegravir
ECG	Electrocardiogram
EORT	end of re-treatment
EOT	end of treatment
GCP	good clinical practice
HIV	Human Immunodeficiency Virus
HLT	high level term
HRZE	isoniazid, rifampicin, pyrazinamide, ethambutol
HR	isoniazid, rifampicin
ICH	International Council for Harmonisation

PROPRIETARY — © 2021 Rho, Inc.

IMP	investigational medication product
IRT	interactive response technology
ITT	Intent to Treat
LLOQ	lower limit of quantification
MAR	missing at random
MedDRA	Medical Dictionary for Regulatory Activities
MGIT	mycobacteria growth indicator tube
MIC	minimum inhibitory concentration
mITT	modified intent-to-treat
MTB	mycobacterium tuberculosis
NCA	non-compartmental analysis
NTP	National Tuberculosis Program
PK	Pharmacokinetic
PopPK	population pharmacokinetic
PP	Per Protocol
PR	PR interval – time from start of P wave to start of QRS complex on ECG
PT	Preferred term
QTcF	QT interval corrected for heart rate using Fridericia's formula
QRS	QRS complex (ventricular depolarisation) on ECG
RR	RR interval – time between two QRS complexes on ECG
SAE	serious adverse event
SAP	Statistical Analysis Plan
SD	standard deviation
SF	significant figures
SMQ	standardised MedDRA query
SOC	System Organ Class
TB	Tuberculosis
TBL	Total bilirubin
TEAE	treatment emergent adverse event
TFV	Tenofovir
TTP	time to positivity
ULN	upper limit of normal
WGS	whole genome sequencing

PROPRIETARY — © 2021 Rho, Inc.

2. PURPOSE OF THE ANALYSES

The purpose of this statistical analysis plan (SAP) is to provide detailed information to aid in the implementation of the statistical analysis and reporting of the study data for the planned Week 8 interim analyses (including the primary endpoint), the Week 52 after end of treatment (EOT) interim analysis, and the end of study analyses after the final database lock. Any additional planned analyses to support reporting for this study are covered by this SAP. These analyses will be used in the interim reports or in the clinical study report (CSR).

This SAP briefly summarises the protocol, describes the analysis sets, and the planned analyses. The details of the specific statistical methods that will be used for the pre-specified analyses of the primary and secondary endpoints are provided and were defined prior to unblinding of the data. This SAP does not limit the analyses in the interim reports or CSR, and additional analyses to supplement the interim report or CSR writing may be conducted and will be identified in the reports as post hoc. Table, figure, and listing specifications are provided in a separate document.

This SAP was written with due consideration of the recommendations outlined in the most recent International Council for Harmonisation (ICH) E9(R1) Guideline entitled Guidance for Industry: Statistical Principles for Clinical Trials and the most recent ICH E3 Guideline, entitled Guidance for Industry: Structure and Content of Clinical Study Reports.

PROPRIETARY — © 2021 Rho, Inc.

3. PROTOCOL SUMMARY

This is a phase 2, multi-center, partially blinded, randomised clinical trial conducted in 5 treatment arms.

The screening period will be up to 11 days. Participants who meet all of the inclusion criteria and none of the exclusion criteria will be randomised in a 1:1:1:1:1 ratio using an interactive response technology (IRT) that stratifies based on country and severity of disease (severe: acid-fast bacilli (AFB) 3+ and/or bilateral cavitation, non-severe: < AFB 3+ and no bilateral cavitation) to 1 of the 5 treatment arms:

- TBAJ876 25 mg + pretomanid 200 mg + linezolid 600 mg for 8 weeks followed by isoniazid (H) + rifampicin (R) (HR) for 7 to 18 weeks
- TBAJ876 50 mg + pretomanid 200 mg + linezolid 600 mg for 8 weeks followed by HR for 7 to 18 weeks
- TBAJ876 100 mg + pretomanid 200 mg + linezolid 600 mg for 8 weeks followed by HR for 7 to 18 weeks
- Bedaquiline 200 mg + pretomanid 200 mg + linezolid 600 mg for 8 weeks followed by bedaquiline 100 mg + pretomanid 200 mg + linezolid 600 mg for 18 weeks
- Isoniazid (H) + rifampicin (R) + pyrazinamide (Z), ethambutol (E) (HRZE) for 8 weeks followed by HR for 18 weeks (dose based on participant's weight) (2HRZE/4HR).

Treatment Period 1 (Day 1 through 8 weeks of treatment)

TBAJ876 and bedaquiline will be blinded during the first 8 weeks of trial treatment; participants randomised to the TBAJ876 or bedaquiline arms will receive open label pretomanid and linezolid. Participants randomised to the 2HRZE/4HR arm will receive open-label HRZE.

Treatment Period 2 (Week 9 through Week 15 or Week 26)

After receiving 8 weeks of treatment, participants randomised to the TBAJ876-Pa-L treatment arms will receive open-label HR for at least 7 weeks. Treatment completion will be allowed at Week 15 in participants randomised to the TBAJ876-Pa-L arms, if the below criteria are met:

- Week 8 or EOT Make-up Period 1 sputum mycobacteria growth indicator tube (MGIT) culture is negative, and
- The participant has no tuberculosis-related symptoms by Week 15. Participants with symptoms that have a more likely alternative explanation are eligible to complete treatment at Week 15.

If the MGIT result is mycobacterium tuberculosis (MTB) positive and/or there are still tuberculosis (TB) symptom(s), participants will continue to receive HR (in the 3 TBAJ876 arms) and will complete 18 weeks of treatment with HR, for a total of 26 weeks of treatment.

PROPRIETARY — © 2021 Rho, Inc.

After receiving 8 weeks of trial treatment, all participants randomised to the HRZE arm will receive open-label HR for 18 weeks, for a total of 26 weeks of treatment.

After receiving 8 weeks of treatment, participants randomised to the bedaquiline, pretomanid, linezolid (B-Pa-L) arm will receive open-label bedaquiline 100 mg (a reduction from the 200 mg daily dose in the first 8 weeks), pretomanid 200 mg, and linezolid 600 mg daily for 18 weeks, for a total of 26 weeks of trial treatment.

Follow-up Period (52 Weeks Post Treatment)

Participants in all treatment arms will complete efficacy and safety follow-up visits for 52 weeks after the end of treatment. The duration of trial participation after the ≤11-day screening period for participants in the TBAJ876-Pa-L treatment arms is expected to be 67 to 78 weeks (8-weeks Treatment Period 1, 7 to 18-weeks Treatment Period 2, and 52-weeks Follow-up Period). The duration of trial participation after the ≤11-day screening period for participants in the B-Pa-L or 2HRZE/4HR treatment arms is expected to be at least 78 weeks (8-week Treatment Period 1, 18-week Treatment Period 2, and 52-week Follow-up Period).

Dose Modification

Linezolid dose modifications (reductions and interruptions) are permitted if necessary for participant safety. Dose modifications of TBAJ876, bedaquiline, pretomanid, or HRZE are not allowed. At no time may the participant be treated with a single agent.

Full regimen interruptions will be allowed to a maximum of 14 cumulative doses during Treatment Period 1. The number of missed doses will be made up by a treatment extension starting at the end of Week 8.

The full regimen may be interrupted for up to 28 cumulative days during Treatment Period 2. The number of doses missed during Treatment Period 2 will be made up after Week 15 or Week 26 depending on the total treatment duration.

Retreatment

Participants randomised to the 3 TBAJ876-Pa-L arms and the B-Pa-L arm who relapse or experience treatment failure will be treated with 2HRZE/4HR, provided that drug susceptibility data do not reveal emergence of resistance to HRZE or the participant has a contraindication for receiving 2HRZE/4HR, in which case they will be referred to the local national tuberculosis program (NTP) for further management. Participants who are re-treated will be followed for 26 weeks after the end of their re-treatment.

In the case of treatment failure or relapse in participants randomised to the control arm (2HRZE/4HR), the participant will be referred to the local NTP for further management, and treating physicians will be provided with the drug susceptibility testing (DST) results and medical report from the investigator. Participants will be required to complete an Early Discontinuation Visit prior to referral to the local NTP.

PROPRIETARY — © 2021 Rho, Inc.

Treatment for Human Immunodeficiency Virus (HIV)

Participants living with HIV will be required to be treated with dolutegravir (DTG), tenofovir (TFV), and lamivudine (3TC) combination at least during the treatment phase of the trial in participants receiving TBAJ876-Pa-L (Treatment Period 1) and B-Pa-L (Treatment Period 1 and 2). Participants randomised to the 2HRZE/4HR regimen or receiving HR during Treatment Period 2 in the TBAJ876 arms can use any antiretroviral (ARV) compatible with the regimen.

Number of Participants (Planned):

The trial is planned to randomise at least 60 participants per treatment arm, for a total of at least 300 participants randomised.

PROPRIETARY — © 2021 Rho, Inc.

4. GENERAL ANALYSIS AND REPORTING CONVENTIONS

The following is a list of general analysis and reporting conventions to be applied for this study.

- Categorical variables will be summarised using counts (n) and percentages (%) and will be presented in the form n (%). If a count is 0, no percentage will be shown. To ensure completeness, summaries for categorical and discrete variables will include all categories, even if no participants had a response in a particular category.
- Continuous variables will be summarised using number of evaluable participants, mean, standard deviation (SD), minimum, maximum, and median. The mean, median, and confidence intervals (CI) will be rounded and reported to 1 more level of precision than the original observations, and the SD will be rounded and reported to 2 more levels of precision than the original observations. The minimum and maximum will be the same precision as the original data.
- Following SAS default rules, the median will be reported as the rounded average of the two middle numbers if the dataset contains even numbers.
- P-values will be rounded and reported to 3 decimal places if greater than 0.001. If the rounded p-value is less than 0.001, '<0.001' will be reported. If the rounded p-value is >0.999, '>0.999' will be reported. P-values and significance levels will be reported as 0.05 rather than .05.
- No preliminary rounding will be performed; rounding will only occur after analysis. To round, consider digit to right of last significant digit: if < 5 then round down, if ≥5 then round up.
- All listings will be sorted in order of treatment group, participant, and time of assessment (e.g., visit, time, and/or event).
- Dates in listings will be displayed as ddmmmyyyy (e.g., 25MAY2023).
- Age (in years) will be calculated using an imputed date of birth of 02JUL from the year of birth (typically the 183rd day of the year) and the Screening date in the following SAS algorithm: floor (Date of screening – Imputed date of birth]/365.25. In the analysis datasets, tables, and listings, age will be reported as the integer part of the derived age, with no rounding.
- Baseline will be the last non-missing value before study drug administration.
- In general, for efficacy analyses, if no scheduled visit data is available for the nominal direct data capture (DDC) recorded visits, the non-missing data from an early termination or unscheduled assessment performed closest to the target day and within the analysis window will be used.
- All analysis will be performed using the SAS System version 9.4 or higher.

PROPRIETARY — © 2021 Rho, Inc.

5. ANALYSIS POPULATIONS

5.1. Intent-to-treat (ITT) Population

The ITT population will include all randomised participants who took at least 1 dose of trial drug.

5.2. Safety Population

The safety population is the same as the ITT population. It will include all randomised participants who took at least 1 dose of trial drug. The term "Safety Population" will be used for all safety analyses, unless otherwise stated.

5.3. Modified Intent-to-treat (mITT) Population

The primary analysis population will be the mITT population defined as participants who are randomised and take at least 1 dose of trial drug, without late exclusions which are defined as:

- Lack of MTB culture positive on Day 1. Screening culture can be used to determine inclusion in mITT population if Day 1 sputum culture is contaminated or unavailable or,
- Discrepancies between screening rapid test result and corresponding culture or WGS result revealing drug-resistant TB (DR-TB). See Myco Discrepancy Evaluation Guidance Document.

The mITT will be the primary population for all efficacy analyses. For the other secondary endpoints i.e., Follow-up Week 26 and 52, 2 mITT analyses will be performed:

- Where all unassessable participants are excluded from the analysis (main analysis).
- Where all unassessable participants are considered unfavourable (sensitivity analysis).

Unassessable participants are defined in more detail in section 8.3.1 for each endpoint.

5.4. Per Protocol (PP) Population

The PP population is the mITT population excluding certain participants with CSR reportable deviations as evaluated and determined by a review committee prior to database lock as defined in section 7.1. For the primary endpoint, the PP population will also exclude participants who received less than 7 weeks of the regimen, referred to as PP 7 weeks. For all secondary endpoints, the PP population will exclude participants who received less than 80% of trial medication, referred to as PP80.

PROPRIETARY — © 2021 Rho, Inc.

5.5. Pharmacokinetic (PK) Population

The PK population will include participants who received at least one dose of TBAJ876, bedaquiline, pretomanid, or linezolid and have at least 1 valid measured concentration for any corresponding analytes (TBAJ876, TBAJ876-M2, TBAJ876-M3, bedaquiline, bedaquiline-M2, pretomanid, or linezolid) at a scheduled PK time point after the start of the dosing regimen of the respective drug. If any participants are found to be noncompliant with respect to dosing, or have incomplete data, a decision will be made on a case-by-case basis as to their inclusion in the analysis and subjected to the Sponsor's approval.

Observations and/or profiles on Day 15 and Week 8 may be excluded as not valid due to protocol deviations or events with potential to significantly affect the concentration data or the calculation of pharmacokinetic endpoints. Examples include, but may not be limited to, vomiting following oral dosing, sample processing errors that lead to inaccurate bioanalytical results, large deviations from planned dosing times, missed doses or incomplete dosing regimen leading up to sample collection. Exclusions will be made on a case-by-case basis and subjected to the Sponsor's approval.

PROPRIETARY — © 2021 Rho, Inc.

6. DISPOSITION OF PARTICIPANTS

The disposition of all consented participants will be tabulated by treatment group and overall. Participants who failed screening or were not randomised will only be included in the overall column. The following disposition information will be summarised:

- The number of participants consented (signed Informed Consent)
- The number and percentage of participants who failed screening and the reasons for failure
- The number of participants randomised
- The number of participants in the ITT
- The number and percentage of participants in the mITT, PP, PP 7 weeks, PP80, and PK populations
- The number and percentage of participants who completed the study through Week 8, through the end of Treatment Period 1 (including EOT make-up period 1 visit), through the end of Treatment Period 2 (including EOT make-up period 2 visit), through the end of 26 Weeks Post EOT Follow-up, and through the end of 52 Weeks Post EOT Follow-up
- The number and percentage of participants who discontinued the study early and the reasons for withdrawal overall and by study treatment period and during the follow up period
- The number and percentage of participants who enter re-treatment
- The number and percentage of participants who complete re-treatment (including the re-treatment follow-up period)

Percentages for the number of participants who failed screening and the reasons for failure will be based on the number of consented participants. All other percentages will be based on number of participants in the ITT population.

Enrollment over time will be summarized graphically.

Participant disposition data for all randomised participants will also be listed. A separate by-participants listing for screen failures will be generated along with reason for screen failure.

PROPRIETARY — © 2021 Rho, Inc.

6.1. Demographic and Other Baseline Characteristics

Descriptive statistics for demographic and other baseline characteristics will be summarised by treatment group and overall, for the ITT population. Select demographics and baseline characteristics will also be summarized by region (Africa, Asia, Europe). Characteristics to be summarised include:

Demographics: age (years), country, region (Africa, Asia, Europe), race, ethnicity, sex at birth, current gender identity, height (cm), weight (kg), and body mass index (BMI) (kg/m^2). Note that for participants from Tanzania and Uganda, the reported sex at birth will be used for both sex at birth and current gender identity.

Baseline disease characteristics:

- Smoking status (Never, Current, Former)
- Alcohol status (Never, Current, Former)
- Screening mycobacteriology test results
    - AFB microscopy (no AFB seen, scanty, 1+, 2+, 3+)
    - GeneXpert MTB/XDR
        - MTB confirmation (yes/no)
        - Isoniazid (susceptible/resistant/indeterminate)
    - GenoType MTBDRplus
        - MTB confirmation (yes/no)
        - Isoniazid (susceptible/resistant/indeterminate)
        - Rifampicin (susceptible/resistant/indeterminate)
    - GeneXpert MTB/RIF Ultra
        - MTB confirmation (yes/no)
        - Rifampicin (susceptible/resistant/indeterminate)
        - Semi-quantitative result (high, medium, low, very low, trace)
        - Minimum cycle threshold (continuous)
    - MGIT culture
        - Result (positive, negative, missing)
        - Time to positivity, when positive for MTB complex (continuous)
    - Lipoarabinomannan assay
        - Done/Not done
        - pg/mL (continuous)
- Baseline mycobacteriology test results (the most severe result will be reported for categorical assessments; mean for continuous assessments)

PROPRIETARY — © 2021 Rho, Inc.

- AFB microscopy (no AFB seen, 1+, 2+, 3+)
- GeneXpert MTB/RIF Ultra
  - MTB confirmation (yes/no)
  - Rifampicin (susceptible/resistant/indeterminate)
  - Semi-quantitative result (high. medium, low, very low, trace)
  - Minimum cycle threshold (continuous)
- MGIT culture
  - Result (positive, negative, missing)
  - Time to positivity, when positive for MTB complex (continuous)
- Lipoarabinomannan assay
  - Done/Not done
  - pg/mL (continuous)
- MGIT drug susceptibility testing
  - Isoniazid (susceptible, resistance, missing)
  - Rifampicin (susceptible, resistance, missing)
  - Streptomycin (susceptible, resistance, missing)
  - Ethambutol (susceptible, resistance, missing)
  - Pyrazinamide (susceptible, resistance, missing)
  - Pretomanid (susceptible, resistance, missing)
  - Linezolid (susceptible, resistance, missing)
- MIC (minimum inhibitory concentration) for bedaquiline, pretomanid and TBAJ876, by MGIT and EUCAST methods
- Genotypic drug susceptibility testing
  - Isoniazid (susceptible, resistance, indeterminate)
  - Rifampicin (susceptible, resistance, indeterminate)
  - Streptomycin (susceptible, resistance, indeterminate)
  - Ethambutol (susceptible, resistance, indeterminate)
  - Pyrazinamide (susceptible, resistance, indeterminate)
  - Bedaquiline (susceptible, resistance, indeterminate)
  - Pretomanid (susceptible, resistance, indeterminate)
  - Linezolid (susceptible, resistance, indeterminate)

- Serology
  - HIV status (positive, negative as collected in DDC)
  - CD4 count (summary statistics)
  - Antiretroviral therapy (yes, no)

- Karnofsky performance status score (%)

- Chest x-ray result (normal, abnormal)

PROPRIETARY — © 2021 Rho, Inc.

- Consistency with TB (yes, no)
- Cavities (unilateral, bilateral)

- Severity of disease (severe: AFB 3+ and/or bilateral cavitation, non-severe: < AFB 3+ and no bilateral cavitation)
- Ophthalmology history
  - Personal history of vision and/or eye disorder (yes, no)
  - Personal history or prior eye surgery (yes, no)
  - History of eye trauma (right eye; left eye) (yes, no)

All durations are calculated relative to the date of first study drug administration.

Demographic and baseline characteristics data will be listed for participants in the ITT population.

6.2. Medical History

Medical history will be coded using the latest available version of Medical Dictionary for Regulatory Activities (MedDRA). The number and percentage of participants with medical history will be summarised by system organ class (SOC) and preferred term (PT) for the ITT population.

Medical history will also be listed for all participants in the ITT population.

PROPRIETARY — © 2021 Rho, Inc.

7. STUDY OPERATIONS

7.1. Protocol Deviations

Prospective approval of protocol deviations to randomisation criteria, also known as protocol waivers or exemptions, is not permitted.

Protocol deviations will be presented by the following 3 categories:

1) CSR reportable deviations. These deviations may have a significant effect on the scientific value of the trial

2) Non-CSR reportable deviations

3) Serious good clinical practice (GCP) non-compliance deviations

CSR reportable deviations and serious GCP non-compliance deviations will be listed for participants in the ITT population.

Participants with CSR reportable deviations will be included in the mITT but may not be included in the PP analyses (see Section 5.4). CSR reportable deviations (blinded to treatment arm) will be evaluated by a review committee prior to database lock; the committee will document which CSR reportable deviations should be excluded from any of the PP analyses.

The number and percentage of participants with any CSR reportable deviation and by each CSR reportable deviation type will be presented by treatment group and overall, in the ITT population.

7.2. Randomisation

Participants will be randomised to one of the 5 regimens in a 1:1:1:1:1 ratio, using an IRT system which will stratify based on country (Georgia, Philippines, South Africa, Tanzania, Uganda) and severity of disease (severe: AFB 3+ and/or bilateral cavitation, non-severe: < AFB 3+ and no bilateral cavitation) to allocate participants evenly across the regimens by country and severity of disease burden. Information and directions will be provided to each site via the IRT user manual. TBAJ876 and bedaquiline will be blinded, while pretomanid, linezolid, and HRZE will be open label in Treatment Period 1. Treatment Period 2 is open label for all treatment regimens, although the dose of TBAJ876 will remain blinded throughout the study for the 3 TBAJ876 arms.

7.3. Measures of Treatment Compliance

See section 9.2 for details on the analysis of treatment compliance.

PROPRIETARY — © 2021 Rho, Inc.

8. ENDPOINT EVALUATION

8.1. Overview of Efficacy Analysis Methods

To support the reporting for this study, analyses will be performed during the study based on snapshots of the database at key time points of interest as specified in Section 11 (e.g. Week 8 analysis [primary analysis], 52 weeks after EOT, etc.) in addition to the end of study analysis after the final database lock. Unless otherwise specified, data analyzed at these milestones will include data up to and including the time point of interest:

- The primary efficacy analyses summarised after all participants have completed Week 8 will include all data up to and including Week 8. One exception is that if the first negative result occurs at Week 8, the Week 9 culture result will be used for the confirmatory negative result
- Efficacy analyses summarised after all participants have completed the 52-week follow-up period after treatment will include all data up to and including the 52-week follow-up period after treatment.

8.1.1. Multicenter Studies

This is a multi-center study. Unless stated otherwise, data from all participating centers in the study will be pooled for analyses.

8.1.2. Assessment Time Windows

For by-visit summaries, the nominal visit will be used for analysis. Unless otherwise specified, for on-treatment tables for by-visit safety data (e.g. vital signs shift to worst), on-treatment assessments up to Week 26, EOT make-up assessments, and follow-up visits are included (e.g. excludes re-treatment visits). Unscheduled visits will be listed but will not be displayed separately in the summaries.

Analysis windows listed in the table in Section 12.3 of the Appendix will be used in the efficacy analyses for slotting actual efficacy assessments into the planned assessment schedule by study day. Early termination visit and unscheduled visit efficacy data will be included in the analyses if no scheduled visit data is available for the nominal DDC visit. If more than one unscheduled assessment (early termination or unscheduled) is performed within an analysis window, the assessment performed closest to the target day will be used, with the earlier assessment given priority in a tie. Analysis windows will not be used for the re-treatment periods of the study. Early termination visit and unscheduled visit efficacy data from study days up to and including the last date of dosing for treatment period 1, including make-up doses, will be assigned to treatment period 1. Early termination visit and unscheduled visit efficacy data between the first date of dosing for treatment period 2 and the participant's EOT date will be assigned to treatment

PROPRIETARY — © 2021 Rho, Inc.

period 2. Early termination visit and unscheduled visit efficacy data between the participant's EOT date and EOT + 378 will be assigned to the post EOT follow-up period.

8.1.3. Timing of Analyses

See Section 11 for details regarding the planned interim analysis and potential other analyses performed during the trial.

8.1.4. Multiple Comparisons/Multiplicity

Statistical testing for the primary endpoint will be performed using the Hochberg method to control the overall Type I error rate at 5% for the following comparisons: TBAJ876 high dose vs. HRZE, TBAJ876 medium dose vs. HRZE, and TBAJ876 low dose vs. HRZE.

No attempt will be made to adjust for multiplicity when testing secondary and exploratory endpoints. All secondary and exploratory endpoints subjected to significance testing will be assessed at an unadjusted 2-sided level of significance of 0.050.

8.1.5. Estimands

An estimand is the target of estimation to address the scientific question of interest posed by a study objective. The 4 attributes of an estimand include the target population, variable of interest, population-level summary of the endpoint, and specification of how intercurrent events are reflected in the scientific question of interest.

Intercurrent events occur after treatment initiation and either preclude observation of the endpoint or affect its interpretation. Study drug discontinuation before the time point of interest defining the endpoint is considered an intercurrent event. For safety and pharmacokinetic (PK) objectives, study drug discontinuation is handled with a "while-on-treatment strategy," i.e., response to treatment prior to the occurrence of the intercurrent event of interest, such that all observed values of the endpoint of interest are used prior to study drug discontinuation.

Refer to Section 5 for analysis populations that are used to assess endpoints.

Data Sources for Endpoints:

- MGIT results are captured in DDC.
- Laboratory test results are from an external laboratory.
- Various data points for determining unassessable vs unfavourable outcomes are captured in DDC.

PROPRIETARY — © 2021 Rho, Inc.

8.1.5.1. Primary Objective Estimand

- Target population: The analysis population will be mITT adult participants with newly diagnosed, smear-positive, pulmonary DS-TB as defined by the protocol inclusion/exclusion criteria.
- Variable of interest: time to stable sputum culture conversion to negative status over 8 weeks using data from weekly cultures up to and including Week 8.
- Population-level summary: comparison of time to stable sputum culture to negative status between each TBAJ876 treatment group and 2HRZE/4HR.
- Intercurrent event handling:
  - Hypothetical strategy: Participants who discontinue the study/lost to follow-up or die due to any cause prior to 8 weeks without having met the criteria for stable sputum culture conversion will be censored at the date of their last visit.

8.1.5.2. Key Secondary Objective Estimand

- Target population: The analysis population will be mITT adult participants with newly diagnosed, smear-positive, pulmonary DS-TB as defined by the protocol inclusion/exclusion criteria.
- Variable of interest: Proportion of participants with a favourable outcome at 26 weeks after EOT.
- Population-level summary: comparison of proportions of participants with a favourable outcome between the B-Pa-L and 2HRZE/4HR treatment groups.
- Intercurrent event handling:
  - Composite strategy:
    - Participants who are unassessable as defined in Table 2 in section 8.3 will be excluded for the main analysis. A sensitivity analysis will be conducted where unassessable participants will be considered unfavourable.

8.2. Primary Endpoint

The primary endpoint is time to stable sputum culture conversion to negative status over 8 weeks using data up to and including Week 8.

8.2.1. Computation of the Primary Endpoint

Stable sputum culture conversion to negative status is defined as 2 MTB negative results (at least 7 days apart without an intervening MTB positive). If the first negative result occurs at Week 8, the Week 9 culture result will be used for the confirmatory negative result. However, the date of

PROPRIETARY — © 2021 Rho, Inc.

meeting stable sputum culture conversion to negative status will be on the date of the initial Week 8 negative result.

Definition for MTB positive and how to derive the MGIT result at a visit are provided in Appendix (Sections 12.4.3 and 12.4.4).

Time to negative stable sputum culture conversion is defined as the time from the date of randomisation to the date of the first of two negative cultures. Time to stable sputum culture conversion (weeks) is calculated as:

$$(\text{date of first of two negative cultures} - \text{randomisation date} + 1)/7.$$

For participants who complete 8 weeks but do not achieve stable sputum culture conversion status, time will be taken from randomisation to 8 weeks (and censored). Participants who die or are lost to follow up/withdrawn will be considered as not achieving culture negative status unless they have achieved a culture negative status prior to death or being lost to follow up/withdrawn. The last known visit date will be taken for any participants who are missing the date of their 8-week visit.

PROPRIETARY — © 2021 Rho, Inc.

The event and censoring rules are summarised below.

Situation	Date of Event or Censoring	Outcome
No post-baseline sputum assessments (i.e., discontinue prior to the first sputum assessment)	Randomisation	Censored
Participants with two negative cultures (7 days apart without an intervening positive culture) within 8 weeks	Date of the first of the 2 negative cultures	Event (Stable sputum culture conversion to negative)
Participants with first negative culture at Week 8 with confirmatory negative culture at Week 9	Date of the Week 8 negative culture	Event (Stable sputum culture conversion to negative)
Participants who die due to any cause prior to Week 8 sputum collection without having met the criteria for stable sputum culture conversion	Date of the last visit	Censored
Participants who discontinued study prior to Week 8 sputum collection without having met the criteria for stable sputum culture conversion	Date of the last visit	Censored
Participants who complete 8 weeks without meeting the criteria for stable sputum culture conversion	Date of 8-week visit	Censored

8.2.2. Primary Analysis of the Primary Endpoint

The primary efficacy analysis uses a time to event approach to compare culture conversion to negative status between the investigational regimens and control regimen based on the mITT population. A stratified logrank test will be used to compare time to stable sputum culture conversion to negative status between the 2 regimens with the stratification factors country and severity of disease (severe: AFB 3+ and/or bilateral cavitation, non-severe: < AFB 3+ and no bilateral cavitation).

Estimates of the hazard ratios will be calculated using a stratified Cox proportional hazards model with treatment group as the sole covariate and the stratification factors country and severity of disease. Corresponding 2-sided 95% CIs will also be presented.

Time to stable sputum culture conversion to negative status will also be summarised using the Kaplan-Meier method and displayed graphically. Median event times (and other quartiles) and 2-sided 95% CI will be provided.

PROPRIETARY — © 2021 Rho, Inc.

A multivariate Cox proportional hazards model will be used to explore predictors (e.g. baseline characteristics) associated with time to stable sputum culture conversion to negative status. Univariate modelling will first be conducted to identify potential predictors for consideration in the multivariate model using a p-value threshold of 0.10. Predictors considered for inclusion will include those listed in Section 8.7 in addition to TTP, LAM concentration, and cycle threshold. Continuous covariates including those such as age which were split into categories for subgroup analysis in Section 8.7 will be treated as continuous for these analyses. The hazard ratio associated with each of the baseline covariates will be presented along with associated 95% CIs and p-values.

The analysis of the primary endpoint will be repeated using the ITT population.

A supportive table of enrollment and analysis exclusions from the MITT population will be generated summarizing the number of enrolled participants with late exclusions including type of late exclusion and the number of these participants who are not treated.

8.2.3. Sensitivity Analyses of the Primary Analysis

A sensitivity analysis pooling all TBAJ876 regimens will also be conducted.

Analysis of the primary endpoint will be repeated for the PP 7 weeks population.

Subgroup analyses by stratification factors (country and disease severity) as well as other key subgroups will be performed and are described in Section 8.7.

Primary endpoint status, time to stable sputum culture conversion to negative status in weeks, and conversion status by visit in Treatment Period 1 and for week 9 (where applicable to the primary endpoint) will be listed for all participants in the mITT population.

8.3. Key Secondary Endpoint

8.3.1. Proportion of participants with a favourable outcome at 26 weeks after EOT (B-Pa-L relative to 2HRZE/4HR)

- For this analysis, participants will be classified as having an unassessable, favourable, or unfavourable outcome at 26 weeks after EOT, as applicable.

The table below describes the definitions:

Table 2: Definitions for Unassessable/Favourable/Unfavourable

Unassessable	A participant who has not already been classified as unfavorable will be classified as unassessable if any one of the following conditions is met:<br><br>• Participants who, having completed treatment, are lost to follow-up or withdrawn from the study & their last status was culture negative

PROPRIETARY — © 2021 Rho, Inc.

	and their last positive culture result ("isolated positive culture") was followed by at least two negative culture results at different visits (at least 7 days apart, without an intervening positive culture)<br><br>• Women who become pregnant during treatment and stop their allocated treatment<br><br>• Participants with suspected/confirmed infection with disease causing an ongoing pandemic during treatment and who stop their allocated treatment<br><br>• Participants who died during treatment from violent or accidental cause (e.g., road traffic accident). This does not include death from suicide, which will be considered as an unfavourable outcome.<br><br>• Participants who died during follow-up (after the end of treatment) with no evidence of failure or relapse of their TB, their last status being culture negative<br><br>• Re-infection<br><br>• Participants who are able to produce sputum at the endpoint visit, but whose endpoint visit sputum samples are all contaminated or missing, who cannot be brought back for repeat cultures, provided their last positive culture ("isolated positive culture") was followed by at least two negative cultures
Assessable participants can be classified as favourable or unfavourable as described below.	
Favourable	Participants who maintain stable culture negative status through to 26 weeks after EOT without re-treatment for TB
Unfavourable	Assessable participants that do not meet the definition for a favourable outcome above are classified as unfavourable defined as:<br><br>• Participants who are not culture negative status at the time of the endpoint (26 weeks after EOT) and whose last positive culture result was not followed by at least two negative culture results. Note that:

PROPRIETARY — © 2021 Rho, Inc.

	○ Participants who have had surgery and the resected tissue is cultured and is positive for MTB are classified as unfavourable.<br>○ Participants requiring an extension of their treatment beyond that permitted by the protocol i.e. a restart or a change of treatment for any reason except reinfection or pregnancy, will be classified as unfavourable (even if participant has a stable culture negative result at end point)<br>• Participants dying from any cause during treatment, except from violent or accidental cause (e.g. road traffic accident), not including suicide (i.e., suicide will be considered an unfavourable outcome), or<br>• Participants definitely or possibly dying from TB related cause during the follow-up phase, or<br>• Participants lost to follow up or withdrawn from the study before the end of treatment

Two mITT analysis will be performed:

1. Where all unassessables are excluded from the analysis (main analysis).
2. Where all unassessables are unfavourable (sensitivity analysis).

Participants who discontinue the study/lost to follow-up or die prior to 26 weeks will be categorized as described in Table 2 as well as based on whether the main or sensitivity analysis is being conducted as described above.

For both mITT analyses, the number and proportion of participants with a favourable outcome and corresponding 95% Wilson (score) CIs will be summarised for the B-Pa-L and 2HRZE/4HR arms at week 26 after EOT. For the main analysis (where unassessables are excluded), the number and proportion of unassessables will also be displayed separately.

A stratified analysis of the risk difference adjusting for country and severity of disease using Cochran-Mantel-Haenszel weights will be used to compare B-Pa-L with 2HRZE/4HR. Stratum-adjusted risk differences and corresponding 95% CIs will be provided for B-Pa-L vs. 2HRZE/4HR. Analyses unadjusted for stratification variables will also be presented.

Logistic regression modelling will also be used to explore predictors of achieving a favourable outcome (e.g., baseline characteristics).

PROPRIETARY — © 2021 Rho, Inc.

Further analyses will explore the relationship between the primary endpoint, time to stable sputum culture over 8 weeks, and whether or not a participant achieves a favourable outcome. For example, Kaplan-Meier estimates and graphs for time to stable sputum culture will be generated for participants who achieve a favourable outcome and participants who do not achieve a favourable outcome. In addition, the Cox proportional hazards model for time to stable sputum culture will be extended to include a covariate for participants achieving a favourable outcome (yes/no).

Analysis of the key secondary endpoint will be repeated for the PP80 population.

An additional sensitivity analysis may be conducted treating all deaths as unfavourable while excluding the remaining unassessable outcomes.

The unadjusted and adjusted risk differences for each of the analyses of the key secondary endpoint will be summarised graphically in a forest plot.

Outcome (favourable, unfavourable, unassessable) at 26 Weeks after EOT and 52 Weeks after EOT as well as relapse status (yes, no) will be listed for all participants the mITT population.

8.4. Secondary Endpoints

The primary population of interest for secondary endpoints is the mITT population. Analyses will be repeated for the PP80 population.

8.4.1. Proportion of Participants who Meet the Criteria to Stop Treatment at Week 15

Per protocol, treatment completion will be allowed at Week 15 in participants randomised to the TBAJ876-Pa-L arms, if the below criteria are met:

- Week 8 or EOT Make-up Period 1 sputum MGIT culture is negative, and
- The participant has no TB-related symptoms by Week 15. Participants with symptoms that have a more likely alternative explanation are eligible to complete treatment at Week 15.

Criteria to stop treatment at Week 15 will be determined through investigator decision (i.e., patient management) for participants in TBAJ876-Pa-L arms.

Inability to produce sputum under experienced staff will be considered as a negative MGIT culture result. If Week 8 MGIT culture results are inconclusive (i.e., contamination of sputum sample), the culture sample obtained at Week 9 can be used to determine whether a participant met the criteria. If a participant has a negative stable MGIT culture result by Week 8 but discontinues prior to Week 15, the participant will be classified as unassessable for this analysis. If MGIT result is MTB positive and/or there are still TB symptom(s), participants will be classified by the investigator as not meeting the criteria for stopping treatment.

The proportion of participants in each TBAJ876 treatment group who meet the above criteria to stop treatment at Week 15, and corresponding 95% Wilson (score) CIs, will be presented based

PROPRIETARY — © 2021 Rho, Inc.

on the mITT population. This analysis will be conducted in 2 ways. The main analysis will include participants who met criteria to stop at Week 15 and did not stop, and participants who did not meet criteria to stop but stopped (e.g. classify and include participants based on meeting the criteria regardless if they stop or not per protocol). If there are such participants, a second analysis will be performed that excludes these participants.

A listing of stopping criteria, whether or not participants met criteria to stop at Week 15, and whether they stopped treatment will be provided. This listing will flag participants who met criteria to stop at Week 15 and did not stop as well as participants who did not meet criteria to stop but stopped.

Analyses of the proportion of participants who meet the criteria to stop treatment at Week 15 will be similar to that of the key secondary endpoint in Section 8.3.

In addition, a dose response analysis using the Cochran-Armitage test for trend using PROC FREQ within SAS and specifying the TREND option on the TABLES statement will be conducted to evaluate if the proportion of participants meeting the criteria to stop treatment at Week 15 increases linearly with increasing dose level of TBAJ876 (25 mg, 50 mg, 100 mg) adjusting for dose level as a categorical covariate.

8.4.2. Proportion of participants with a favourable outcome at 26 weeks and 52 weeks after the EOT

The proportion of participants with a favourable outcome at 26 weeks and 52 weeks after EOT will be analyzed for the 3 dose levels of TBAJ876 relative to 2HRZE/4HR similar as described for B-Pa-L vs. 2HRZE/4HR in Section 8.3.

In addition, the proportion of participants with a favourable outcome at 52 weeks after EOT will be analyzed for B-Pa-L vs. 2HRZE/4HR as described in Section 8.3.

8.4.3. Relapse Rates

Relapse rates at 26 weeks after EOT, and separately at 52 weeks after EOT (end of follow-up period) based on the mITT population will be summarised as described for favourable outcome by treatment group. Participants with relapse are classified based on the definition in Protocol Table 17.

8.4.4. Impact of Treatment Duration on Favourable Outcome and Relapse Rates (26 Weeks and 52 Weeks after EOT)

To support the main analyses of favourable outcome and relapse rates performed based on the randomised treatment arms and further explore the impact of treatment duration on efficacy, analyses will be performed for 2 subgroups for each experimental treatment arm:

(1) participants stopping treatment at Week 15 because stopping criteria having been met, and

PROPRIETARY — © 2021 Rho, Inc.

(2) participants not meeting the criteria to stop treatment (continuing treatment to Week 26).

Participants who met criteria to stop at Week 15 and did not stop and participants who did not meet criteria to stop but stopped will be excluded from analysis.

In an exploratory fashion, analyses will be conducted to descriptively compare these subgroups within each TBAJ876-Pa-L arm to examine whether treatment duration impacts effect of TBAJ876 for a particular dose, as well as to compare these subgroups between the TBAJ876 dose arms to examine whether the impact of treatment duration varies by TBA876 dose.

The resulting subgroups by arm and treatment are below:

- TBAJ876 25 mg-Pa-L, met the criteria to stop treatment at Week 15
- TBAJ876 25 mg-Pa-L, did not meet the criteria to stop treatment at Week 15
- TBAJ876 50 mg-Pa-L, met the criteria to stop treatment at Week 15
- TBAJ876 50 mg-Pa-L, did not meet the criteria to stop treatment at Week 15
- TBAJ876 100 mg-Pa-L, met the criteria to stop treatment at Week 15
- TBAJ876 100 mg -Pa-L did not meet the criteria to stop treatment at Week 15

Analysis of favourable outcome as described in Section 8.3 will be performed within each of the aforementioned subgroups. Similarly, analysis of relapse rates as described in Section 8.4.3 will be performed by subgroup. The results of these subgroup analyses will also be displayed graphically.

Logistic regression modelling will be used to further explore the relationship between favourable outcome and early completion of treatment, and separately relapse rates and early completion of treatment. The logistic model for favourable outcome will focus on TBAJ876 participants and will include treatment group, early completion of treatment group (stop at Week 15 vs did not stop at Week 15), treatment group by early completion of treatment group interaction, and the stratification factors.

8.4.5. Bactericidal Activity

To assess the bactericidal activity over 2 weeks of TBAJ876 or bedaquiline in combination with pretomanid and linezolid, relative to HRZE, in adult participants with newly diagnosed, smear-positive, pulmonary DS-TB, analyses will be performed to calculate bactericidal activity over 2 weeks (BA_{TTP}(1-15)) as determined by the rate of change in TTP over Days 1 to 15 of treatment.

The rate of change in TTP over Days 1 to 15 will be calculated as:

$$\frac{BA_{TTP}(15) - BA_{TTP}(1)}{15 - 1}$$

If a participant does not have a bactericidal activity measured by TTP (BA_{TTP}) result at Day 15 (BA_{TTP}(15)), then their closest result prior to Day 15, including unscheduled or early termination

PROPRIETARY — © 2021 Rho, Inc.

visits, will be used instead, and the rate of change will be calculated over the number of days between that result and Day 1. If a participant does not have a $BA_{TTP}(1)$ value, then the calculation will not be performed. The rate of change in TTP over Days 1 to 15 will be summarised descriptively and presented by treatment group for the mITT population.

Data listings and plots of the observed TTP counts over time will be presented accordingly for each collection visit. Observed mean TTP counts (in data listings and plots) will be presented for the mITT population.

A similar analysis will be performed to assess bactericidal activity over 8 weeks ($BA_{TTP}(1\text{-}56)$).

8.4.6. Time to Stable Sputum Culture Conversion to Negative Status by Severity Group and Separately by Country

Time to stable sputum culture conversion to negative status through 8 weeks of treatment will also be analyzed as described in Section 8.2.2 for the primary analysis by severity group (severe, non-severe) and separately by country.

8.5. Other Endpoints

Time to stable sputum culture conversion to negative status over 8 weeks comparing B-Pa-L vs. HRZE will be analyzed as described in Section 8.2.2 for the primary analysis.

Time to stable sputum culture conversion to negative status over 8 weeks comparing TBAJ876 vs. B-Pa-L will also be analyzed as described in Section 8.2.2 for the primary analysis.

To evaluate stable sputum culture conversion to negative status over time of TBAJ876 or B-Pa-L relative to 2HRZE/4HR during the treatment period, the estimated probability of stable sputum culture conversion to negative status based on the Kaplan-Meier method will be presented for each treatment regimen at Weeks 4, 6, 8, 12, 15, 20, and 26 for the mITT population. In addition, the proportion of patients with stable sputum culture conversion will be presented by visit including Weeks 4, 6, 8, 12, 15, 20, 26, EOT Treatment Period 1, EOT Treatment Period 2, and follow-up visits. Weeks 20 and 26 in the TBAJ876 arms, participants who stop at Week 15 will be considered unassessable for this analysis and presented as a separate row in the table.

The TB Symptoms Profile will record participants' ratings of the severity of common TB symptoms. TB Symptoms Profile responses and shifts from baseline to maximum category will be summarised and presented by treatment group for the ITT population.

8.6. Exploratory Endpoints

Unless otherwise stated, all exploratory endpoints will be performed on the mITT population.

For patients who are re-treated, the following descriptive analyses will also be performed:

PROPRIETARY — © 2021 Rho, Inc.

- Time to treatment failure or relapse
- Proportion of participants with negative stable MGIT culture after re-treatment and after Week 26 follow-up.

Time to treatment failure or relapse in weeks is calculated as (Date of treatment failure or relapse – Date of randomisation + 1)/7.

Change from baseline in measurements of biomarker assays (potentially LAM and other assays), through the course of treatment and the post-treatment follow-up period relative to treatment outcome will be evaluated and presented by treatment group.

Additional analyses will be conducted to correlate response measured with MGIT at Day 1 and Weeks 4, 8, 15, and 26 with AFB smear and exploratory biomarkers.

Correlation analyses will also be performed to look at the relationship of MGIT culture results at Weeks 2, 4, and 8 with favourable outcome at 26 weeks and 52 weeks after the EOT.

Logistic regression modelling will be used to explore predictors (i.e., baseline characteristics) of MGIT negative culture (yes/no) at Week 8 of treatment.

Quality of life data will be collected using the WHOQOL-BREF. Summary statistics for observed and change from baseline results will be reported by visit and treatment group for the mITT population, and by sex at birth and current gender identity subgroups.

8.7. Examination of Subgroups

Subgroup analyses of the primary and key secondary efficacy endpoint (with tests for interaction) will be performed for all of the subgroups specified below. For any subgroup level that does not comprise ≥ 10% of the mITT population, the analyses based on the models will not be performed. Note that for sex at birth and gender identity subgroups that do not comprise ≥ 10% of the mITT population, the number of enrolled participants in each subgroup will be acknowledged in the footnote of the corresponding subgroup table, even if that number is zero. The subgroups of interest are described below.

- Age (18-30, 31-45, 46-65)
- Sex at birth (male, female, unknown or undifferentiated)
- Current gender identity (male, female, trans-male, trans-female, gender non-conforming, different identity, chose to not answer the question)
- Race (Asian – Indian, Asian – Other, Black or African American, White, Mixed Race, Native Hawaiian or Other Pacific Islander, Other)
- Ethnicity (Hispanic or Latino, not Hispanic or Latino, not reported)
- Country (Georgia, Philippines, South Africa, Tanzania, Uganda)
- Region (Africa, Asia, Europe)

PROPRIETARY — © 2021 Rho, Inc.

- Severity of disease (severe, non-severe)
- HIV status (negative, positive)
- Smoking status (never, current, former)
- Alcohol status (never, current, former)

Models used for the subgroup analyses will be the same as those used for the primary and key secondary efficacy endpoint as specified in Sections 8.2.2 and 8.3 with covariates for the subgroup, treatment group by subgroup interaction, and stratification factors (if different from the subgroup).

Heterogeneity of treatment effect across different levels of each subgroup will be evaluated by presenting p-values for the treatment by subgroup interaction. The interaction term tests whether the treatment effect is significantly different across different levels of the subgroup, between males and females, for example. If a subgroup variable has more than two levels then the least severe level (where applicable) will be used as the reference subgroup, and the test will assess whether the treatment effect in the more severe levels differs from that in the reference subgroup.

Forest plots of the treatment effect estimates and corresponding 95% CIs from the subgroup analyses will be provided. Time to stable sputum culture conversion to negative status will be summarised by country using the Kaplan-Meier method and displayed graphically.

Additional subgroup analyses are described for safety analyses in Section 9.

PROPRIETARY — © 2021 Rho, Inc.

9. SAFETY EVALUATION

9.1. Overview of Safety Analysis Methods

To support the reporting for this study, analyses will be performed during the study based on snapshots of the database at key time points of interest as specified in Section 11 (e.g. Week 8 analysis [primary analysis], 52 weeks after EOT, etc.) in addition to the end of study analysis after the final database lock.

The primary safety evaluation will be performed at Week 8 to align with the time point of interest for the primary efficacy endpoint. To align the time period for safety with the time point of interest while also conservatively summarizing all safety at the time of reporting, the following approach will be taken:

- For adverse events (AEs), summary tables will be included that include data through Day x, where x is relevant to the specific time point of interest at the time of report (e.g., Week 8).
- A second summary for key AE tables will be generated using all data at the time of the snapshot.
- An additional summary of key tables will be generated by period (first 4 weeks, second 4 weeks, >8 weeks).
- Exposure adjusted AE rates will be presented.
- Data for the time point of interest can be easily identified in by-visit safety summaries (e.g. vital signs, labs)

Key safety endpoints will also be analyzed separately by subgroups defined by whether a participant met the criteria to stop treatment at Week 15 to explore whether the additional duration of treatment impacted the overall safety and tolerability of the 3 doses of TBAJ876-Pa-L. Summaries will be presented for each subgroup in Section 8.4.4 overall on-trial as well as over time with particular focus on Weeks 8, 15 to 26 and beyond. Key safety endpoints include an overall summary of incidence of treatment emergent adverse events (TEAEs) (including by severity, drug relatedness, seriousness, leading to early study discontinuation), and specific toxicities, post-Day 1 QTcF intervals and changes from Day 1, and incidence of laboratory abnormalities.

For re-treated participants, brief narratives will be written on each participant.

9.2. Extent of Exposure

Descriptive statistics for exposure results (including compliance, Linezolid pauses, Linezolid dose reductions, and full regimen pauses) will be summarised by treatment group and overall, for

PROPRIETARY — © 2021 Rho, Inc.

the safety population. Exposure results will also be summarized by region (Africa, Asia, Europe).

Duration of study drug treatment will be defined as the number of study days on which the participant received study drug. Drug exposure in days will be calculated as follows: (date of last dose – date of first dose + 1 - duration of interruption), where duration of interruption is calculated as: (end date of interruption – start date of interruption + 1). Drug exposure in weeks will be calculated by dividing the exposure in days by 7. The date of last dose is the last available date in the Exposure Log in DDC, if missing then the date of last dose in the disposition treatment page will be used.

The number and percentage of participants who received investigational medicinal product (IMP) will be categorised by the following duration categories for the Safety Population:

- for treatment period 1:
  - <7 weeks (excluded from PP 7 weeks analyses)
  - 7 to <8 weeks (less than allocated)
  - 8 weeks (as expected)
- through treatment period 2:
  - TBAJ876 arms:
    - <15 weeks (among participants who met criteria to stop and stopped treatment at Week 15)
    - 15 weeks (among participants who met criteria to stop and stopped treatment at Week 15)
    - <26 weeks (among participants who did not meet criteria to stop treatment at Week 15)
    - 26 weeks (among participants who did not meet criteria to stop treatment at Week 15)
  - B-Pa-L and HRZE arms:
    - <26 weeks
    - 26 weeks

Duration of study drug exposure will also be summarised using descriptive statistics of the number of days on study drug (n, mean, standard deviation, minimum, median, and maximum).

An exposure listing will be provided, including all available exposure data.

Treatment compliance (%) will be calculated as (# of actual doses / # of planned doses * 100) and summarised using descriptive statistics. The number and percentage of participants in each

PROPRIETARY — © 2021 Rho, Inc.

compliance category (<80%, 80 to <90%, ≥90%) will be presented by treatment group and treatment period. Note that <80% category would represent participants who are excluded from the PP80 population for secondary endpoints. Percentages will be calculated out of the number of participants who were dosed at that dosing period in the Safety Population.

The following definitions will be used for linezolid dose reductions, dose pauses, and discontinuations.

Parameter	Definition
Linezolid dose reduction	A reduction from 600mg to 300mg*
Linezolid dose pause**	
Complete pause	Pause with a recorded pause start and pause end date.
Ongoing Pause	Pause with a start date and no end date and no entry of 'End of Treatment', note: may become discontinuation.
Linezolid discontinuation	Pause followed by an entry of 'End of Treatment'

* Note that any reduction to 0 mg is a linezolid pause.
** A scheduled dispense of TBAJ876/Bedaquiline and Pretomanid must occur to qualify for a linezolid pause.

The following exposure parameters will be summarised according to the general methods:

- The number and percentage of participants with at least one of the following: linezolid pause, discontinuation, or dose reduction.
- For linezolid complete pause, ongoing pause and discontinuations (number and percentage of participants with at least one dose pause, number of dose pauses, total duration of pauses, reason for dose pause). The Linezolid pause information will be retrieved from the DDC IMP Dosing pages indicated by a pause of Linezolid and scheduled dispense of TBAJ876/Bedaquiline and Pretomanid.
- Linezolid dose reduction (number of participants with at least one dose reduction, number of dose reductions, reason for dose reduction).
- Participants experiencing suspected drug related toxicities due to drugs in the regimen other than linezolid can have the full study regimen paused for up to 14 consecutive days in Treatment Period 1 and the full regimen may be interrupted for up to 28 consecutive days in Treatment Period 2. Full regimen pauses will be summarised by number and percentage of participants with at least one full regimen pause, number of full regimen pauses and reason for regimen pause. Information related to these is found on the DDC IMP Dosing pages as pause selected on each dosing page, TBAJ876, Bedaquiline, Pretomanid, and Linezolid. Full regimen discontinuations will also be summarized.

Time to first Linezolid pause, time to Linezolid discontinuation, time to Linezolid first dose reduction, and time to first Linezolid pause and/or dose reduction will also be summarized using the Kaplan-Meier method and displayed graphically. Time to first Linezolid pause and/or dose reduction will also be displayed by region.

PROPRIETARY — © 2021 Rho, Inc.

In addition to the above figures, time to first Linezolid discontinuation, pause and/or dose reduction due to (1) TEAEs [All], (2) Peripheral Neuropathy TEAE and (3) Myelosuppression TEAE will also be summarized using the Kaplan-Meier method and displayed graphically. Time to first Linezolid discontinuation, pause and/or dose reduction due to each of the above TEAEs will also be displayed by region.

A compliance listing will be provided, including all available compliance data.

9.3. Adverse Events (AEs)

9.3.1. Adverse Event Overview

AEs will be assessed and recorded from the time of signing of the informed consent until the Follow-up Week 52 visit (end of main trial) or Follow-up Week 26 after EORT (end of re-treatment). AEs will be mapped to MedDRA PT and SOC using the latest version available at the start of the study. Relationship to study drug will be assessed as not related or related. AEs will be graded according to the Division of AIDS Toxicity Tables [(DAIDS) Table for Grading the Severity of Adult and Pediatric Adverse Events, Corrected Version 2.1 (July 2017), see Protocol Appendix 6]. AEs not covered by DAIDS criteria will be assessed for severity as mild, moderate, severe, or life-threatening, corresponding to toxicity Grades 1 through 4.

AE summaries will summarise only TEAEs, which are defined as AEs which started at or after the first administration of IMP and includes those events started prior to the first administration of IMP but which worsened after the first intake. Adverse events starting after the last administration of IMP and within 28 days after the last dose of IMP will be regarded as treatment emergent. If a partially missing date or time of onset allows the possibility that an AE may be a TEAE it will be assumed that it is a TEAE.

For the primary safety evaluation, only TEAEs which started or worsened during Treatment Period 1 will be considered. If a partially missing date or time of onset allows the possibility that an AE may be attributed to Treatment Period 1 it will be assumed that it is.

AEs will be categorised by TEAE, severity of AE, causal relationship of AE, serious AE (SAE), fatal AE, AE leading to treatment discontinuation, AE leading to treatment interruption, AE leading to study discontinuation, and specific toxicities. An overall summary of the number and percentage of participants in each category will be presented separately for each treatment group. This overall summary of TEAEs by treatment group will also be presented by sex at birth and by gender for the safety population.

The Investigator will provide an assessment of the severity of each AE. Severity will be assessed as missing, mild, moderate, severe, or life-threatening. Please note that the severity collected in DDC is not based on any standardised scale such as Common Terminology Criteria for Adverse Events (CTCAE) but is instead simply the investigator's subjective impression of the intensity of the event. Note: "severe" is a measure of intensity; it is not equivalent to "serious". All AEs will

PROPRIETARY — © 2021 Rho, Inc.

be summarised by maximum severity in terms of the number and percentage of participants experiencing at least 1 AE by SOC and PT, presented separately for each study part by treatment group. Missing severities will be assigned a severity of “severe”.

All AEs will be summarised by the causal relationship (missing, not related (including unlikely), and related (including possibly, probably, and certainly related)) to each study drug (TBAJ876/Bedaquiline, Pretomanid, Linezolid, Bedaquiline, and HRZE/HR presented by treatment group. Participants will be counted under their highest relationship within a SOC and PT. Additionally, TEAEs and SAEs will be summarised by relatedness to each study drug by SOC and PT, presented by treatment group. Missing relationships will be assigned a relationship of “related”.

The following TEAE summary tables will be prepared:

- Overall summary of TEAEs
- TEAEs by SOC and PT
- TEAEs by SOC and PT, by region
- Grade III/IV TEAEs by SOC and PT, by region
- TEAEs by SOC, PT, and DAIDS toxicity grade
- Related TEAEs by SOC and PT (separately for blinded IMP, bedaquiline, pretomanid, linezolid, HRZE, and HR)
- TEAEs leading to treatment discontinuation by SOC and PT (separately for linezolid and IMP)
- TEAEs leading to treatment interruption by SOC and PT (separately for linezolid and IMP)
- TEAEs leading to study discontinuation by SOC and PT
- Specific Toxicities by SOC and PT
- Specific Toxicities by SOC and PT, by region
- Serious TEAEs by SOC and PT
- TEAEs leading to death by SOC and PT

A summary of grade 3 or 4 AEs after 28 days post end of treatment will also be generated.

Events will be sorted by decreasing frequency overall.

PROPRIETARY — © 2021 Rho, Inc.

All AEs recorded in the DDC will be listed for the safety population. A separate listing of AEs will be presented for the following: screen failures, AEs leading to treatment discontinuation, AEs leading to treatment interruption, and AEs leading to study discontinuation.

Duration of AEs will be derived and presented in all listings. It is calculated as AE stop date/time minus AE start date/time. Both start and stop dates need to be present to calculate a duration. If AE start time is missing, it will be treated as 00:00 on the start day; if AE stop time is missing, it will be treated as 23:59 on the stop day.

Exposure-adjusted Adverse Events

Exposure-adjusted AEs will be summarised by SOC and PT with the patient-years, exposure-adjusted event rates, and exposure-adjusted multiple occurrence rates which are defined as follows:

- Number of participants with AE.
- Number of occurrences of unique AEs is defined as the total number of occurrences of unique AEs summed across all participants. For a given participant and PT, an occurrence of a unique AE is determined only by distinct AE start date.
- Patient-years: total exposure up to first AE onset summed across all participants, where a participant's total exposure up to first AE onset is either of the following:
    - Time to first AE, i.e., ([earliest imputed AE start date] – reference start date + 1)/365.25, if the participant had the AE
    - Time in the study if the participant did not have the AE, defined as:
        - (date of last dose – date of first dose + 1)/365.25. Note: the date of last dose is the last available date in the study medication page, if missing then the date of last dose in the disposition treatment page for the main trial will be used.
- Exposure-adjusted rate per patient-year: (number of participants with AE)/patient-years.
- Exposure-adjusted unique occurrences rate per patient-year: (number of occurrences of unique AEs)/patient-years.

Exposure-adjusted AEs will also be analyzed separately by subgroups defined by whether a participant met the criteria to stop treatment at Week 15.

9.4. Deaths, Serious Adverse Events, and Specific Toxicities

The incidence of all-cause mortality will be summarised for each treatment group as described in Section 9.3.

PROPRIETARY — © 2021 Rho, Inc.

All TB-related symptoms that meet SAE criteria will be recorded and reported as a SAE and summarised for each treatment group as described in Section 9.3.

SAEs, adverse events leading to study withdrawal, and fatal AEs will be presented in data listings for participants in the Safety Population.

Specific toxicities will be summarised by SOC and PT, presented by treatment group and include the following:

- SMQs (all narrow and broad PTs): (1) Cardiomyopathy (SMQ), (2) Cardiac arrhythmias (SMQ), (3) Peripheral Neuropathy (SMQ) (overall, and in the first 3 months of treatment), (4) Optic Nerve Disorders (SMQ), (5) Lactic Acidosis (SMQ), (6) Drug related hepatic disorders – comprehensive search (SMQ), and (7) Convulsion, where SMQ refers to Standardised MedDRA Queries .
- MSMQs (Modified SMQ selection of narrow/broad PTs) (1) Rhabdomyolysis/Myopathy (MSMQ), (2) Haematopoietic cytopenias (MSMQ), (3) Acute Pancreatitis (MSMQ, only narrow PTs), and (4) Cardiac Enzymes (MSMQ).

Specific Toxicities will also be summarized by region, sex at birth, and current gender identity for all arms, and also by treatment duration for each of the TBAJ876 treatment arms.

Specific Toxicities will also be summarized by severity and region.

Time to certain specific toxicities (including peripheral neuropathy, optic nerve disorders, and haematopoietic cytopenias) will also be summarized using the Kaplan-Meier method and displayed graphically. This summary will also be conducted by region, sex at birth, and current gender identity.

Logistic regression modelling will also be used to explore predictors of experiencing each specific toxicity (e.g., baseline characteristics).

Specific toxicities will be listed for the safety population.

9.5. Clinical Laboratory Evaluation

Continuous laboratory results and changes from baseline will be summarised descriptively by visit for all clinical laboratory tests (see Protocol Appendix 2), presented by treatment group.

Laboratory results will be summarised for the Safety Population. Grade ≥ 3 laboratory results will be listed.

9.5.1. Analysis of Abnormal Laboratory Value

A list of laboratory tests (haematology, clinical chemistry, and urinalysis) to be included in the analysis is presented in Protocol Section 13.2. Laboratory assessments done by a central laboratory will be summarised in a table by visit and treatment group. Laboratory abnormalities will be graded according to the Division of AIDS Toxicity Tables (DAIDS) Table for Grading

PROPRIETARY — © 2021 Rho, Inc.

the Severity of Adult and Pediatric Adverse Events, Corrected Version 2.1 (July 2017), see Protocol Appendix 6). Laboratory toxicity shifts from baseline to worst post-baseline assessments will be summarised by treatment group. The shift table denominator will be the number of participants with the corresponding baseline grade. The incidence of laboratory results with Grade ≥ 3 will be summarized.

The incidence of liver related laboratory abnormalities will be explored by treatment group using the following categories for worst post-baseline assessments:

- Total number of participants with at least one liver-related abnormality (worst elevation)
- Alanine Aminotransferase (ALT) or Aspartate Aminotransferase (AST) >3 x upper limit of normal (ULN) and ≤5 x ULN
- ALT or AST >5 x ULN and ≤8 x ULN
- ALT or AST >8 x ULN
- Bilirubin > 2 x ULN
- Alkaline Phosphatase (ALP) >2 x ULN
- ALT or AST >3 x ULN and total bilirubin >2 x ULN
- ALT or AST >3 x ULN and total bilirubin >2 x ULN and ALP <2 x ULN (potential Hy's law case)

Laboratory liver-related abnormalities will also be summarized by region.

Graphs of the mean change from baseline by treatment for AST, ALT, ALP, total bilirubin, hemoglobin, ANC, platelets, and eosinophils will be produced by visit.

Liver enzyme profile plots including normalised AST, ALT, ALP, and total bilirubin values by study day will be produced individually for participants with at least one liver-related abnormality. Interruptions to study treatment will be identified in the plots.

The incidence of liver related laboratory abnormalities will also be explored by treatment group using the categories which correspond to different DAIDS grades for worst post-baseline assessments:

- Total number of participants with at least one liver-related abnormality (worst elevation) per DAIDS Grade 2 or higher
- ALT or AST ≥2.5 x ULN and <5 x ULN
- ALT or AST ≥5 x ULN and <10 x ULN
- ALT or AST ≥10 x ULN

PROPRIETARY — © 2021 Rho, Inc.

- Bilirubin ≥1.6 x ULN and <2.6 x ULN
- Bilirubin ≥2.6 x ULN and <5 x ULN
- Bilirubin ≥5 x ULN
- ALP ≥2.5 x ULN and <5 x ULN
- ALP ≥5 x ULN and <10 x ULN
- ALP ≥10 x ULN

An evaluation of drug-induced serious hepatotoxicity (eDISH) scatter plot will display the maximum TBL ratio of value to ULN on the y-axis versus the maximum ALT ratio of value to ULN on the x-axis, where the maxima are not necessarily concurrent, for the Safety Population. Both axes will be on the log10 scale. Ratios < 0.1 x ULN will be set to 0.1. Sample sizes in the legend will represent subjects with paired ratios. A horizontal reference line will be placed at 2 x ULN, and a vertical reference line will be placed at 3 x ULN. The lower left quadrant will be labeled “Normal Range”, the upper left quadrant will be labeled “Hyperbilirubinemia”, the lower right quadrant will be labeled “Temple’s Corollary”, and the upper right quadrant will be labeled “Possible Hy’s Law Range.”

9.6. Electrocardiograms (ECGs)

The central cardiologist’s assessment will be communicated to the site after centralised reading and will include the following measures: heart rate, PR interval, RR interval, the corrected QT interval by Fridericia (QTcF), and QRS.

Electrocardiogram (ECG) results and changes from baseline (defined as the average of Screening and Day 1 values) will be summarised descriptively by visit and treatment group. ECG interpretations, which include clinical significance, will also be summarised by visit and treatment group. The triplicate mean will be used for descriptive statistics for each applicable post-baseline visit and time point.

QTcF intervals will be classified into the following categories:

- QTcF <= 450 msec
- 450 msec < QTcF <= 480 msec
- 480 msec < QTcF <= 500 msec
- QTcF >500 msec

QTcF changes from Day 1 will be classified into the following categories:

- increase < 30 msec,
- 30 <= msec and <60 msec, and

PROPRIETARY — © 2021 Rho, Inc.

- increase >60 msec.

Frequency counts will be used to summarise the number of participants at each time point according to the above categories.

ECG results and changes from baseline will be summarised descriptively by time point and for worst post-baseline assessment, presented by treatment group for the Safety Population.

Participants with any abnormal QTcF values that resulted in early withdrawal of treatment will have their QTcF intervals presented in a figure vs time.

ECG results and associated interpretations/findings will be listed for the Safety Population.

9.7. Visual Acuity

Visual acuity will be listed by visit for participants in the Safety Population.

9.8. Peripheral Neuropathy

Descriptive statistics of neuropathy data derived from Brief Peripheral Neuropathy Screen will be summarised and listed for the Safety Population by treatment group and also by region. Categorical data for observed signs and symptoms of neuropathy will be summarised in frequency tables, including changes in signs and symptoms from baseline.

9.9. Vital Signs, Physical Findings, and Other Observations Related to Safety

9.9.1. Vital Signs and Physical Examination

Systolic and diastolic blood pressure (mmHg) are to be measured supine (after 5 minutes of rest) using an appropriately sized cuff and using the same type of sphygmomanometer by the same observer, if possible, at each relevant visit.

- Weight will be recorded in kilograms.
- Respiratory rate will be recorded as breaths per minute.
- Heart rate will be recorded as beats per minute.
- Temperature will be measured using a digital temperature scanner.

Vital sign results and changes from baseline will be summarised descriptively by visit, presented by treatment group for the Safety Population.

Abnormal vital sign assessment results will be identified, and the number and percentage of participants with at least one post-baseline abnormality will be summarised.

PROPRIETARY — © 2021 Rho, Inc.

In addition, a shift table from baseline to worst post-baseline category result will be presented for systolic blood pressure, diastolic blood pressure, and heart rate.

Abnormal vital sign results will be listed for the Safety Population.

Abnormal physical examination findings will be listed for the Safety Population.

9.9.2. Concomitant Medications

Concomitant medications taken 30 days prior to screening until the end of the trial (52 weeks after the EOT) should be collected in the case report form and medical records. Prior and concomitant medications will be coded using the latest World Health Organization Drug Dictionary Version.

All medications will be summarised per Anatomical Therapeutic Chemical (ATC) level 2 and level 4 code. Medications that cannot be assigned a level 2 or level 4 code will be identified in the table as missing the coding level.

A prior medication is defined as any medication taken prior to the date and time of the first dose of study drug. A concomitant medication is defined as any medication taken on or after the date and time of the first dose of study drug through the date and time of the last dose of study drug. A medication that starts before the first dose of study drug and ends after the first dose will be counted as both a prior and concomitant medication.

All prior and concomitant medications will be summarised for the Safety Population by treatment group and overall. Participants receiving the same medication more than once will be counted only once for a particular medication class and medication.

Prior and concomitant medication data will also be presented in a data listing for participants in the Safety Population.

Partial dates will be imputed following the same algorithm as for TEAEs, i.e., a partially missing start or end date allows the possibility that a medication may be a concomitant it will be assumed that it is concomitant.

For an entirely missing start date (i.e., day, month, and year are missing), the start date will be set to the start date of administration of study drug unless the stop date is prior to the start date of administration of study drug, in which case the start date will be set to the stop date.

9.9.3. Concomitant Procedures

Concomitant procedures taken 30 days prior to screening until the end of the trial (52 weeks after the EOT) should be collected in the case report form and medical records. Concomitant procedures will be coded using the latest MedDRA version.

Concomitant is defined as described for medications in Section 9.9.2.

PROPRIETARY — © 2021 Rho, Inc.

Procedures will be summarised by SOC and PT. All prior and concomitant procedures will be summarised for the Safety Population by treatment group and overall. Participants receiving the same procedure more than once will be counted only once for a particular SOC and PT.

Concomitant procedure data will also be presented in a data listing for participants in the Safety Population.

Partial dates will be imputed following the same algorithm as for TEAEs, i.e., a partially missing start or end date allows the possibility that a medication may be a concomitant it will be assumed that it is concomitant.

For an entirely missing start date (i.e., day, month, and year are missing), the start date will be set to the start date of administration of study drug unless the stop date is prior to the start date of administration of study drug, in which case the start date will be set to the stop date.

PROPRIETARY — © 2021 Rho, Inc.

10. PHARMACOKINETIC EVALUATION

10.1. Pharmacokinetic Endpoints

For each analyte and each scheduled sampling time, the plasma concentration will be summarised by descriptive statistics including the mean, SD, CV, median, minimum, and maximum.

Plasma concentrations by actual time point will be summarised graphically. Mean concentrations (+/- SD) by nominal time point will be presented by treatment group.

Trough concentrations of dolutegravir and tenofovir will be calculated and summarized by descriptive statistics for participants living with HIV. Box plots of trough concentrations of dolutegravir and tenofovir will be presented by time post-dose.

MIC at baseline and week 8 for bedaquiline, pretomanid and TBAJ876 will be summarized by descriptive statistics including change from baseline.

10.2. Pharmacokinetic Methods

10.2.1. Pharmacokinetic Data Conventions

Data Below the Limit of Quantification and Outlying Data:

Concentrations that are below the limit of quantification (BLQ) will be presented as "BLQ" in listings. Any table or listing that contains concentration data will include the lower limit of quantification (LLOQ) for the applicable assay as a footnote.

For calculation of summary statistics for concentration data, concentrations that are BLQ will be treated as zero. Individual BLQ concentrations may alternatively be set to missing if deemed appropriate (e.g., BLQ result is implausible based on the totality of available data). If any values are set to missing for concentration analyses, this will be described in the CSR along with the rationale for doing so. These values will be reported as "BLQ" in individual- participant summaries (including listings) with a flag to denote the rationale for excluding in calculation of summary statistics.

If ≥ 33% of the available concentration values at a given timepoint (or interval) are BLQ or missing, only the number of samples, minimum, and maximum will be included in the summary tables. All other descriptive statistics parameters will be reported as "NC" (not calculable).

Before estimation of PK parameters, concentrations will be examined visually, and clearly outlying values will be excluded and documented as such. Non-outlying BLQ concentrations will be set to 0 for calculation of PK parameters.

PROPRIETARY — © 2021 Rho, Inc.

Missing Data:

For participants who have missing data (e.g., due to premature discontinuation, missed sample(s), etc.), all available data will be included for the key clinical pharmacology analyses.

In the following instances missing concentration data may be imputed for purposes of PK parameter analysis and generation of summary statistics, without additional justification:

- Missing pre-dose values may be imputed to the minimum observed concentration (Cmin) or the concentration at the end of the dosing interval.
- Missing values at the end of the dosing interval may be imputed to the pre-dose value.

Results that are BLQ are not considered missing data.

Rounding:

Individual-participant plasma, urine, and saliva concentration data and PD data will be reported in listings to the same number of decimal places (DP) or significant figures (SF) as in the bioanalytical report.

Individual-participant PK parameters for plasma will be rounded to 3 SF.

For summary and inferential statistics, the following conventions will be applied:

- Number of participants/samples (n) will be reported as a whole number
- Minimum and maximum values will be reported to the same number of DP or SF as the individual-participant data
- All other summary and inferential statistics will be reported to 3 SF

10.2.2. Pharmacokinetic Endpoint Estimation

Concentrations from intensively sampled profiles and, as feasible, from sparse sampling will be used to compute summary metrics of exposure defined in Table 3 over a dosing interval for TBAJ876 and its M3 metabolite, bedaquiline and its M2 metabolite, pretomanid, and linezolid. Pre-dose values will be used for 24-hour post-dose values for participants with sparse sampling for computing summary metrics of exposure.

Plasma PK parameters for each participant will be estimated over the sampling interval by non-compartmental analysis (NCA) using Phoenix® WinNonlin® (Certara, New Jersey, USA) version 8.3 or higher. AUCs will be calculated using the linear-log trapezoidal summation method in WinNonlin, also known as “linear-up log-down” trapezoidal summation.

PROPRIETARY — © 2021 Rho, Inc.

Table 3: Parameters for Pharmacokinetic Analysis

Pharmacokinetic Parameter	Units	Definition	Phoenix WinNonlin Parameter Output
C_{max}	ng/mL	Observed maximum plasma concentration, obtained directly from the observed versus time concentration data	Cmax
t_{max}	h	Time of Cmax	Tmax
AUC_{tau}	ng·h/mL	Area under the PK plasma concentration time (t) curve over the dosing interval	AUC_TAU
C_{min}	ng/mL	Observed minimum plasma concentration, obtained directly from the observed versus time concentration data	Cmin
t_{min}	h	Time of Cmin	Tmin

Additional parameters will be reported for TBAJ876:

- SAUC = $AUC_{tau,TBAJ876}$ + $AUC_{tau,M3}$: Sum of the areas under the curve for TBAJ876 and M3. This metric will be used for exposure-response analyses of safety endpoints.
- SAM = $AUC_{tau,TBAJ876}/1$ + $AUC_{tau,M3}/3$: Sum of the areas under the curve for TBAJ876 and its M3 metabolite divided by representative MIC values, 1 ng/mL and 3 ng/mL, respectively. These MIC values have been determined for the H37Rv strain of Mtb. Other values may be used if judged more appropriate at the time of analysis. This metric will be used for exposure-response analyses of efficacy endpoints.

10.2.3. Statistical Analysis of Pharmacokinetic Endpoints

Summary statistics for each summary metric of exposure will be reported by treatment group overall as well as for the subgroups identified in Section 8.4.4 to assess the relationship between exposure and efficacy outcomes across all analytes. The geometric mean, CI of geometric mean, and geometric CV will be included for C_{max}, AUC_{tau}, and C_{min}.

Box plots of C_{max} and AUC_{tau} for each analyte, and SAUC and SAM for TBAJ876, will be presented by applicable treatment group.

10.2.4. Analyses of Efficacy versus Exposure

Further analyses will explore the relationship between the primary endpoint, time to stable sputum culture over 8 weeks, and exposure of TBAJ876. The value of SAM at Day 15 (SAM_{D15}) will be used. If a participant is missing a value of SAM at Day 15 but has one at Week 8 (SAM_{W8}), then

PROPRIETARY — © 2021 Rho, Inc.

SAM_{D15} will be imputed from SAM_{W8} by multiplying SAM_{W8} by the median value of the ratio of SAM_{D15} to SAM_{W8} for all participants in the same treatment group with both values. Participants missing both SAM_{D15} and SAM_{W8} will be excluded. Estimates of hazard ratios at the 10th, 25th, 75th, and 90th percentiles (or other meaningful values) of SAM_{D15} relative to the hazard at the median (or other meaningful value) of SAM_{D15} will be calculated using a stratified Cox proportional hazards model with the individual centered, log-transformed SAM_{D15} and baseline TTP as predictor variables and the stratification factors country and severity of disease. Corresponding 2-sided 95% CIs will also be presented.

To examine the assumption of proportional hazards in the Cox regression model, the 'assess' statement in SAS proc phreg with the 'PH' and 'RESAMPLE' options will be used to generate various graphical plots and a Kolmogorov-type supremum test. If non-proportional hazards are detected for a predictor variable, Schoenfeld's residuals will be further examined to guide the addition of a time-dependent interaction to the model. Other statistical tests and graphical methods may also be used to evaluate the proportional hazards assumption.

Using a similar approach, logistic regression modelling will be used to explore whether SAM is a predictor of achieving a favourable outcome at 26 weeks after the EOT visit, adjusting for baseline TTP and stratification factors. For this purpose, a time-average of SAM_{D15} and SAM_{W8} will be used. Considering SAM_{D15} to be at Week 2 and assuming SAM is zero at time zero, an area-under-SAM through Week 8 may be computed via the trapezoidal rule as

$$0.5x(0 + SAM_{D15})x(2 \text{ weeks}) + (0.5)x(SAM_{D15} + SAM_{W8})x(6 \text{ weeks}).$$

Dividing by 8 weeks yields a time-average. Thus,

$$SAM_{avg} = (4xSAM_{D15} + 3xSAM_{W8})/8.$$

Participants with both SAM_{D15} and SAM_{W8} missing and participants who withdrew before Week 8 will be excluded. Otherwise, if SAM_{D15} is missing, it will be imputed as above; and if SAM_{W8} is missing it will be imputed from SAM_{D15} via the median of ratios of SAM_{W8} to SAM_{D15} within the same treatment group.

Using a similar approach, logistic regression modelling will be used to explore whether centered, log-transformed SAM_{avg} is a predictor for relapse at 26 weeks after the EOT visit.

10.2.5. Analyses of Safety versus Exposure

Similar analyses of TBAJ876 exposure versus response via logistic regression modelling will be performed, as feasible, for the following safety outcomes through eight weeks of treatment:

- Participants experiencing AEs leading to discontinuation (Yes/No)
- Participants experiencing a Specific Toxicity (Yes/No). This analysis will be conducted for each specific toxicity listed in Section 9.4.

PROPRIETARY — © 2021 Rho, Inc.

- Participants with at least one Treatment Emergent AST or ALT > 3x ULN (Yes/No).
- Participants with a QTcF interval > 450 msec and/or an increase in QTcF from baseline of >= 30 msec.

For these analyses, centered, log-transformed $SAUC_{D15}$, imputed from $SAUC_{W8}$ if necessary, as described above for SAM_{D15}, will be used as the predictor variable.

10.2.6. Additional Analyses

A separate population pharmacokinetic (PopPK) analysis and additional exposure-response analyses may be performed, if feasible, and reported elsewhere.

PK/QT analyses may be conducted on data from this study and will be described in a separate analysis plan.

PROPRIETARY — © 2021 Rho, Inc.

11. INTERIM ANALYSES AND DATA MONITORING

There will be 1 planned unblinded interim analysis which will contain results by treatment group in aggregate and will include the primary analysis. This will occur after all participants have completed 8 weeks of treatment including after any missed doses have been made up at the end of Treatment Period 1.

In addition, at the following times:

- After all participants have completed the 52-week follow-up period after treatment
- At end of study (final database lock), after treatment failure participants have completed the 26-week follow-up after re-treatment

Additional analyses may be performed during trial conduct to support organizational decision making or interactions with regulatory authorities. In this circumstance, a limited number of individuals will have access to individual treatment assignments. The sites, participants, and individuals involved in the day-to-day trial conduct will remain blinded to individual treatment information, until the end of the trial.

Additional details regarding data handling and unblinding during each interim analysis will be provided in the study's Unblinding Plan.

PROPRIETARY — © 2021 Rho, Inc.

12. APPENDIX

12.1. Study Flow Chart

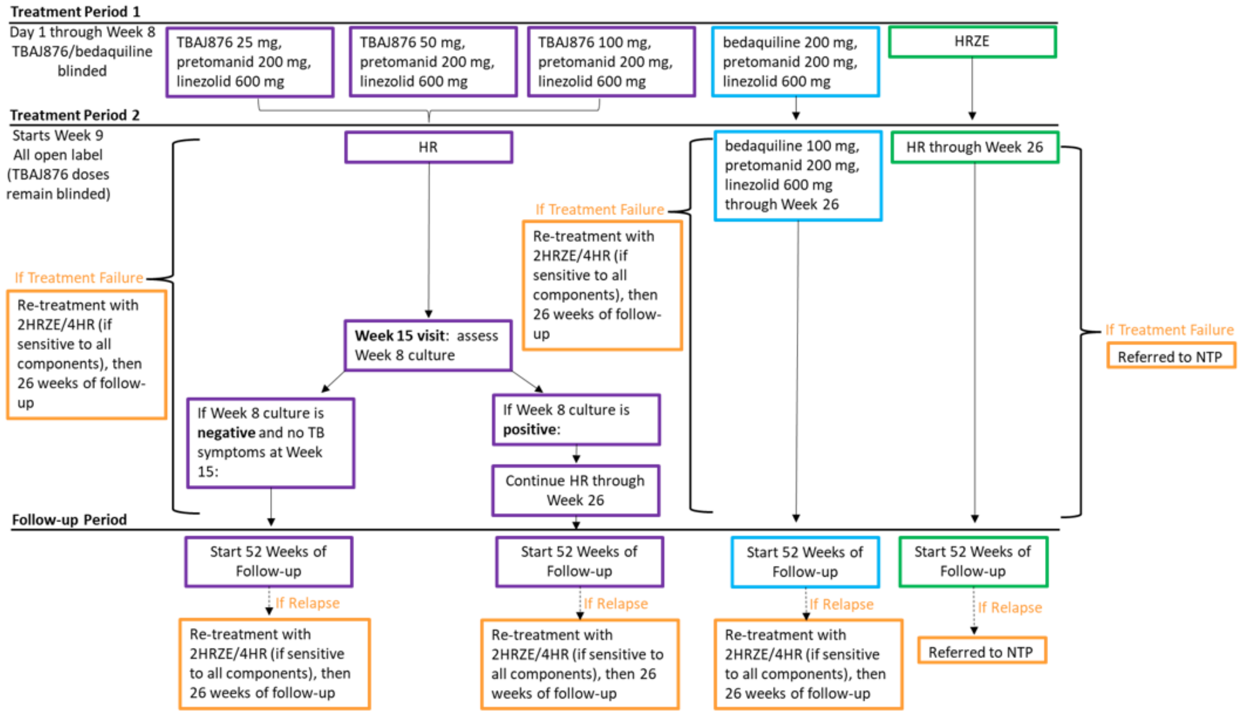

At the Week 15 visit, the Week 8 culture and presence of TB-related symptoms will be assessed for participants who received TBAJ876-Pa-L. If the Week 8 culture is negative, and the participant has no TB-related symptoms by Week 15, the participant can complete treatment at Week 15. Participants with symptoms that have a more likely alternative explanation are eligible to complete treatment at Week 15. If the Week 8 culture is MTB positive and/or the participant has TB symptoms, the participant will continue treatment through Week 26.

PROPRIETARY — © 2021 Rho, Inc.

12.2. Schedule of Events

Period	Screening	Treatment Period 1												Treatment Period 2						Post EOT Follow-up						Early Trial Discontinuation Visit	Protocol Section
Time of Visit	Up to 11 days before 1st IMP dose	Day 1	Day 4	Day 8	Day 11	Day 15	Week 3	Week 4	Week 5	Week 6	Week 7	Week 8	[1] EOT Make-up Period 1	Week 9	Week 12	[2] Week 15 or EOT	[3] Week 20	[3] Week 26 EOT	[4] EOT Make-up Period 2	FU Week 4	FU Week 8	FU Week 16	FU Week 26	FU Week 38	FU Week 52		
Visit Window	-11 to -1 days		±1 day				±3 days							±5 days						±14 days							
Informed Consent	X																										13.1.3
Demography	X																										
Medical History	X																										11.3.9
Urine Drug Screen	X																										13.2
Urine Pregnancy Test	X	X						X		X		X			X			X				X			X	X	11.3.5
Urinalysis	X																										11.3.4
Exploratory Urine Sample		X						X				X				X		X									11.6
Inclusion/Exclusion	X	X																									8
Randomisation		X																									9.3
Karnofsky Assessment	X																										13.8
HIV Screening	X																										11.3.6
CD4 Count, Viral Load	X							X				X						X							X	X	11.3.6
HBsAg, Anti-Hep C Ab	X																										
Chest X-ray	X														X			X					X		X	X	11.3.7
Spot Sputum	X	X	X	X	X	X	X	X	X	X	X	X	X	X	X	X	X	X	X	X	X	X	X	X	X	X	11.2.1
Visual Acuity	X					X		X		X		X			X	X	X	X		X		X				X	11.3.8
Vital Signs	X	X	X	X	X	X	X	X	X	X	X	X	X	X	X	X	X	X	X	X	X	X	X	X	X	X	11.3.2
Single 12-lead ECG	X																										11.3.3
Triple 12-lead ECG		X				X						X	X		X			X	X							X	11.3.3

Period	Screening	Treatment Period 1												Treatment Period 2						Post EOT Follow-up						Early Trial Discontinuation Visit	Protocol Section
Time of Visit	Up to 11 days before 1st IMP dose	Day 1	Day 4	Day 8	Day 11	Day 15	Week 3	Week 4	Week 5	Week 6	Week 7	Week 8	[1] EOT Make-up Period 1	Week 9	Week 12	[2] Week 15 or EOT	[3] Week 20	[3] Week 26 EOT	[4] EOT Make-up Period 2	FU Week 4	FU Week 8	FU Week 16	FU Week 26	FU Week 38	FU Week 52		
Visit Window	-11 to -1 days		±1 day				±3 days							±5 days						±14 days							
[5] PK Sampling (Trial Drugs)		X		X		X	X	X	X	X	X	X	X		X			X	X							[6] X	11.5.1
[7] PK Sampling (ARV)		X		X				X				X															11.5.2
PK Sampling (24 hour)						X																					11.5.1.2
[5] Pharmacogenetic Sample		X																									12.6.1
Full Physical Exam	X	X														X		X	X								11.3.1
Height	X																										11.3.1
Cardiovascular Exam			X	X	X	X	X	X	X	X	X	X	X	X	X		X			X	X	X				X	11.3.1
Symptom Directed Exam			X	X	X	X	X	X	X	X	X	X	X	X	X		X			X	X	X	X	X	X	X	11.3.1
TB Symptoms Profile		X						X				X	X			X		X	X				X		X	X	11.2.2
Brief Peripheral Neuropathy Assessment	X					X		X		X		X	X		X	X	X	X	X			X				X	0
Laboratory Safety Test	X	X		X		X	X	X	X	X	X	X	X	X	X	X	X	X	X							X	11.3.4
Exploratory Blood Sample		X						X				X				X		X									11.6
IMP Administration, Adherence		X	X	X	X	X	X	X	X	X	X	X	X	X	X	X	X	X	X							[5] X	9.1
Quality of Life Questionnaire		X														X									X	X	11.3.10
Concomitant Medications		X	X	X	X	X	X	X	X	X	X	X	X	X	X	X	X	X	X	X	X	X	X	X	X	X	9.7
Adverse Events	X	X	X	X	X	X	X	X	X	X	X	X	X	X	X	X	X	X	X	X	X	X	X	X	X	X	11.4

1. The EOT Make-up Period 1 visit will only be performed if the participant has missed 4 to 14 cumulative IMP doses. If less than 4 doses were missed, the participant is also required to make up those missed doses by the Week 8 visit, but there is no need to perform an additional visit prior to the transition to Treatment Period 2. See Section 9.5.2.
2. Week 15 can either be the EOT visit for participants who complete treatment at this visit in the TBAJ876-Pa-L arms or a regular visit for participants who continue treatment until Week 26. See Section 7.1 for details.
3. Visit only applicable to participants who complete treatment at Week 26.
4. The EOT Make-up Period 2 visit will only be performed if the participant has missed 6 to 28 cumulative IMP doses (during Treatment Period 2). Making up missed doses is required for participants who complete treatment at Week 15 or 26; however, the additional EOT visit is not required prior to proceeding to the Follow-up Period if less than 6 doses were missed. See Section 9.5.2.
5. Excluding participants randomised to 2HRZE/4HR.
6. IMP adherence will be assessed and PK samples will be taken only if the Early Trial Discontinuation Visit is done during either of the treatment periods and not during follow up.
7. Applies to all treatment arms; only for participants living with HIV and on ARV.

Note: all section numbers refer to the protocol.

PROPRIETARY — © 2021 Rho, Inc.

12.3. Visit Windowing

Period	Visit	Assessment Day	Target Window	Analysis Window[a]
Screening	Screening – Up to 11 days before 1st IMP dose	-40 to -30	N/A	N/A - Nominal DDC visit
Treatment Period 1	Day 1	1	1	N/A - Nominal DDC visit
	Day 4	4	3-5 (+/- 1)	2-6
	Day 8	8	7-9 (+/- 1)	7-9
	Day 11	11	10-12 (+/- 1)	10-12
	Day 15	15	14-16 (+/- 1)	14-17
	Week 3	21	18-24 (+/- 3)	18-24
	Week 4	28	25-31 (+/- 3)	25-31
	Week 5	35	32-38 (+/- 3)	32-38
	Week 6	42	39-45 (+/- 3)	39-45
	Week 7	49	46-52 (+/- 3)	46-52
	Week 8	56	53-59 (+/- 3)	53-59
	EOT Make-up Period 1	N/A	N/A	N/A - Nominal DDC visit
Treatment Period 2	Week 9*	63	58-68 (+/- 5)	60-73
	Week 12*	84	79-89 (+/- 5)	74-94
	Week 15 or EOT*	105	100-110 (+/- 5)	95-122 or N/A – Nominal DDC visit

PROPRIETARY — © 2021 Rho, Inc.

	Week 20*	140	135-145 (+/- 5)	123-161
	Week 26 EOT*	182	177-187 (+/- 5)	162-187
	EOT Make-up Period 2	N/A	N/A	N/A – Nominal DDC visit
Post EOT Follow-up	FU Week 4	EOT + 28	EOT + 14-42 (+/- 14)	EOT + 14-42
	FU Week 8	EOT + 56	EOT + 42-70 (+/- 14)	EOT + 43-84
	FU Week 16	EOT + 112	EOT + 98-126 (+/- 14)	EOT + 85-147
	FU Week 26	EOT + 182	EOT + 168-196 (+/- 14)	EOT + 148-224
	FU Week 38	EOT + 266	EOT + 252-280 (+/- 14)	EOT + 225-315
	FU Week 52	EOT + 364	EOT + 350-378 (+/- 14)	EOT + 316-378
	Early Trial Discontinuation Visit	N/A	N/A	N/A – Nominal DDC visit

* If participant completes an EOT Make-up Period 1 visit, add the following number of study days to the target date and windows: (EOT Make-up Period 1 visit date – Week 8 visit date)

PROPRIETARY — © 2021 Rho, Inc.

12.4. Definitions

12.4.1. Inability to produce sputum

In general, inability to produce sputum is treated as being equivalent to having a negative (favourable) culture result. This includes the rare situation where a patient never achieves culture negative status due to inability to produce sputum but completes follow-up without clinical or microbiological evidence of relapse. Such a patient will be considered to have a favourable outcome.

12.4.2. Isolated positive cultures

It is known that occasionally patients produce sputum samples that are "isolated positives", that is a positive culture preceded by a series of negative cultures and followed thereafter by at least 2 negative cultures without an intervening positive result. This phenomenon may be the result of a sealed cavity breaking down or laboratory contamination and does not in itself signify that the patient is relapsing. In the event of a single positive culture result occurring in a patient who has previously been classified as having culture negative status (in the absence of any retreatment), the patient will not be classified as a recurrence unless a second positive culture result is obtained at a separate visit (at least 7 days apart) without an intervening negative culture or unless the patient is lost to follow up or completes the study (and is unable to be brought back) before two negative cultures are obtained..

To expand a bit, most of the experience with isolated positives has been with liquid culture. Because liquid culture is more sensitive, it is possible that more than one isolated positive may occasionally occur. Therefore, the clinical condition of the patient will also be considered when deciding whether re-treatment is indicated and in determining the outcome.

12.4.3. Positive culture

Positive culture refers to the culture being positive for *Mycobacterium tuberculosis* (MTB). False positive or contaminated sputum cultures, without speciation data confirming presence of MTB, will be treated as missing. Specimens classified as non-tuberculous mycobacteria (NTM) and negative for MTB will be treated as contaminated.

Ideally, 2 sputum samples are collected at each visit at the research site under the coaching and observation of the trial staff (spot samples). Every effort is to be made to collect sputum samples. However, the inability to produce sputum under experienced staff coaching is considered equivalent to having a negative culture (favourable) result.

The culture result for a given visit is established using all samples obtained for that visit. A positive culture takes precedence over a negative or contaminated culture at the same visit and a negative culture takes precedence over a contaminated culture at the same visit.

PROPRIETARY — © 2021 Rho, Inc.

12.4.4. Derived MGIT results per visit

Derived sample Culture 1 (Visit X)	Derived Sample Culture 2 (Visit X)	Final Derived Result for Visit X
Positive	Missing/Negative/Contaminated	Positive
Negative	Missing/Contaminated	Negative
Missing/Contaminated	Missing/Contaminated	Missing

12.4.5. Interpretation of Relapse/Reinfection using Whole Genome Sequencing (WGS)

The purpose of the WGS analysis is to determine if the two *M. tuberculosis* strains from a given participant (positive culture at baseline and at or after the end of treatment) can be considered the same (treatment failure/bacteriologic failure or relapse/bacteriological relapse), or different (reinfection/bacteriological reinfection).

To do this, WGS of the two *M. tuberculosis* strains are compared, the number of SNPs/variants determined, and the criteria outlined below followed.

These cut offs have been determined from previously published reports from REMoxTB and RIFAQUIN trials and show a clear genetic distinction between relapse and reinfection cases of *M. tuberculosis* infection.

≤12 SNPs different = Relapse

≥100 SNPs different = Reinfection

>12 and <100 SNPs different = Indeterminate

Indeterminate results will be reviewed on a case-by-case basis and are likely to be rare.

Additional sequence analysis may be performed and/or additional samples may need to be tested.

Any additional investigations will be documented on the 'WGS Indeterminate Proforma' which also includes the final conclusion of 'relapse' or reinfection' based on this further review.

A participant will be considered a relapse unless there is sufficient evidence to support a classification of reinfection.

PROPRIETARY — © 2021 Rho, Inc.
