# Supplementary material for: Protocol for a phase 2, partially blinded, randomized trial assessing the safety and efficacy of sorfequiline or bedaquiline in combination with pretomanid and linezolid in adult participants with newly diagnosed, drug-sensitive, smear-positive pulmonary tuberculosis (NC-009)
Source: Trials. 2026 Jan 6;27:102. doi: 10.1186/s13063-025-09413-5 (PMC12869905; doi:10.1186/s13063-025-09413-5)
Supplement: Supplementary file 4 — Additional file 4. NC009 Data Safety Monitoring Committee Charter. [file 13063_2025_9413_MOESM4_ESM.pdf]

Data Safety Monitoring Committee Charter

Version 1.0 Date 27 September 2023

CONFIDENTIAL

Protocol Title: A phase 2, partially-blinded, randomised trial assessing the safety and efficacy of TBAJ876 or bedaquiline, in combination with pretomanid and linezolid in adult participants with newly diagnosed, drug-sensitive, smear-positive pulmonary tuberculosis

Protocol Number: NC-009

Author name: Leandra Lombard
Author position: Clinical Project Manager
Author signature and date:
DocuSigned by:
Leandra Lombard
Signer Name: Leandra Lombard
Signing Reason: I am the author of this document
Signing Time: September 27, 2023 | 4:34 AM PDT
4937F95DE43E460686F675BCCD798AA0

Approver name: Morounfolu Olugbosi, MD
Co-Author position: Study Physician
Co-Author signature and date:
DocuSigned by:
Morounfolu Olugbosi
Signer Name: Morounfolu Olugbosi
Signing Reason: I approve this document
Signing Time: September 27, 2023 | 7:43 AM EDT
3DABF6C3410E4B8F85E1D6E511FA5A5F

Approver name: Prof. Nick Paton
Approver position: Data Safety Monitoring Committee Chairperson
Approver signature and date:
DocuSigned by:
Nick Paton
Signer Name: Nick Paton
Signing Reason: I approve this document
Signing Time: September 28, 2023 | 5:34 AM PDT
DAC57715F6BD41C3A78486A47F80F306

Revision History

Version Number:	Author:	Date	Reason for Revision:
1.0	Leandra Lombard	27 September 2023	Initial

Table of Contents

Purpose 4

Protocol Treatment & Endpoints 4

Committee Membership 9

Roles & Responsibilities 11

Confidentiality 11

Meeting Format & Frequency 11

Meeting Documentation 13

DSMC Recommendation Letter & Distribution 14

Appendix 1: DSMC Member Signature Page 16

Appendix 2: Template NC-009 DSMC Recommendation Letter 17

Purpose

The Data Safety Monitoring Committee (DSMC) charter provides a detailed summary of the standard operating procedures regarding the roles and functions of the DSMC. This includes the roles and responsibilities of the DSMC, DSMC membership, meeting timelines and frequency, data to be reviewed, methods of providing data to the DSMC for review, safety data evaluation and reporting from the DSMC.

The DSMC Charter should be finalised before the first participant is randomised to the study.

Protocol Treatment & Endpoints

Treatment Groups and Duration

**Day 1 to Week 8**<br>*QD for 8 weeks*			**Week 9 to Week 15**<br>*QD for 7 weeks*			**Week 15 to Week 26**<br>***if MGIT Positive and/or with TB symptoms***		
Blinding	Regimen	Number of tablets	Blinding	Regimen	Number of tablets	Blinding	Regimen	Number of tablets
☐ Open label<br>☒ Blinded	TBAJ876 **25 mg** Pa-L	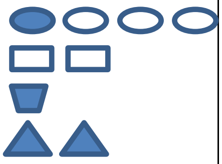	☒ Open label<br>☐ Blinded	HR	Up to 5 tablets *(per weight band)*	☒ Open label<br>☐ Blinded	HR	Up to 5 tablets *(per weight band)*
	TBAJ876 **50 mg** Pa-L	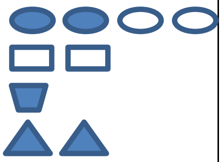						
	TBAJ876 **100 mg** Pa-L	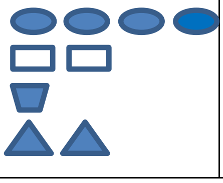						

B-Pa-L Regimen

**Day 1 to Week 8**<br>*QD for 8 weeks*			**Week 9 to Week 26**<br>*QD for 18 weeks*		
Blinding	Regimen	Number of tablets	Blinding	Regimen	Number of tablets
☐ Open label<br>☒ Blinded	B-Pa-L	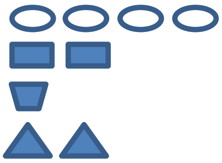	☒ Open label<br>☐ Blinded	B-Pa-L	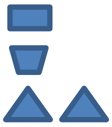

2HRZE/4HR Regimen

**Day 1 to Week 8** *QD for 8 weeks*			**Week 9 to Week 26** *QD for 18 weeks*		
Blinding	Regimen	Number of tablets	Blinding	Regimen	Number of tablets
☒ Open label<br>☐ Blinded	HRZE	Up to 5 tablets<br>*(per weight band)*	☒ Open label<br>☐ Blinded	HR	Up to 5 tablets<br>*(per weight band)*

Key and Unit dose strength/s

- TBAJ876 25 mg tablet
- TBAJ876 placebo
- Pretomanid 200 mg tablet
- Bedaquiline 100 mg tablet
- Bedaquiline placebo
- Linezolid 300 mg tablet (open label)

TB Alliance NC009

Study Schematic

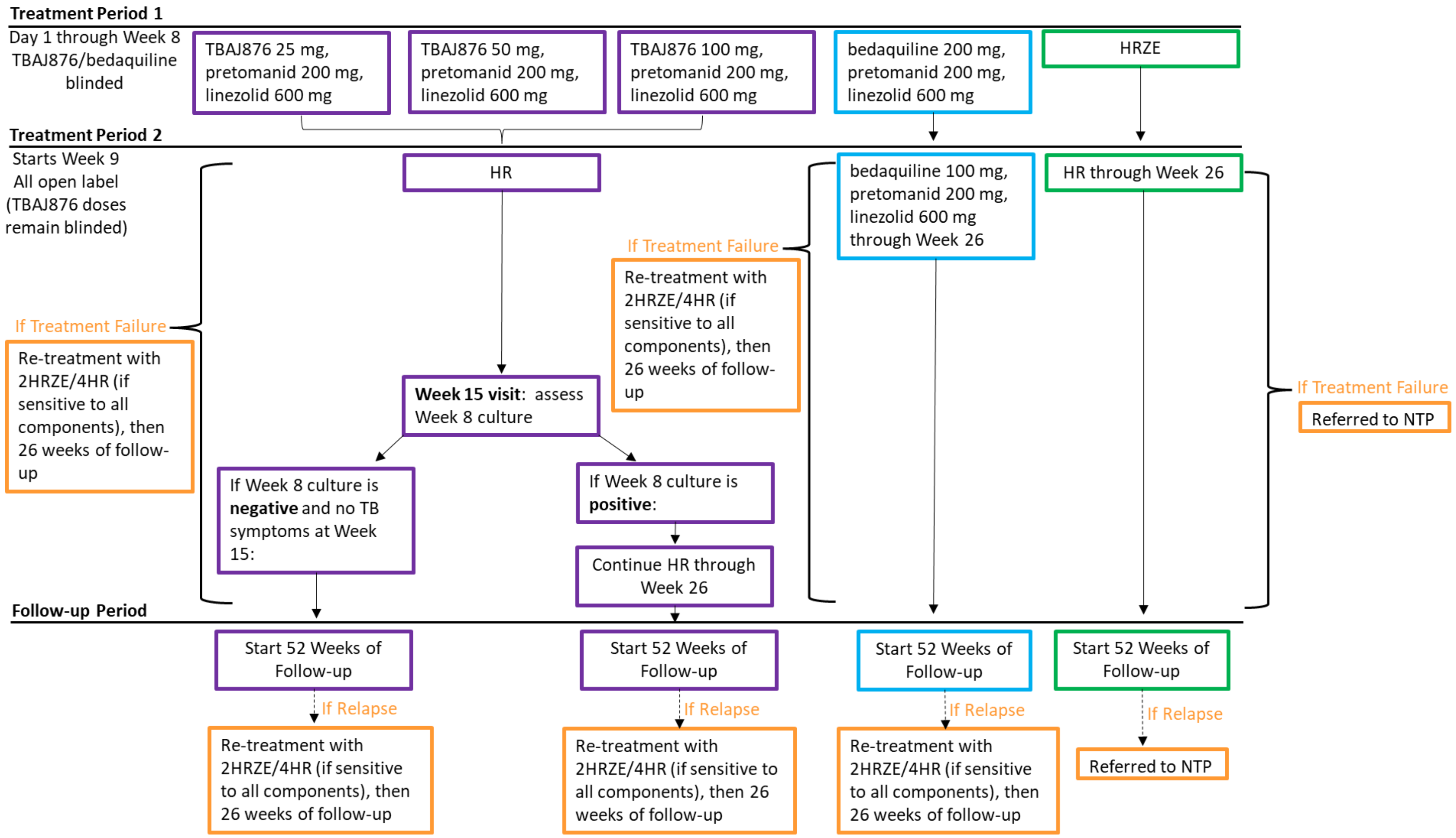

At the Week 15 visit, the Week 8 culture and presence of TB-related symptoms will be assessed for participants who received TBAJ876-Pa-L. If the Week 8 culture is negative, and the participant has no TB-related symptoms by Week 15, the participant can complete treatment at Week 15. Participants with symptoms that have a more likely alternative explanation are eligible to complete treatment at Week 15. If the Week 8 culture is MTB positive and/or the participant has TB symptoms, the participant will continue treatment through Week 26.

Re-Treatment Schematic

Re-treatment 2HRZE/4HR (26 weeks) → **Follow-up Period** 26 weeks after the end of re-treatment

Objectives and Endpoints

Objectives	Endpoints
**Primary**	
To evaluate the efficacy of 3 dose levels of TBAJ876 in combination with pretomanid and linezolid, relative to 2HRZE, during 8 weeks of treatment in adult participants with newly diagnosed, smear-positive, pulmonary DS-TB as measured by time to stable sputum culture conversion to negative status.	Time to stable sputum culture conversion to negative status using data from weekly cultures through 8 weeks of treatment.
**Key Secondary**	
To evaluate the safety and efficacy at 26 weeks after the EOT of B-Pa-L relative to 2HRZE/4HR in adult participants with newly diagnosed, smear-positive, pulmonary DS-TB.	Proportion of participants with a favourable outcome at 26 weeks after the EOT.
**Secondary**	
To evaluate the efficacy of 3 dose levels of TBAJ876 in combination with pretomanid and linezolid, in adult participants with newly diagnosed, smear-positive, pulmonary DS-TB, as measured by meeting the criteria to stop treatment at Week 15.	Proportion of participants who meet the criteria to stop treatment at Week 15.
To evaluate the safety and efficacy of 3 dose levels of TBAJ876 in combination with pretomanid and linezolid at 26 weeks and 52 weeks after end of treatment relative to 2HRZE/4HR for the overall arms and key subgroups by stratification factors and total treatment duration, in adult participants with newly diagnosed, smear-positive, pulmonary DS-TB.	Proportion of participants with a favourable outcome at 26 weeks and 52 weeks after the EOT.
To evaluate the safety and efficacy at 52 weeks after the EOT of B-Pa-L relative to 2HRZE/4HR in adult participants with	Proportion of participants with a favourable outcome at 52 weeks after the EOT.

newly diagnosed, smear-positive, pulmonary DS-TB.	
To evaluate the efficacy of 3 dose levels of TBAJ876 in combination with pretomanid and linezolid, B-Pa-L, and 2HRZE/4HR, at 26 weeks and 52 weeks after the EOT, as measured by relapse rates in participants that enter follow up with a favourable response at the EOT for the overall arms and key subgroups by stratification factors and total treatment duration.	Relapse rates at 26 weeks and 52 weeks after the EOT.
To evaluate the efficacy of B-Pa-L, relative to 2HRZE, during 8 weeks of treatment in adult participants with newly diagnosed, smear-positive, pulmonary DS-TB as measured by time to stable sputum culture conversion to negative status.	Time to stable sputum culture conversion to negative status using data from weekly cultures through 8 weeks of treatment.
To assess the bactericidal activity over 2 weeks of 3 dose levels of TBAJ876 in combination with pretomanid and linezolid or B-Pa-L, relative to 2HRZE, in adult participants with newly diagnosed, smear-positive, pulmonary DS-TB.	BA_{TTP}(1-15) as determined by the rate of change in TTP over Days 1 to 15 of treatment, represented by the model-fitted log(TTP) as calculated by the regression of the observed log(TTP) counts over time.
To assess the bactericidal activity over 8 weeks of 3 dose levels of TBAJ876 in combination with pretomanid and linezolid or B-Pa-L, relative to 2HRZE, in adult participants with newly diagnosed, smear-positive, pulmonary DS-TB.	BA_{TTP}(1-56) as determined by the rate of change in TTP over 8 weeks of treatment, represented by the model-fitted log(TTP) results as calculated by the regression of the observed log(TTP) results over time.
To evaluate stable sputum culture conversion to negative status over time during the treatment period.	Proportion of participants with stable sputum culture conversion to negative status at Weeks 4, 6, 8, 12, 15, 20, and 26.
To assess the safety and tolerability of the 3 dose levels of TBAJ876 in combination with pretomanid and linezolid or B-Pa-L regimens at different time points, relative to 2HRZE/4HR, in adult participants with newly diagnosed, smear-positive, pulmonary DS-TB.	Incidence of TEAEs, by severity, drug relatedness, seriousness, leading to early discontinuation, and leading to death; and ECG, vital signs, and quantitative and qualitative clinical laboratory result measurements, changes in ophthalmic exam for visual acuity and changes noted in peripheral neuropathy, including observed and changes from baseline at Weeks 8, 26, and 26 weeks after the EOT.

To evaluate the systemic exposure of TBAJ876, bedaquiline, pretomanid, and linezolid.	Plasma concentrations of TBAJ876, bedaquiline, pretomanid, linezolid, and selected metabolites from sparse samples in all participants assigned those treatments and from an intensive profile on Day 15 in a sub-group of participants.
**Exploratory**	
To assess if changes in measurements of biomarker assays through the course of treatment and the post-treatment follow-up period are associated with treatment outcome.	Change from baseline in biomarker assay through the course of treatment and the post-treatment follow-up period relative to treatment outcome.
To correlate various endpoints measured with MGIT with AFB smear and exploratory biomarkers at Day 1 and Weeks 4, 8, 15, and 26.	Parameters derived from modelling various endpoints measured with MGIT (e.g. MGIT negative [yes/no], quantitative outcomes) relative to AFB smear and exploratory biomarkers at Day 1 and Weeks 4, 8, 15, and 26
To correlate MGIT culture results at Weeks 2, 4, and 8 with favorable outcome at 26 weeks and 52 weeks after the EOT.	Parameters derived from modelling MGIT culture results at Weeks 2, 4, and 8 (e.g. MGIT negative [yes/no], quantitative outcomes) relative to favourable outcome at 26 weeks and 52 weeks after the EOT.
To explore predictors of MGIT negative culture at Week 8 of treatment.	MGIT negative at Week 8 of treatment (yes/no) relative to key baseline characteristics.
To explore the population PK and exposure-response relationships of TBAJ876, bedaquiline, pretomanid, and linezolid.	Population-PK models, summary metrics of exposure (e.g., AUC) derived from those models, and models for relationships between exposure and efficacy and safety outcomes.
To explore the systemic exposure of dolutegravir and tenofovir when co-administered with 3 dose levels of TBAJ876 in combination with pretomanid and linezolid, B-Pa-L or 2HRZE.	Trough concentrations of dolutegravir and tenofovir from participants living with HIV assigned treatments.
To explore the impact on quality of life of 3 dose levels of TBAJ876 in combination with pretomanid and linezolid, B-Pa-L, and 2HRZE/4HR	Time to improvement of quality of life measurements, proportion of participants with improved measurements on quality of life at different time points during and after treatment.

Committee Membership

All members of the DSMC will be independent from the trial and the sponsor, the TB Alliance (TBA), and have no financial, scientific, or other conflicts of interest with the trial. The DSMC will be composed of at least 3 voting members (at least 2 of whom are Physicians), and 1 member, a

Statistician. The minimum total of 4 DSMB members should collectively have the following expertise/qualifications:

1. TB/Infectious Disease Specialist
2. Physician/Trialist
3. Statistician
4. Country representative/s where required.

All DSMC members should complete Appendix 1: Member Signature Page and provide a copy to the Clinical Project Manager on finalisation of the DSMC Charter. Additionally, each DSMC member will be asked to sign a 'TB Alliance Conflicts of Interest and Confidentiality form'.

The Rho (a CRO partner of the TBA) Unblinded Statistician will oversee the collation and provision of the required documentation to the DSMC and will also attend both the open and closed sessions of the DSMC Meeting. It is recommended that the Study Physician, Medical Monitors, TB Alliance Clinical Project Manager, Chief Medical Officer, and the statistician(s) attend the open session of the DSMC Meeting. The Study Physician may invite other staff from TBA/the CRO study teams to the meeting, at their discretion. Meetings may be either in person or by teleconference. Refer to section 6 of the DSMC Charter for details on the open and closed sessions of the meeting.

Every effort will be made to maintain the continuity of the membership in this committee. Should a DSMC member leave the committee, TBA is responsible for selecting a replacement DSMC member. The replacement will have appropriate and preferably similar expertise to the DSMC member who resigned from the committee. The departing member is to return all study-related documents to the Chairperson and the incoming member will receive all information about previous DSMC activities from the DSMC Chairperson.

The DSMC for this trial will consist of the following members:

1. **Chairperson:** **Prof. Nick Paton**
   Affiliation: National University of Singapore
2. **Statistician:** **Dr Daniel Grint**
   Affiliation: London School of Hygiene and Tropical Medicine
3. **TB Disease Specialist:** **Prof. Stephen Gillespie**
   Affiliation: University of St Andrews
4. **Physician:** **Prof. Susan Dorman**
   Affiliation: Medical University of South Carolina, Division of Infectious Disease
5. **Country Representative:** **Prof. Muhammad Bakari Kambi (Representative from Tanzania)**

Affiliation: Muhimbili University of Health and Allied Sciences (MUHAS)

The Chairperson is the contact person for the DSMC, and primary communication between the DSMC and TBA will be routed through the Chairperson and the TBA Study Physician.

Roles & Responsibilities

The DSMC will act in an advisory capacity to TBA, to safeguard the interest of trial participants by monitoring participant safety, participant risk versus benefit, and general evaluation of study progress.

Specifically, the DSMC will:

- After receipt from the Rho Unblinded Statistician, review the pre-meeting documentation provided (details described in DSMC Charter section 7).
- Report to TBA on the safety and progress of the trial.
- Advise TBA if the trial should continue without modification, be modified, or be terminated following their review meeting, which will occur approximately every six months after the first participant is randomised.
- Consider factors external to the study when relevant information becomes available, such as published scientific or new non-clinical findings that may be relevant to human safety that may have an impact on the safety of the participants or the ethics of the trial. As required, consider regulatory and/or ethics issues raised and make recommendations to TBA on appropriate management strategies.
- Ensure confidentiality of all study data, the results of the DSMC discussions, and recommendations.
- Assist TBA by commenting on any problems with study conduct, recruitment, sample size and/or data collection and/or analyses, if required.
- Maintain applicable meeting records (responsibility of the Rho Unblinded Statistician).

The DSMC will discharge itself from its duties when the study is complete.

Confidentiality

All materials, discussions, and proceedings of the DSMC are completely confidential. Members and other participants in DSMC meetings are expected to maintain confidentiality.

Meeting Format & Frequency

The CPM (Clinical Project Manager) and or designee will arrange a Kick-off DSMC meeting prior to the randomization of the first participant, to review the trial design and the draft DSMC Charter. After this meeting the DSMC Charter will be finalised and all DSMC members will complete and sign the 'Member Signature Page'.

A DSMC meeting will be held approximately every 6 months after the first randomised participant. Ad-hoc meetings can be called by TBA or the DSMC based on the rates of SAEs, SAEs of particular concern,

or any safety concerns that arise during the trial. The Study Physician will discuss any interim safety concerns with the Chairperson and together agree on whether an ad hoc meeting of the DSMC is needed.

The Kick-off DSMC meeting should establish the following:

- Exact data (Tables, Figures, and Listings) and format that should be reviewed during the open and closed sessions.
- Timing of communication of SAEs, AESIs, including deaths

The Rho Unblinded Statistician, in consultation with the Clinical Project Manager and or designee and DSMC Chairperson, will:

- Schedule the 6-monthly DSMC meetings (date and time)
- Prepare and distribute the meeting agenda and materials to all attendees, in advance of meeting.
- Upon receipt from the DSMC Chairperson, distribute the DSMC recommendation letter to all relevant parties, as detailed in Section 8 of the DSMC Charter.

Meetings to review data may be in person or by teleconference. The DSMC meetings to review data should consist of an open and closed session:

The open session (including, but not limited to, operational status and key updates): can be scheduled just prior to the closed session and will provide opportunity for the Clinical Project Manager, Study Physician, Medical Monitor and/or Chief Medical Officer to present the progress of the study, participant accrual, protocol compliance, and any other relevant issues. External information from other trials may also be presented.

The closed session (sponsor will not attend this session): in addition to the information/material provided in the open session, information/material for the closed session will include unblinded safety data and efficacy data as agreed with the DSMC. The purpose of the DSMC's review of the efficacy data is to assess the benefit/risk context for potential safety concerns. This session is to be used to discuss and agree on recommendations for the trial.

The meeting must include a recommendation to continue, terminate or modify the study made by consensus, or if necessary, a majority vote.

Should the DSMC decide to issue a *termination* recommendation, every effort should be made for all DSMC members to vote on the recommendation. If required, the Rho Unblinded Statistician should schedule a meeting between the DSMC Chairperson and TBA representative to discuss the recommendation. Ultimately TBA will make the decision on whether the trial is to be terminated.

Should the DSMC decide to issue a recommendation *to continue with or modify the trial*, a majority vote of the DSMC is required. Should there not be a majority vote in favour of the recommendation and a tie is reached, the vote of the DSMC Chairperson will serve as the final decision. If required, the

Rho Unblinded Statistician should schedule a meeting between the DSMC Chairperson and a TBA representative to discuss the recommendations.

The DSMC Chairperson will make every effort to ensure the participation of every member in meetings. A quorum will consist of at least 3 members of the DSMC. If only 2 members can meet for an urgent assessment of safety data and their vote is split on a recommendation to continue with or modify the trial, a meeting will be scheduled with TBA to discuss their recommendations.

Meeting Documentation

Pre-meeting Documentation

After sending out a meeting request and providing the Meeting Agenda, the Rho Unblinded Statistician will collect and collate all the required pre-meeting materials. Documentation should be circulated to the appropriate parties (agenda and open session materials to all attendees, closed session materials to DSMC members only) at least ***5 to 10 working days before the meeting*** will take place. The location and availability of meeting materials should be communicated to the DSMC members, by the Rho Unblinded Statistician, in advance of the meeting.

Documentation to be distributed prior to the meeting:

Open Session:

- Study Protocol and Amendments/Administrative Changes as applicable
- DSMC Charter
- Current Medical Monitoring Plan
- Current Informed Consent(s)
- Investigator Brochures / Package Inserts
- Executive Summary (at a minimum to include a summary of participant recruitment (screening and randomization status'), withdrawal, completion and lost to follow up; protocol deviations; performance of the trial sites; other relevant study issues)
- Any other factors that could influence participant safety and study outcome (e.g., data from other trials, regulatory warnings etc.).
- Open displays as agreed with the DSMC

Closed Session:

- Closed displays as agreed with the DSMC

Rho Unblinded Statistician to generate:

- Participant disposition
- Baseline demographic characterization

- History of dosing/ dose interruptions and reductions
- Safety data per DSMC approved Tables, Figures and Listings
- Microbiology data and efficacy information, as it relates to the safety assessment.
- Participant profiles

Rho Pharmacovigilance function to provide:

- SAE CIOMS

In advance of the meeting to review data, TBA will propose to and agree with, the DSMC members on the specific data elements and format of the data that will be presented for review.

Post-meeting documentation

The DSMC Chairperson will prepare the DSMC Recommendation Letter (Appendix 2) within 10 (ten) working days of the meeting and provide it to the Rho Unblinded Statistician who will send the recommendation within one working day to the Study Physician and the Clinical Project Manager.

Documentation of the open and closed meeting will comprise of the:

- Agenda;
- Attendance Register;
- DSMC Recommendation Letter.

Meeting summaries (including decisions made and action items) will be documented for both the open and closed meetings. The Rho Unblinded Statistician will document the meeting summaries of the closed meetings and the DSMC Chairperson will document the Recommendation Letter.

Meeting summaries of the closed meetings should not detail the per member voting decision.

These should be distributed within 10 (ten) working days of the DSMC Meeting. The TBA Clinical Project Manager will document, distribute and finalise the meeting summaries of the open meetings.

All meeting documentation will be retained by the Rho Unblinded Statistician until the end of trial, where after it will be provided to TBA/the CRO for inclusion in the Trial Master File.

DSMC Recommendation Letter & Distribution

A formal letter containing the recommendations for continuation, termination or modification of the study will be prepared and signed by the DSMC Chairperson. Refer to Appendix 2 for the template which may be used for the recommendation letter.

a) Should the DSMC decide to issue a recommendation to *terminate or to modify the trial*:

The DSMC Chairperson should provide such a recommendation to the Study Physician by telephone and e-mail within 2 (two) working days after the meeting. The formal recommendation letter (Appendix 2) should be included in the e-mail and the DSMC members should be copied on the correspondence. Once the DSMC has made a formal and written recommendation, TBA will notify the participating vendors, as appropriate.

It is the responsibility of TBA to ensure that appropriate Regulatory Authorities, Investigators and Ethics Committees are informed, as required.

b) Should the DSMC decide to issue a recommendation *to continue with the trial*:

The DSMC Chairperson will prepare the DSMC Recommendation Letter (Appendix 2) within 10 (ten) working days of the meeting and provide it to the Rho who will send the recommendation within one working day to the Study Physician and the Clinical Project Manager.

Appendix 1: DSMC Member Signature Page

Data Safety Monitoring Committee Charter

MEMBER SIGNATURE PAGE

Protocol Title: A phase 2, partially-blinded, randomised trial assessing the safety and efficacy of TBAJ876 or bedaquiline, in combination with pretomanid and linezolid in adult participants with newly diagnosed, drug-sensitive, smear-positive pulmonary tuberculosis

Protocol Number: NC-009

Version and Date: 1.0 03FEB2023

MEMBER INFORMATION

**Name:**		
**Qualifications:**		
**Address:**		
**Contact Details:**	**Tel:**	
	**Cell:**	
	**E-mail**	

Role: DSMC Chair ☐ Member ☐

Voting Rights: Yes ☐ No ☐

I have reviewed the above version of the DSMC Charter and approve it as written. I understand my role as a member of this DSMC.

Name and Title:

Signature and Date:

Appendix 2: Template NC-009 DSMC Recommendation Letter

<<CPM to populate this letter in advance of the DSMC Meetings and email it to the Chairperson>>

<<Insert Date>>

To: NC-009 Study Physician
TB Alliance

cc: All DSMC Members

Dear Dr Olugbosi

The Date Safety Management Committee (DSMC) for the NC-009 trial met on *<<insert date>> <<at xxx>> <<or via teleconference>>* to review the trial progress and interim accumulating safety data.

The following DSMC members attended the meeting, reviewed the data and approved the recommendation:

<< Delete members which were not present>>

- Prof. Nick Paton (Chairperson)
- Dr Daniel Grint (Statistician)
- Prof. Stephen Gillespie (TB Disease Specialist)
- Prof. Susan Dorman (Physician)
- Prof. Muhammad Bakari Kami (Country Representative from Tanzania)

The following documents were reviewed and discussed during the open session as applicable:

<<Edit table and document description, per trial requirements>>

Document Description	Version	Date	Update implemented since last DSMC meeting?
Protocol	1.0	3 Feb 2023	NA
Protocol Memo #1	1.0	30 May 2023	NA
Protocol Memo #2	1.0	2 Jun 2023	NA
Protocol Memo #3 (Uganda Specific)	1.0	3 Jul 2023	NA
Protocol Memo #4 (Uganda and Tanzania Specific)	1.0	13 Jul 2023	NA
Protocol Memo #5	1.0	24 Jul 2023	NA
Protocol Memo #6	1.0	27 Sep 2023	NA
Medical Monitoring Plan	1.0	27 Sep 2023	NA
Informed consent – Main	1.0	16 Jun 2023	NA
Informed consent – HIV	0.1	3 Feb 2023	NA
Informed consent – 24Hr PK	0.1	16 Feb 2023	NA
Informed consent – Pharmacogenetic	1.0	13 Jun 2023	NA
Informed consent – Biostorage / Exploratory	1.0	2 Jun 2023	NA
Investigator Drug Brochure, PA-824	22	31 Jan 2023	NA

Investigator Drug Brochure, TBAJ876	3.0	16 Dec 2022	NA
Investigator Drug Brochure, Bedaquiline	16	28 Oct 2022	NA
Investigator Drug Brochure, Linezolid	3.0	7 Feb 2023	NA
Package insert, HRZE HR	NA	Jul 2011	NA
DSMC Charter	1.0	27 Sep 2023	NA

The following participant data was discussed during the closed session:

- Disposition, demographic information, and study medication dosing history
- Safety Data and ECG results
- Microbiology data as it relates to the safety assessment.
- *<<list of other data>>*

As a result, the DSMC recommendation is (indicate appropriate action):

☐ *To continue study unmodified*

☐ *To continue study unmodified and plan an additional meeting.*

☐ *To continue study unmodified, and request additional data/expert review/analyses.*

Describe and provide timelines of additional review:

☐ *To continue study and amend protocol(s) as described:*

Describe sections below and list protocol(s) to be amended.

☐ *To set up a meeting with TBA to discuss concerns of safety and/or efficacy within the clinical study as outlined below, within [#] weeks after the DSMC meeting.*

Yours sincerely,

____

Prof. Nick Paton

Chairperson of the NC-009 Data Safety Management Committee

On behalf of the DSMC (members listed below):

- Dr Daniel Grint (Statistician)
- Prof. Stephen Gillespie (TB Disease Specialist)
- Prof. Susan Dorman (Physician)
- Prof. Muhammad Bakari Kami (Country Representative from Tanzania)

DocuSign

Certificate Of Completion

Envelope Id: 6A0FEB2A64B0426CB30B93EBD6009F99		Status: Completed
Subject: Complete with DocuSign: NC-009 DSMC Charter V1.0 27 Sep 2023.docx		
Source Envelope:		
Document Pages: 18	Signatures: 3	Envelope Originator:
Certificate Pages: 5	Initials: 0	Leandra Lombard
AutoNav: Enabled		80 Pine Street, FL 20
EnvelopeId Stamping: Disabled		New York, NY 10005
Time Zone: (UTC-08:00) Pacific Time (US & Canada)		Leandra.Lombard@tballiance.org
		IP Address: 102.132.134.147

Record Tracking

Status: Original<br>27 September 2023 | 04:29	Holder: Leandra Lombard<br>Leandra.Lombard@tballiance.org	Location: DocuSign

Signer Events	Signature	Timestamp
Leandra Lombard<br>leandra.lombard@tballiance.org<br>Director, Clinical Operations<br>Global Alliance for TB Drug Development (Part 11 Compliant)<br>Security Level: Email, Account Authentication (Required)	Leandra Lombard<br><br>Signature Adoption: Pre-selected Style<br>Signature ID:<br>4937F95D-E43E-4606-86F6-75BCCD798AA0<br>Using IP Address: 102.132.134.147<br><br>With Signing Authentication via DocuSign password<br>With Signing Reasons (on each tab):<br>I am the author of this document	Sent: 27 September 2023 | 04:33<br>Viewed: 27 September 2023 | 04:33<br>Signed: 27 September 2023 | 04:34
**Electronic Record and Signature Disclosure:**<br>Not Offered via DocuSign		
Morounfolu Olugbosi<br>morounfolu.olugbosi@tballiance.org<br>Senior Director, Clinical Development<br>TB Alliance<br>Security Level: Email, Account Authentication (Required)	Morounfolu Olugbosi<br><br>Signature Adoption: Pre-selected Style<br>Signature ID:<br>3DABF6C3-410E-4B8F-85E1-D6E511FA5A5F<br>Using IP Address: 102.132.181.230<br><br>With Signing Authentication via DocuSign password<br>With Signing Reasons (on each tab):<br>I approve this document	Sent: 27 September 2023 | 04:33<br>Viewed: 27 September 2023 | 04:43<br>Signed: 27 September 2023 | 04:43
**Electronic Record and Signature Disclosure:**<br>Accepted: 27 September 2017 | 05:45<br>ID: 8028570c-617d-4406-b419-ac9d8a5cc392		

Signer Events	Signature	Timestamp
Nick Paton<br>nick_paton@nus.edu.sg<br>Security Level: Email, Account Authentication (Required)	Nick Paton<br><br>Signature Adoption: Pre-selected Style<br>Signature ID:<br>DAC57715-F6BD-41C3-A784-86A47F80F306<br>Using IP Address: 194.73.19.51<br><br>With Signing Authentication via DocuSign password<br>With Signing Reasons (on each tab):<br>I approve this document	Sent: 27 September 2023 | 04:33<br>Viewed: 28 September 2023 | 05:31<br>Signed: 28 September 2023 | 05:34

Electronic Record and Signature Disclosure:
Accepted: 28 September 2023 | 05:31
ID: 09e2dc42-506c-4fc9-900d-33521d97f1fb

In Person Signer Events	Signature	Timestamp

Editor Delivery Events	Status	Timestamp

Agent Delivery Events	Status	Timestamp

Intermediary Delivery Events	Status	Timestamp

Certified Delivery Events	Status	Timestamp

Carbon Copy Events	Status	Timestamp

Witness Events	Signature	Timestamp

Notary Events	Signature	Timestamp

Envelope Summary Events	Status	Timestamps
Envelope Sent	Hashed/Encrypted	27 September 2023 | 04:33
Certified Delivered	Security Checked	28 September 2023 | 05:31
Signing Complete	Security Checked	28 September 2023 | 05:34
Completed	Security Checked	28 September 2023 | 05:34

Payment Events	Status	Timestamps

Electronic Record and Signature Disclosure

ELECTRONIC RECORD AND SIGNATURE DISCLOSURE

From time to time, Global Alliance for TB Drug Development-Sub Account (we, us or Company) may be required by law to provide to you certain written notices or disclosures. Described below are the terms and conditions for providing to you such notices and disclosures electronically through your DocuSign, Inc. (DocuSign) Express user account. Please read the information below carefully and thoroughly, and if you can access this information electronically to your satisfaction and agree to these terms and conditions, please confirm your agreement by clicking the 'I agree' button at the bottom of this document.

Getting paper copies

At any time, you may request from us a paper copy of any record provided or made available electronically to you by us. For such copies, as long as you are an authorized user of the DocuSign system you will have the ability to download and print any documents we send to you through your DocuSign user account for a limited period of time (usually 30 days) after such documents are first sent to you. After such time, if you wish for us to send you paper copies of any such documents from our office to you, you will be charged a $0.00 per-page fee. You may request delivery of such paper copies from us by following the procedure described below.

Withdrawing your consent

If you decide to receive notices and disclosures from us electronically, you may at any time change your mind and tell us that thereafter you want to receive required notices and disclosures only in paper format. How you must inform us of your decision to receive future notices and disclosure in paper format and withdraw your consent to receive notices and disclosures electronically is described below.

Consequences of changing your mind

If you elect to receive required notices and disclosures only in paper format, it will slow the speed at which we can complete certain steps in transactions with you and delivering services to you because we will need first to send the required notices or disclosures to you in paper format, and then wait until we receive back from you your acknowledgment of your receipt of such paper notices or disclosures. To indicate to us that you are changing your mind, you must withdraw your consent using the DocuSign 'Withdraw Consent' form on the signing page of your DocuSign account. This will indicate to us that you have withdrawn your consent to receive required notices and disclosures electronically from us and you will no longer be able to use your DocuSign Express user account to receive required notices and consents electronically from us or to sign electronically documents from us.

All notices and disclosures will be sent to you electronically

Unless you tell us otherwise in accordance with the procedures described herein, we will provide electronically to you through your DocuSign user account all required notices, disclosures, authorizations, acknowledgements, and other documents that are required to be provided or made available to you during the course of our relationship with you. To reduce the chance of you inadvertently not receiving any notice or disclosure, we prefer to provide all of the required notices and disclosures to you by the same method and to the same address that you have given us. Thus, you can receive all the disclosures and notices electronically or in paper format through the paper mail delivery system. If you do not agree with this process, please let us know as described below. Please also see the paragraph immediately above that describes the consequences of your electing not to receive delivery of the notices and disclosures electronically from us.

How to contact Global Alliance for TB Drug Development-Sub Account:
You may contact us to let us know of your changes as to how we may contact you electronically, to request paper copies of certain information from us, and to withdraw your prior consent to receive notices and disclosures electronically as follows:
To contact us by email send messages to: christi.baine@tballiance.org

To advise Global Alliance for TB Drug Development-Sub Account of your new e-mail address
To let us know of a change in your e-mail address where we should send notices and disclosures electronically to you, you must send an email message to us at christi.baine@tballiance.org and in the body of such request you must state: your previous e-mail address, your new e-mail address. We do not require any other information from you to change your email address..
In addition, you must notify DocuSign, Inc to arrange for your new email address to be reflected in your DocuSign account by following the process for changing e-mail in DocuSign.
To request paper copies from Global Alliance for TB Drug Development-Sub Account
To request delivery from us of paper copies of the notices and disclosures previously provided by us to you electronically, you must send us an e-mail to christi.baine@tballiance.org and in the body of such request you must state your e-mail address, full name, US Postal address, and telephone number. We will bill you for any fees at that time, if any.
To withdraw your consent with Global Alliance for TB Drug Development-Sub Account
To inform us that you no longer want to receive future notices and disclosures in electronic format you may:

i. decline to sign a document from within your DocuSign account, and on the subsequent page, select the check-box indicating you wish to withdraw your consent, or you may;
ii. send us an e-mail to christi.baine@tballiance.org and in the body of such request you must state your e-mail, full name, IS Postal Address, telephone number, and account number. We do not need any other information from you to withdraw consent.. The consequences of your withdrawing consent for online documents will be that transactions may take a longer time to process..

Required hardware and software

Operating Systems:	Windows2000? or WindowsXP?
Browsers (for SENDERS):	Internet Explorer 6.0? or above
Browsers (for SIGNERS):	Internet Explorer 6.0?, Mozilla FireFox 1.0, NetScape 7.2 (or above)
Email:	Access to a valid email account
Screen Resolution:	800 x 600 minimum
Enabled Security Settings:	•Allow per session cookies<br>•Users accessing the internet behind a Proxy Server must enable HTTP 1.1 settings via proxy connection

** These minimum requirements are subject to change. If these requirements change, we will provide you with an email message at the email address we have on file for you at that time providing you with the revised hardware and software requirements, at which time you will

have the right to withdraw your consent.

Acknowledging your access and consent to receive materials electronically

To confirm to us that you can access this information electronically, which will be similar to other electronic notices and disclosures that we will provide to you, please verify that you were able to read this electronic disclosure and that you also were able to print on paper or electronically save this page for your future reference and access or that you were able to e-mail this disclosure and consent to an address where you will be able to print on paper or save it for your future reference and access. Further, if you consent to receiving notices and disclosures exclusively in electronic format on the terms and conditions described above, please let us know by clicking the 'I agree' button below.

By checking the 'I Agree' box, I confirm that:

- I can access and read this Electronic CONSENT TO ELECTRONIC RECEIPT OF ELECTRONIC RECORD AND SIGNATURE DISCLOSURES document; and

- I can print on paper the disclosure or save or send the disclosure to a place where I can print it, for future reference and access; and

- Until or unless I notify Global Alliance for TB Drug Development-Sub Account as described above, I consent to receive from exclusively through electronic means all notices, disclosures, authorizations, acknowledgements, and other documents that are required to be provided or made available to me by Global Alliance for TB Drug Development-Sub Account during the course of my relationship with you.
